# Cyclic ester insertion into a bismuth–amide bond: isolable molecular models for ROP initiation

**DOI:** 10.1039/d6cc05601f

**Published:** 2026-09-21

**Authors:** Gabija Navickaite, Louis J. Morris, Alexander Evans, Zoë R. Turner, Dermot O’Hare

**Affiliations:** a Chemistry Research Laboratory, Department of Chemistry, University of Oxford, 12 Mansfield Road Oxford OX1 3TA UK zoe.turner@earth.ox.ac.uk dermot.ohare@chem.ox.ac.uk

## Abstract

Reaction of a bismuth amide with l-lactide, ε-caprolactone and γ-butyrolactone gives isolable ring-opened products which are also observed during polymerisation studies, including rare crystallographically authenticated amidolactate and γ-butyrolactone-derived complexes. Monomer-dependent divergence between post-insertion transesterification, delayed propagation and arrested insertion provides insight into ROP initiation.

## Abstract 2

Bi–N bonds ring-open cyclic esters to yield isolable complexes as characterised snapshots of ROP initiation species.

## Graphical abstract

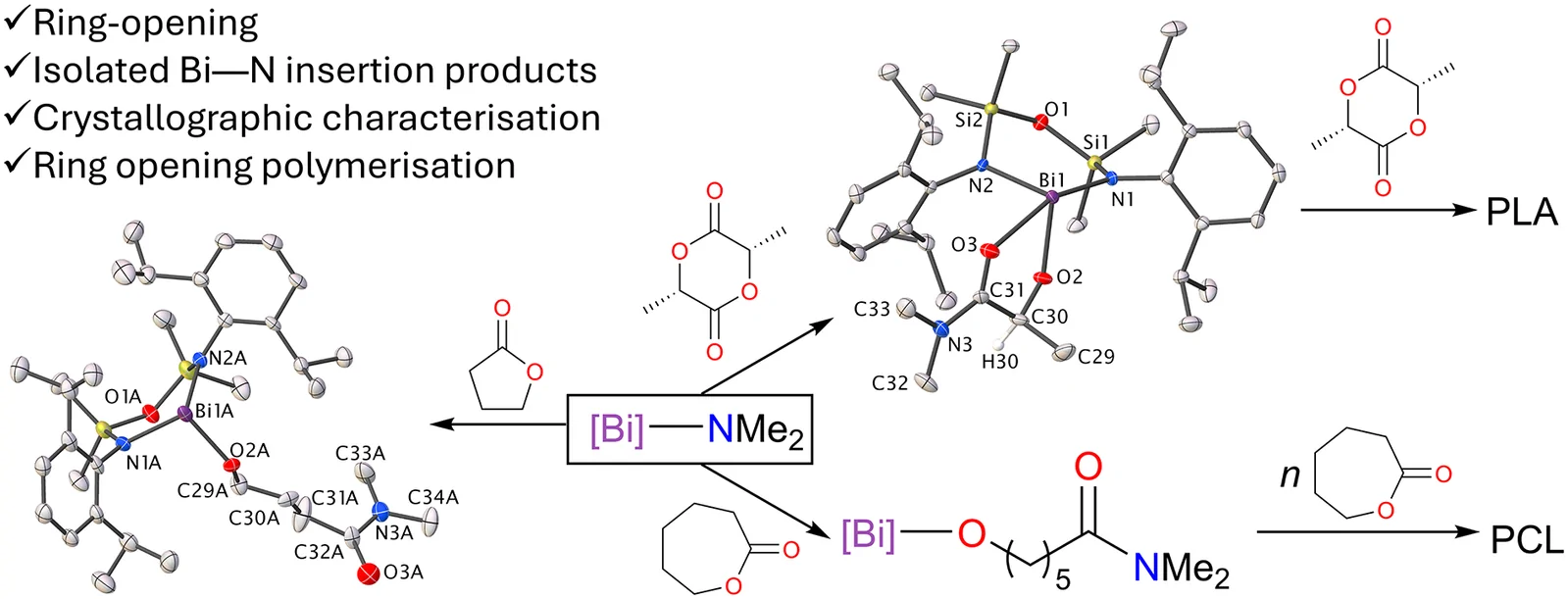

The ring opening polymerisation (ROP) of cyclic esters produces biodegradable and biocompatible polymers such as poly(lactic acid) and poly(ε-caprolactone) with applications in packaging, fibres, and medicine on an industrial scale.^1–10^ One of the most common mechanisms for the ROP of cyclic esters is the coordination-insertion mechanism, where nucleophilic attack of an initiating group on a metal-coordinated monomer yields an insertion product as the propagating species. Compounds formed during the first monomer insertion event have been shown to be representative of propagating species.^11–17^ Whilst crystallographically characterised lactate M{OCH(Me)C(O)R} and lactyl lactate M{OCH(Me)C(O)OCH(Me)C(O)R} complexes, including bismuth derivative I (Chart 1), have been studied as models for the propagating species in the ROP of lactide,^11,15,18–28^ only four structurally authenticated examples formed by ring-opening the genuine monomer have been reported (II–V, Chart 1).^14,15,29,30^ Further, whilst numerous structurally characterised adducts of lactones have been reported,^29,31–36^ model compounds for the propagating intermediates in lactone ROP are rare^20^ and, to our knowledge, structurally characterised insertion products derived from ring-opened lactones are entirely absent from the literature.

**Chart 1:**
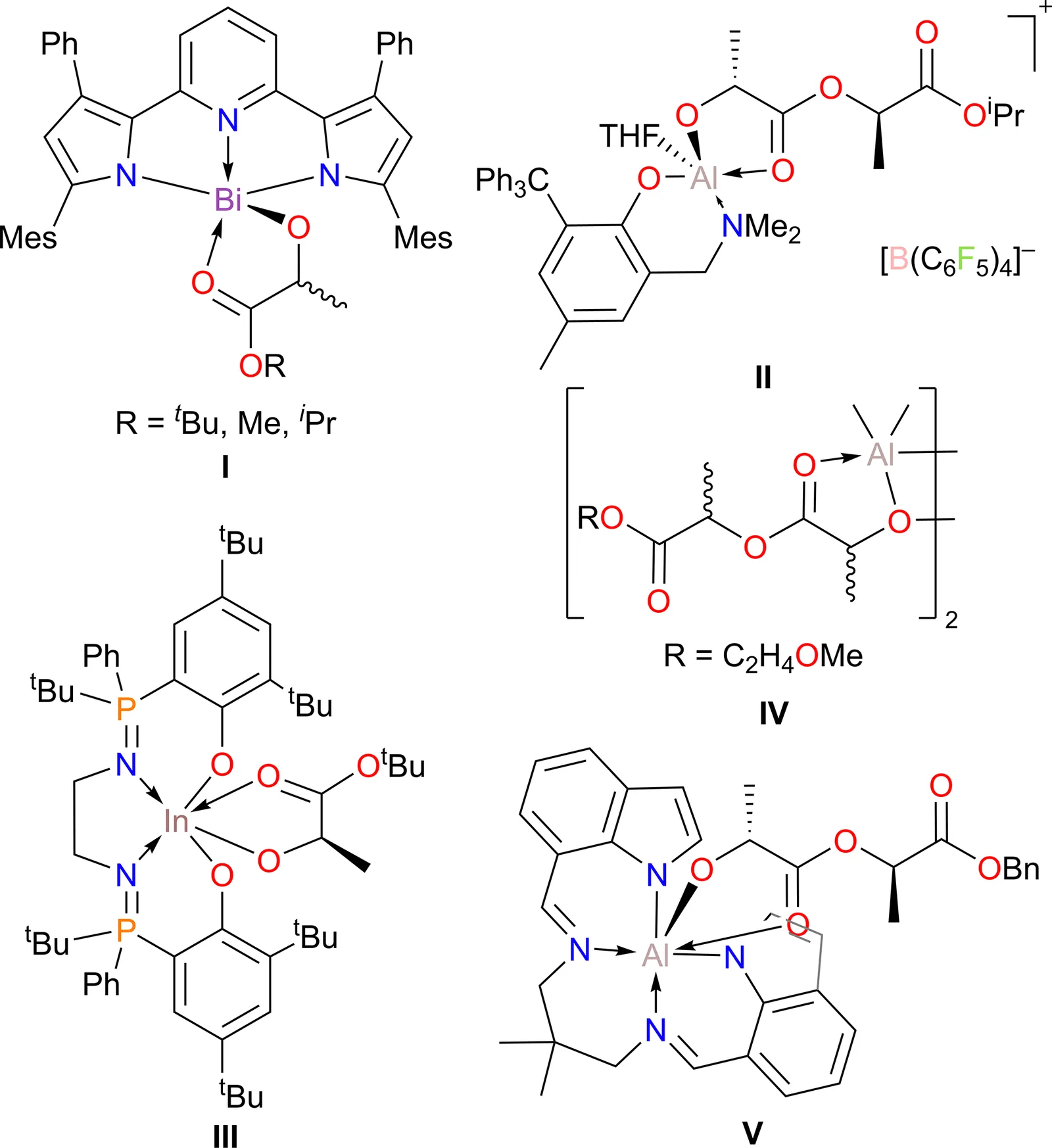
Previously reported bismuth lactyl complex I, and structurally characterised metal lactyl lactate and lactate compounds (II–V) derived from the ring-opening of a lactide monomer.

Bismuth is the heaviest non-radioactive element,^37^ yet it is non-toxic and has found applications in cosmetics and pharmaceuticals,^38^ making it an attractive choice for the development of non-toxic^10,39–41^ ROP initiators.^11,42–44^ Bismuth amide complexes are nucleophilic reagents with well-established reactivity including the insertion of electrophilic small molecules into the Bi–N bond,^45–52^ intramolecular C–H activation,^52–54^ and radical reactivity derived from Bi–N homolysis.^55^ With this in mind, we surmised that the controlled insertion of cyclic esters into a Bi–N bond may provide direct access to isolable products representative of the species formed during ROP initiation.

To this end, and inspired by Coles, Fulton, and co-workers use of the di(amido)siloxane ligand [O(SiMe_2_NDipp)_2_]^2−^ ([NON^Dipp^]^2−^; Dipp = 2,6-di-isopropyl-phenyl) to access bismuth-based bond activation chemistry,^56–59^ the bismuth amide complex [(NON^Dipp^)Bi(NMe_2_)] (1) was synthesised by protonolysis of [Bi(NMe_2_)_3_] with NON^Dipp^H_2_ (see SI for details). The crystal structure of 1 reveals a trigonal pyramidal bismuth centre (Σ(∠Bi) = 297.35°), chelated by the [NON^Dipp^]^2−^ ligand in a κ^2^-*N*,*N*′-bidentate bonding mode (Scheme 1b). The BiN_2_Si_2_O metallacycle adopts a half-chair conformation and the Bi–N bond length of the NMe_2_ moiety (Bi1–N3 = 2.112(2) Å) compares well with the Bi–N distances reported for related bismuth dimethylamide compounds (2.050(14)–2.189(18) Å).^48,49,60^ The ^1^H NMR spectrum of 1 (Fig. S1) displays two inequivalent SiMe_2_ proton environments at *δ* 0.44 and 0.27 ppm, consistent with *C*_s_-symmetry, whilst the NMe_2_ group resonates as a singlet at *δ* 3.78 ppm.

**Scheme 1:**
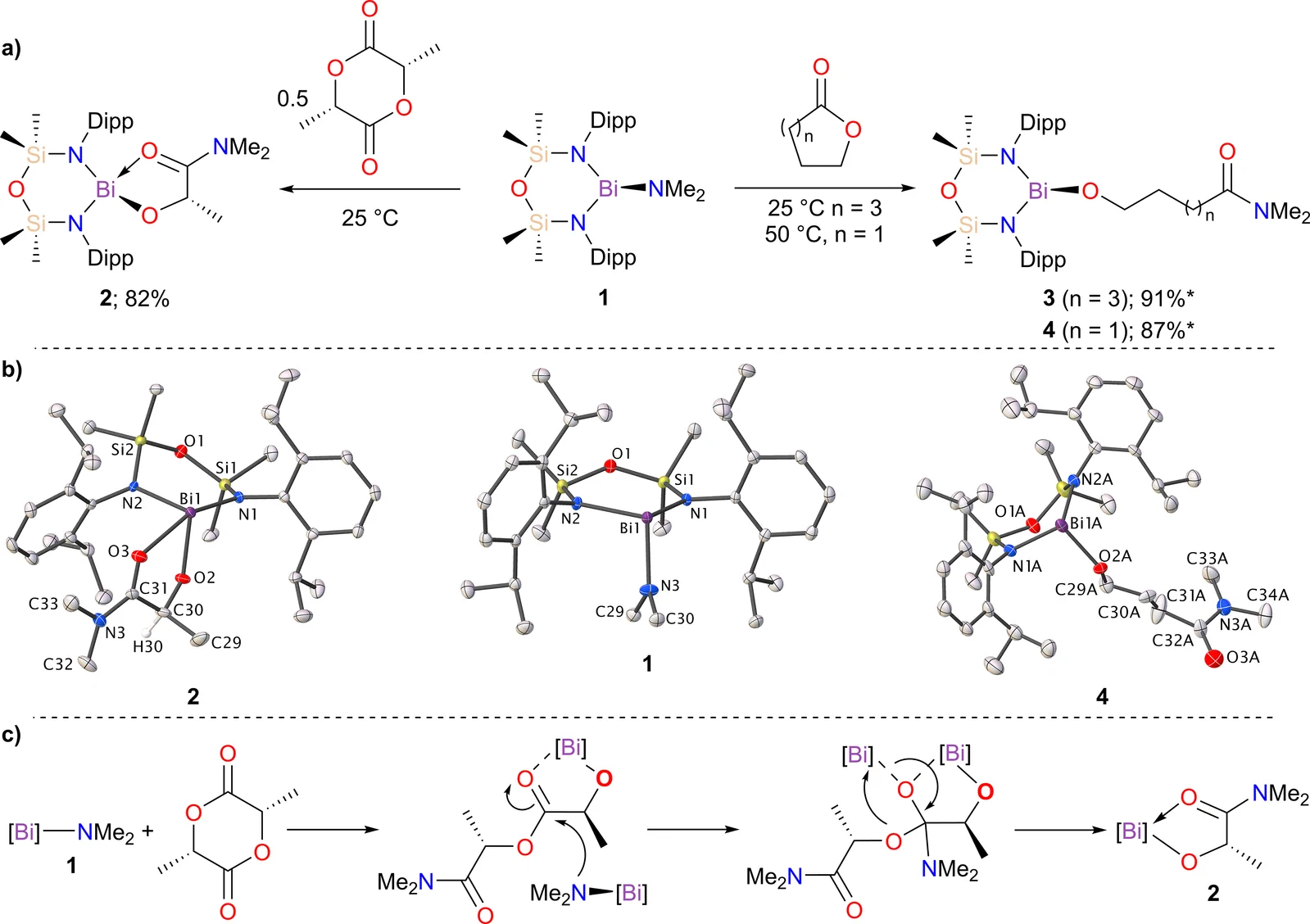
(a) Synthesis of compounds 2–4; (b) displacement ellipsoid drawings (30% probability) of the X-ray crystal structures of 1, 2, and 4; (c) postulated mechanism for the formation of 2. Reactions carried out using benzene as reaction solvent. Isolated yield reported for 2. *Crude isolated yield reported for 3 and 4 due to contamination with residual lactone. Dipp = 2,6-iPr-C_6_H_3_. All hydrogen atoms have been omitted from the displacement ellipsoid drawings for clarity, except for H30 in compound 2. Structure of 4 is disordered and the major component only is shown for atomic connectivity. Compound 4 displays intermolecular Bi1A–O3A/Bi1B–O3B interactions in the solid-state, see SI (Fig. S65–S69).

The addition of a half an equivalent of l-lactide (l-LA) to a benzene solution of 1 resulted in rapid consumption of the starting complex and formation of a new chiral *C*_1_-symmetric species, 2, identified in the *in situ*^1^H NMR spectrum (Fig. S3, S9) by four singlets corresponding to the Si*Me*_2_ protons and four septets assigned to the C*H*Me_2_ protons (Scheme 1a). Further, the N*Me*_*2*_ resonance of 1 was replaced by two singlets at *δ* 2.04 and 1.83 ppm in the ^1^H NMR spectrum, whilst a mutually coupled doublet and quartet (^3^*J*_H–H_ = 6.6 Hz; *δ* 0.71, 6.22 ppm, respectively) were assigned to the CH*Me* and C*H*Me protons originating from l-LA. Consistent with insertion of l-LA into the Bi–N bond, the latter resonance is significantly shifted to higher frequency relative to l-LA (*δ*_H_ (C*H*Me) 3.76 ppm), as is the carbonyl resonance in the ^13^C{^1^H} NMR spectrum (Fig. S5; *δ* 182.6 ppm, *cf.* 167.0 ppm for l-LA). Subsequent X-ray diffraction analysis of a colourless single crystal confirmed the structure of 2 as a bismuth amidolactate [(NON^Dipp^)Bi(κ^2^-*O*,*O*-OCH(Me)C(O)NMe_2_)] (Scheme 1b). The formation of 2 is proposed to proceed *via* the ring-opening of l-LA by nucleophilic attack of the NMe_2_ group followed by rapid bimolecular transesterification of a putative lactamido lactate intermediate (Scheme 1c). Intermolecular transesterification during the formation of metal lactate compounds has been previously suggested for tin and bismuth initiators.^11,61^

In the crystal structure of 2 (space group *P*2_1_2_1_2_1_), the bismuth centre adopts a distorted disphenoidal coordination geometry with O3 and N1 occupying the axial sites (O3–Bi1–N1 = 161.3(1)°), while O2 and N2 sit in equatorial positions (O2-Bi1-N2 = 94.7(1)°). The chelating κ^2^-*O*,*O* amido lactate moiety is bound to bismuth *via* a single covalent bond (Bi1–O2 = 2.111(3) Å) and a longer non-covalent interaction (Bi1–O3 = 2.590(3) Å). The C30–O2 and C31–O3 distances (1.401(6), 1.240(6) Å, respectively) are consistent with relatively elongated single and double bonds, respectively, compared to previously reported metal lactate compounds (M = Zn, Al, In, Sn, and Bi) (C–O = 1.35(2)–1.41(2) Å and C=O = 1.20(2)–1.24(3) Å).^11,15,17,21,23,62^

Poly(ε-caprolactone) is well established as a biocompatible polymer,^10^ whilst poly(γ-butyrolactone) is readily depolymerised back to γ-BL (chemically recyclable).^65,67^ Ring opening of γ-BL is challenging due to a lack of ring strain and only undergoes ROP under certain conditions.^63–67^ Noting the absence of structurally characterised ring-opened lactone complexes in the literature, the Bi–N insertion chemistry of 1 with ε-CL and γ-BL was investigated.

An equimolar reaction between ε-CL and 1 in benzene-*d*_6_ at room temperature led to the rapid formation of a new *C*_s_-symmetric complex, 3 (Scheme 1a), identified by two singlets at *δ* 0.60 and 0.28 ppm corresponding to the Si*Me*_2_ protons in the *in situ*^1^H NMR spectrum (Fig. S12). The presence of two N*Me*_2_ proton resonances at *δ* 2.65 and 2.24 ppm and five distinct C*H*_*2*_ proton environments indicated ring-opening of the ε-CL to form an amide derivative, the carbonyl carbon resonance of which is observed at *δ* 171.8 ppm (Fig. S14), slightly shifted to lower frequency with respect to ε-CL (δ 174.9 ppm). Although attempts to crystallise 3 proved futile, the ^1^H and ^13^C NMR data are fully consistent with insertion of a single molecule of ε-CL into the Bi–N bond to form [(NON^Dipp^)Bi(OC_5_H_10_C(O)NMe_2_)]. The formation of a coordination complex comprising a κ^2^-*O*,*O*′-chelated ring-opened ε-CL moiety has been predicted by DFT modelling of lactone ring-opening pathways,^28,68^ but the isolation of such a species is, to our knowledge, unprecedented.

The reaction of 1 with one equivalent of γ-BL proceeded at 50 °C, resulting in selective formation of the ring-opened compound [(NON^Dipp^)Bi(κ^1^-OC_3_H_6_C(O)NMe_2_)] (4) after 16 hours (Scheme 1a). The corresponding ^1^H NMR spectrum (Fig. S18) is very similar to that of 3: the NMe_2_ protons appear as two singlets at *δ* 2.47 and 2.15 ppm in the ^1^H NMR spectrum, whilst the CH_2_ protons of the ring-opened γ-BL moiety are located as two triplets at *δ* 4.30 and 1.82 ppm and a mutually coupled multiplet at *δ* 1.62 ppm. The carbonyl carbon signal was observed at *δ* 171.9 ppm in the ^13^C{^1^H} NMR spectrum (Fig. S20). X-ray diffraction analysis of colourless block-shaped single crystals confirmed the structure of 4 as an ω-amidoalkoxy bismuth complex. One bismuth complex is present per asymmetric unit, and the entire molecule is disordered across two components (occupancy 0.55 : 0.45) due to the co-crystalline occurrence of two types of intermolecular interactions (Fig. S65–S69). Although this disorder prevents detailed discussion of distances and angles, the structure solution (Scheme 1b) provides unambiguous confirmation of the atomic connectivity. The trigonal pyramidal bismuth centre is bound to the alkoxy oxygen atom of the ring-opened monomer, whilst the carbonyl oxygen of the terminal amide interacts with the bismuth centre of a neighbouring molecule. In the major component, the orientation of the carbonyl oxygen leads to a short (2.797(5) Å) intermolecular Bi1A–O3A contact as part of a macrocyclic, centrosymmetric dimer. In the minor component, an alternative conformation of the ring-opened lactone chain leads to propagation of a 1D coordination polymer *via* long (3.339(9) Å) Bi1B–O3B interactions (Fig. S65–S69). Compound 4 represents the first structurally characterised example of a metal complex derived from the ring-opening and insertion of a single γ-BL molecule.

As expected for the low ceiling temperature of poly(γ-BL),^63–67^ polymerisation was not observed when further equivalents of γ-BL were added to 1. By contrast, compound 1 was active in the ROP of l-LA and ε-CL (Scheme 2). No changes to the ^1^H NMR spectrum were observed at room temperature upon sequential addition of one to 50 equivalents of l-LA to 2 (Fig. S38–S40). After heating an equimolar mixture of 2 and l-LA at 50 °C for 2 hours, a small amount of PLLA oligomer was observed (Fig. S34–S37). Monitoring a freshly-prepared benzene-*d*_6_ solution of 1 and l-LA ([l-LA]_0_/[Bi]_0_ = 50; [l-LA]_0_ = 0.5 M) by *in situ*^1^H NMR spectroscopy (Fig. S41–S46) at 50 °C, however, revealed consumption of 1 within 10 minutes of mixing (l-LA conversion < 3%) and the formation of compound 2, as confirmed by comparison to the ^1^H NMR spectrum of 2 at the same temperature in the absence of l-LA (Fig. S9–S11). In addition to 2 as the major species, two quartets at *δ* 5.62, 4.78 ppm in the ^1^H NMR spectrum (Fig. S44) were assigned to the respective terminal methine OC(*H*)MeC(O)NMe_2_ and BiOC(*H*)MeCO_2_ environments of a propagating α-NMe_2_, ω-Bi PLLA chain. These resonances persist after subsequent consumption of 2 (Fig. S45); after 4.7 hours, the *in situ*^1^H NMR spectrum is consistent with an α-NMe_2_, ω-Bi PLLA chain, the NMe_2_ protons of which resonate as broad singlets at *δ* 2.48 and 2.16 ppm (Fig. S46). When an isolated sample of 2 was employed as an initiator under otherwise identical conditions, the initial ^1^H NMR spectrum (l-LA conversion <3%, Fig. S50) was similarly consistent with 2 as the major species and the subsequent evolution of the same set of resonances assigned to the propagating α-NMe_2_, ω-Bi PLLA chain (Fig. S47–S50). In neither case was an initiation period observed (Fig. S51). These data suggest that compound 2 is rapidly formed as an insertion-transesterification product when 1 is employed as initiator, and that 2 itself is active for subsequent propagation.

**Scheme 2:**
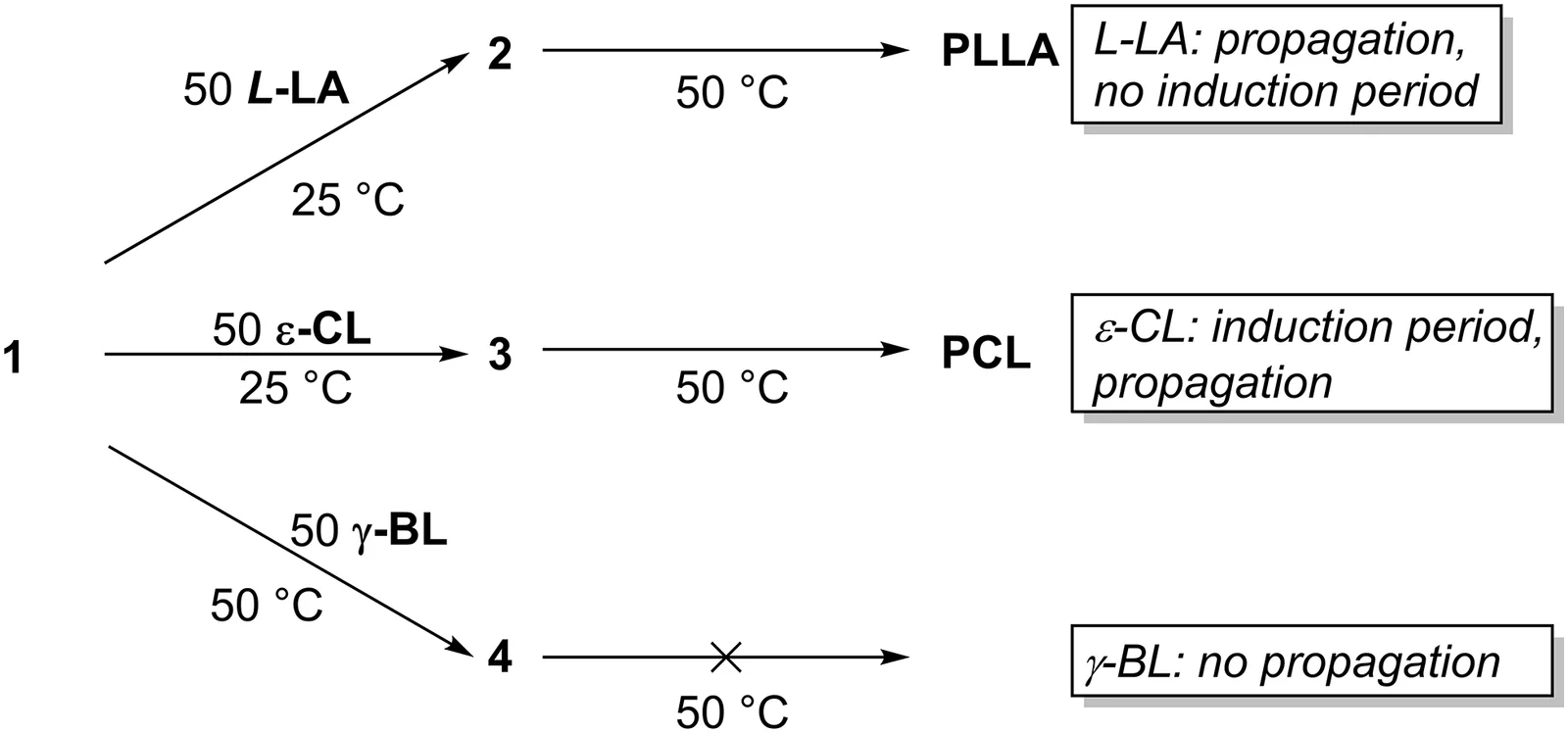
Summary of the reaction outcomes when 1 is reacted with excess (50 equivalents) monomer.

A linear plot of ln([l-LA]_0_/[l-LA]_*t*_) *vs.* polymerisation time is consistent with first-order dependence on monomer concentration for both 1 and 2, with similar activities (*k*_obs_ = 0.219 ± 0.008 and 0.191 ± 0.007 h^−1^, respectively, Fig. S51). Good agreement between *M*_*n*,SEC_ and *M*_*n*,theo_ and moderate polydispersity (*Đ*_M_ = 1.36, 1.20, respectively) was observed in both cases (Tables S3, S4 and Fig. S32, S52). Analysis of the isolated polymer by MALDI-ToF resulted in a mass-spectrum with a series of peaks separated by *m*/*z* = 72, indicative of intermolecular transesterification (Fig. S53). The major series of peaks was assigned to the PLLA chain terminated by α-NMe_2_ and ω-OH end groups, consistent with a coordination-insertion mechanism. An additional series of peaks at the lower molecular weight range of the MALDI-ToF spectrum was attributed to macrocyclic PLLA formed *via* intramolecular transesterification. The α-NMe_2_ end group was also observed as two characteristic proton singlets located at *δ* 2.45 and 2.09 ppm in the ^1^H NMR spectrum of the isolated polymer (Fig. S54). Consistent with a higher energy monomer,^69^ a higher rate (*k*_obs_ = 0.738 ± 0.005 h^−1^) was observed for the ROP of *meso*-LA initiated by 1 compared to that of *rac*-LA, d-LA, and l-LA (*k*_obs_ = 0.531 ± 0.041 h^−1^, 0.365 ± 0.022 h^−1^, 0.219 ± 0.008 h^−1^, respectively; Table S1). Stereochemical effects might be responsible for the relatively higher rate of *rac*-LA *cf.*l-LA, whilst the difference in rates for d- and l-LA remains unaccounted for. Lower than expected molecular weights for *meso*-LA and *rac*-LA (Table S1) suggests susceptibility of *meso*- and *rac*-linkages towards transesterification. A bimodal molecular weight distribution was obtained for *rac*-LA (*Đ*_M_ = 2.07; Fig. S26), possibly due to the presence of different active species arising from transesterification and aggregation.

ROP of ε-CL (50 °C) initiated by 1 displayed an induction period of 2 h and lower subsequent activity (*k*_obs_ = 0.061 ± 0.003 h^−1^*cf.* 0.219 ± 0.008 h^−1^ for l-LA; first order based on ln([ε-CL]_0_/[ε-CL]_*t*_); [monomer]_0_ : [Bi]_0_ = 50 : 1; Fig. S58). The ^1^H NMR spectrum obtained immediately after addition of ε-CL (Fig. S56 and S57) indicated the rapid formation of 3, suggesting that the induction period is due to low activity of bismuth alkoxide 3 towards ring-opening of further monomers. Monomer conversion of 66% was achieved after 15 h. at 50 °C, and the resulting *in situ*^1^H NMR spectrum was consistent with α-NMe_2_, ω-BiO polymer (Fig. S56 and S57). ^1^H NMR spectroscopy of the isolated polymer was consistent with α-NMe_2_, ω-OH chain ends (Fig. S60). Moderate polydispersity (*Đ*_M_ = 1.32) and lower than expected polymer molecular weight (*M*_*n*,SEC_ = 2900 g mol^−1^*vs. M*_*n*,theo_ = 3800 g mol^−1^; Fig. S59) are consistent with the occurrence of transesterification.

In summary, Bi–N bonds can mediate clean first-insertion events across lactide and lactone substrates, but with distinct monomer-dependent outcomes following the initial cyclic-ester ring opening. The ring-opened l-LA derivative undergoes subsequent transesterification to provide amidolactate 2 before propagation to yield PLA; ε-CL gives an initially dormant ring-opened species 3 before delayed propagation, and γ-BL affords a trapped insertion product 4 that does not propagate under the conditions examined.

## Conflicts of interest

There are no conflicts to declare.

## Supplementary Material

CC-062-D6CC05601F-s001

CC-062-D6CC05601F-s002

## Data Availability

The data supporting this article have been included as part of the supplementary information (SI). Supplementary information: synthetic protocols, characterisation of compounds 1–4, polymerisation data. See DOI: https://doi.org/10.1039/d6cc05601f. CCDC 2580882–2580884 contain the supplementary crystallographic data for this paper.^70^

## References

[cit1] European Bioplastics, https://www.european-bioplastics.org/, (accessed March 2026)

[cit2] Auras R., Harte B., Selke S. (2004). Macromol. Biosci..

[cit3] Hoogsteen W., Postema A. R., Pennings A. J., Ten Brinke G., Zugenmaier P. (1990). Macromolecules.

[cit4] Perepelkin K. E. (2002). Fibre Chem..

[cit5] Manavitehrani I., Fathi A., Wang Y., Maitz P. K., Dehghani F. (2015). ACS Appl. Mater. Interfaces.

[cit6] Kulkarni R. K., Pani K. C., Neuman C., Leonard F. (1966). Arch. Surg..

[cit7] Ashammakhi N., Rokkanen P. (1997). Biomaterials.

[cit8] Zhang Y., Wu X., Han Y., Mo F., Duan Y., Li S. (2010). Int. J. Pharm..

[cit9] Lasprilla A. J. R., Martinez G. A. R., Lunelli B. H., Jardini A. L., Filho R. M. (2012). Biotechnol. Adv..

[cit10] Labet M., Thielemans W. (2009). Chem. Soc. Rev..

[cit11] Turner Z. R., Wilmore J. T., Rees N. H., Buffet J.-C. (2022). Dalton Trans..

[cit12] Buchard A., Davidson M. G., Gobius du Sart G., Jones M. D., Kociok-Köhn G., McCormick S. N., McKeown P. (2023). Inorg. Chem..

[cit13] Ovitt T. M., Coates G. W. (2002). J. Am. Chem. Soc..

[cit14] Lewiński J., Horeglad P., Wójcik K., Justyniak I. (2005). Organometallics.

[cit15] Yuntawattana N., McGuire T. M., Durr C. B., Buchard A., Williams C. K. (2020). Catal. Sci. Technol..

[cit16] Tang Z., Pang X., Sun J., Du H., Chen X., Wang X., Jing X. (2006). J. Polym. Sci., Part A: Polym. Chem..

[cit17] Nomura N., Ishii R., Yamamoto Y., Kondo T. (2007). Chem. – Eur. J..

[cit18] Ko B.-T., Wang F.-C., Sun Y.-L., Lin C.-H., Lin C.-C., Kuo C.-Y. (1998). Polyhedron.

[cit19] Nomura N., Ishii R., Akakura M., Aoi K. (2002). J. Am. Chem. Soc..

[cit20] Klitzke J. S., Roisnel T., Kirillov E., Casagrande Jr. O. D. L., Carpentier J.-F. (2014). Organometallics.

[cit21] Chamberlain B. M., Cheng M., Moore D. R., Ovitt T. M., Lobkovsky E. B., Coates G. W. (2001). J. Am. Chem. Soc..

[cit22] Horeglad P., Kruk P., Pécaut J. (2010). Organometallics.

[cit23] Wang L., Kefalidis C. E., Sinbandhit S., Dorcet V., Carpentier J.-F., Maron L., Sarazin Y. (2013). Chem. – Eur. J..

[cit24] Dove A. P., Gibson V. C., Marshall E. L., Rzepa H. S., White A. J. P., Williams D. J. (2006). J. Am. Chem. Soc..

[cit25] Steudel A., Stehr J., Siebel E., Fischer R. D. (1998). J. Organomet. Chem..

[cit26] Hsiao M.-W., Lin C.-C. (2013). Dalton Trans..

[cit27] Horeglad P., Cybularczyk M., Litwińska A., Dąbrowska A. M., Dranka M., Żukowska G. Z., Urbańczyk M., Michalak M. (2016). Polym. Chem..

[cit28] Nifant'ev I. E., Shlyakhtin A. V., Bagrov V. V., Minyaev M. E., Churakov A. V., Karchevsky S. G., Birin K. P., Ivchenko P. V. (2017). Dalton Trans..

[cit29] Dagorne S., Le Bideau F., Welter R., Bellemin-Laponnaz S., Maisse-François A. (2007). Chem. – Eur. J..

[cit30] Luke A. M., Peterson A., Chiniforoush S., Mandal M., Popowski Y., Sajjad H., Bouchey C. J., Shopov D. Y., Graziano B. J., Yao L. J., Cramer C. J., Reineke T. M., Tolman W. B. (2020). Macromolecules.

[cit31] Kazarina O. V., Gourlaouen C., Karmazin L., Morozov A. G., Fedushkin I. L., Dagorne S. (2018). Dalton Trans..

[cit32] Evans W. J., Shreeve J. L., Doedens R. J. (1993). Inorg. Chem..

[cit33] Evans W. J., Shreeve J. L., Ziller J. W., Doedens R. J. (1995). Inorg. Chem..

[cit34] Lewiński J., Horeglad P., Tratkiewicz E., Grzenda W., Lipkowski J., Kolodziejczyk E. (2004). Macromol. Rapid Commun..

[cit35] Afroz T., Seo D. M., Han S.-D., Boyle P. D., Henderson W. A. (2015). J. Phys. Chem. C.

[cit36] Saura-Llamas I., Dalton D. M., Arif A. M., Gladysz J. A. (1992). Organometallics.

[cit37] de Marcillac P., Coron N., Dambier G., Leblanc J., Moalic J.-P. (2003). Nature.

[cit38] Briand G. G., Burford N. (1999). Chem. Rev..

[cit39] Tanzi M. C., Verderio P., Lampugnani M. G., Resnati M., Dejana E., Sturani E. (1994). J. Mater. Sci.: Mater. Med..

[cit40] Yamada T., Jung D.-Y., Sawada R., Tsuchiya T. (2008). J. Biomed. Mater. Res., Part B.

[cit41] Dantas F. J. S., de Mattos J. C. P., Moraes M. O., Viana M. E., Lage C. A. S., Cabral-Neto J. B., Leitão A. C., Bernardo-Filho M., Bezerra R. J. A. C., Carvalho J. J., Caldeira-de-Araújo A. (2002). Food Chem. Toxicol..

[cit42] Gagnon A., Dansereau J., Le Roch A. (2017). Synthesis.

[cit43] Hannah T. J., McCarvell W. M., Kirsch T., Bedard J., Hynes T., Mayho J., Bamford K. L., Vos C. W., Kozak C. M., George T., Masuda J. D., Chitnis S. S. (2023). Chem. Sci..

[cit44] Bonné C., Pahwa A., Picard C., Visseaux M. (2017). Inorg. Chim. Acta.

[cit45] Ando F., Hayashi T., Ohashi K., Koketsu J. (1975). J. Inorg. Nucl. Chem..

[cit46] Ando F., Kohmura Y., Koketsu J. (1987). Bull. Chem. Soc. Jpn..

[cit47] Nekoueshahraki B., Pillai Sarish S., Roesky H. W., Stern D., Schulzke C., Stalke D. (2009). Angew. Chem., Int. Ed..

[cit48] Nekoueshahraki B., Samuel P. P., Roesky H. W., Stern D., Matussek J., Stalke D. (2012). Organometallics.

[cit49] Dengel H., Lichtenberg C. (2016). Chem. – Eur. J..

[cit50] Ramler J., Poater J., Hirsch F., Ritschel B., Fischer I., Bickelhaupt F. M., Lichtenberg C. (2019). Chem. Sci..

[cit51] Oberdorf K., Grener P., Pfister P., Hanft A., Rempel A., Lichtenberg C. (2023). ChemPlusChem.

[cit52] Oberdorf K., Hanft A., Xie X., Bickelhaupt F. M., Poater J., Lichtenberg C. (2023). Chem. Sci..

[cit53] Ritschel B., Poater J., Dengel H., Bickelhaupt F. M., Lichtenberg C. (2018). Angew. Chem., Int. Ed..

[cit54] Oberdorf K., Grenzer P., Wieprecht N., Ramler J., Hanft A., Rempel A., Stoy A., Radacki K., Lichtenberg C. (2021). Inorg. Chem..

[cit55] Oberdorf K., Hanft A., Ramler J., Krummenacher I., Bickelhaupt F. M., Poater J., Lichtenberg C. (2021). Angew. Chem., Int. Ed..

[cit56] Schwamm R. J., Lein M., Coles M. P., Fitchett C. M. (2018). Chem. Commun..

[cit57] Schwamm R. J., Lein M., Coles M. P., Fitchett C. M. (2017). J. Am. Chem. Soc..

[cit58] Schwamm R. J., Lein M., Coles M. P., Fitchett C. M. (2016). Angew. Chem., Int. Ed..

[cit59] Schwamm R. J., Harmer J. R., Lein M., Fitchett C. M., Granville S., Coles M. P. (2015). Angew. Chem., Int. Ed..

[cit60] Clegg W., Compton N. A., Errington R. J., Fisher G. A., Green M. E., Hockless D. C. R., Norman N. C. (1991). Inorg. Chem..

[cit61] Chisholm M. H., Delbridge E. E. (2003). New J. Chem..

[cit62] Klitzke J. S., Roisnel T., Kirillov E., Casagrande Jr. O. D. L., Carpentier J.-F. (2014). Organometallics.

[cit63] Houk K. N., Jabbari A., Hall H. K., Alemán C. (2008). J. Org. Chem..

[cit64] Hong M., Chen E. Y.-X. (2016). Angew. Chem., Int. Ed..

[cit65] Hong M., Chen E. Y. X. (2016). Nat. Chem..

[cit66] Zhao N., Ren C., Li H., Li Y., Liu S., Li Z. (2017). Angew. Chem., Int. Ed..

[cit67] Zhu J.-B., Watson E. M., Tang J., Chen E. Y.-X. (2018). Science.

[cit68] Miranda M. O., DePorre Y., Vazquez-Lima H., Johnson M. A., Marell D. J., Cramer C. J., Tolman W. B. (2013). Inorg. Chem..

[cit69] Buffet J.-C., Okuda J. (2011). Polym. Chem..

[cit70] (a) CCDC 2580882: Experimental Crystal Structure Determination, 2026 10.5517/ccdc.csd.cc2smm8b

