# Supplementary material for: Cyclic ester insertion into a bismuth–amide bond: isolable molecular models for ROP initiation
Source: Chem Commun (Camb). 2026 Sep 21. Online ahead of print. doi: 10.1039/d6cc05601f (PMC13659864; doi:10.1039/d6cc05601f)
Supplement: Supplementary file 1 [file CC-OLF-D6CC05601F-s002.pdf]

This journal is © The Royal Society of Chemistry 2026

Cyclic ester insertion into a bismuth–amide bond: isolable molecular models for ROP initiation

Gabija Navickaite, Louis J. Morris, Alexander Evans, Zoë R. Turner* and Dermot O′Hare*

Chemistry Research Laboratory, Department of Chemistry, University of Oxford, 12 Mansfield Road, Oxford, OX1 3TA, UK; zoe.turner@earth.ox.ac.uk; dermot.ohare@chem.ox.ac.uk

Supporting information

Table of Contents

1. General considerations ... 1
2. Synthesis and characterization of compounds **1 – 4** ... 3
2.1. [(NON^{Dipp})Bi(NMe$_2$)] (**1**) ... 3
2.2. [(NON^{Dipp})Bi(OCH(Me)C(O)NMe$_2$)] (**2**) ... 5
2.3. [(NON^{Dipp})Bi(OC$_5$H$_{10}$C(O)NMe$_2$)] (**3**) ... 10
2.4. [(NON^{Dipp})Bi(OC$_3$H$_6$C(O)NMe$_2$)] (**4**) ... 13
3. Polymerisation studies ... 17
3.1. General procedure for polymerisation ... 17
3.2. Monomer stereochemistry variation ... 17
3.3. Initiator concentration dependence ... 20
3.4. Temperature variation ... 22
3.5. Addition of 1 equiv. *L*-LA to compound **2** ... 24
3.6. Sequential addition of increasing equivalents of *L*-LA to **1** ... 26
3.7. In situ NMR monitoring for the reaction of **1** with *L*-LA ... 28
3.8. Lactide polymerisation studies using bismuth amidolactate **2** ... 34
3.9. End-group analysis of PLLA ... 39
3.10. ε-Caprolactone polymerisation using bismuth amide **1** ... 41
4. Crystallographic data ... 45
4.1. General considerations, crystal data and refinement ... 45
4.2. Crystal structure determination of [(NON^{dipp})Bi(NMe$_2$)] (**1**) ... 47
4.3. Crystal structure determination of [(NON^{dipp})Bi(κ^2-*O*,*O*-OC(CH$_3$)HC(O)NMe$_2$)] (**2**) 48
4.4. Crystal structure determination of [(NON^{dipp})Bi(O(CH$_2$)$_3$C(O)NMe$_2$)] (**4**) ... 49
5. References ... 52

1. General considerations

Air- and moisture-sensitive manipulations were conducted under an inert atmosphere of nitrogen, employing standard Schlenk line techniques and an MBraun Unilab glove box.

Reactions out using bismuth reagents were carried out whilst avoiding exposure to light, but we have found that solutions and solid samples of the (NON^{dipp})-bismuth complexes described in this work can be handled breifly in ambient lab-light without appreciable decomposition. Glassware was oven-dried at temperatures greater than 150 °C overnight prior to use. Pentane, n-hexane, toluene, and benzene were dried using an MBraun SPS-800 solvent purification system, stored over a potassium mirror, and degassed under partial pressure prior to use. Tetrahydrofuran was distilled from sodium metal/benzophenone, stored over activated 3 Å molecular sieves and degassed under partial pressure prior to use. Diethyl ether was distilled from sodium metal/benzophenone, stored over a potassium mirror, and degassed under partial pressure prior to use. Benzene-d_6 was dried over potassium, distilled under reduced pressure, and freeze−pump−thaw degassed three times prior to use. *L*-Lactide (Corbion Biomaterials) was recrystallized repeatedly from dry toluene and dried under vacuum. β-Butyrolactone and ε-caprolactone were dried over calcium hydride and distilled. $Bi(NMe_2)_3$[1] and (NON^{Dipp})H_2[2] were prepared according to the literature procedures.

NMR spectra were recorded on a Bruker Avance III HD NanoBay NMR (9.4 T, 400.2 MHz), a Bruker Avance III NMR (11.75 T, 499.9 MHz), a Bruker Avance NMR fitted with a ^{13}C cryoprobe (11.75 T, 500.3 MHz) or a Bruker NEO 600 (14.1 T, 600.4 MHz) spectrometers. Spectra were recorded at 298 K, unless otherwise stated, and internally referenced to residual protio solvent ^{1}H or ^{13}C resonances. MestreNova software was used to process and analyze spectra. Chemical shifts were quoted in δ (ppm) with respect to tetramethylsilane (δ = 0 ppm), and coupling constants were reported in Hertz. Air sensitive samples were prepared in a glove box under a nitrogen atmosphere using dried deuterated solvents in 5 mm J. Young's tap NMR spectroscopy tubes.

Samples for IR spectroscopy were prepared in a glove box under a nitrogen atmosphere as pellets pressed with anhydrous potassium bromide, which was dried above 150 °C at 10^{-6} mbar for 24 hours prior to use. FTIR spectra were measured using a Nicolet iS5 ThermoScientific spectrometer, with a background spectrum run prior to the sample and subtracted from the sample spectra.

Elemental analyses were carried out in duplicate by Ms Orla McCullough at London Metropolitan University.

MALDI-ToF MS analysis was performed by Dr Victor Mikhailov or Mr Justin Hayden (University of Oxford) using a Bruker MALDI Autoflex ToF MS. Polymer samples were prepared in chloroform at a concentration of 1.0 mg mL^{-1} and filtered through a 0.2 μm PTFE filter membrane before analysis. The DHB matrix was prepared by mixing the sample with DHB (10 mg mL^{-1} in 70:30 H_2O:CH_3CN) in a 1:1 volume ratio. The DCTB matrix was prepared by mixing 10 μL of a sample with 10 μL DCTB (40 mg mL^{-1} in THF) and 2.5 μL KTFA (5 mg mL^{-1} in THF). 1.5 μL of the mixed solutions were spotted on the MALDI plate and dried.

Polymer molecular weights and molecular weight distributions were recorded on a Polymer Laboratories PLSEC50 Plus instrument equipped with a Polymer Laboratories PLgel Mixed-D column (300 mm length, 7.5 mm diameter) and a refractive index (RI) detector. THF (Sigma Aldrich, HPLC grade) was used as the eluent flowing at 1.0 mL min^{-1} at 30 °C. Polymer samples were prepared in THF at a concentration of 1.0 mg mL^{-1} and filtered through a 0.2 μm PTFE filter membrane before analysis. Experimental molecular weights were recorded as linear polystyrene equivalents and corrected by Mark-Houwink correction factors for PLA

(0.58) and PCL (0.56).[3, 4] Theoretical M_n was calculated for PLA = (conv.(%) × [L-LA]$_0$/[Bi]$_0$) × 144.13 + (M_n of end groups); and for PCL = (conv.(%) × [ε-CL]$_0$/[Bi]$_0$) × 114.14 + (M_n of end groups).

2. Synthesis and characterization of compounds 1 – 4

2.1. [(NON^{Dipp})Bi(NMe$_2$)] (1)

(NON^{Dipp})H$_2$ (112 mg, 0.231 mmol) in benzene (1 mL) was added dropwise to a solution of freshly sublimed Bi(NMe$_2$)$_3$ (78.8 mg, 0.231 mmol) in benzene (1 mL). The resulting yellow mixture was allowed to react overnight and freeze-dried to produce [(NON^{Dipp})Bi(NMe$_2$)] (**1**) as a pale-yellow solid. Yield: 127 mg (75%). Colourless block-shaped single crystals suitable for X-ray diffraction analysis were obtained from a saturated *n*-hexane solution at room temperature.

^{1}H NMR (benzene-d_6, 400 MHz): δ 7.19 (dd, $^3J_{H–H}$ = 7.6 Hz, $^4J_{H–H}$ = 1.8 Hz, 2H, *m*-C$_6$*H*$_3$), 7.13 (dd, $^3J_{H–H}$ = 7.7 Hz, $^4J_{H–H}$ = 1.8 Hz, 2H, *m*-C$_6$*H*$_3$), 7.04 (t, $^3J_{H–H}$ = 7.6 Hz, 2H, *p*-C$_6$*H*$_3$), 3.89 (sept, $^3J_{H–H}$ = 6.8 Hz, 4H, C*H*Me$_2$), 3.78 (s, 6H, N*Me*$_2$), 1.35 (d, $^3J_{H–H}$ = 7.1 Hz, 6H, CH*Me*$_2$), 1.33 (d, $^3J_{H–H}$ = 7.1 Hz, 6H, CH*Me*$_2$), 1.22 (d, $^3J_{H–H}$ = 6.8 Hz, 6H, CH*Me*$_2$), 1.19 (d, $^3J_{H–H}$ = 6.9 Hz, 6H, CH*Me*$_2$), 0.44 (s, 6H, Si*Me*$_2$), 0.27 (s, 6H, Si*Me*$_2$) ppm.

^{13}C{^{1}H} NMR (benzene-d_6, 101 MHz): δ 150.6 (*o*-*C*$_6$H$_3$), 148.3 (*o*-*C*$_6$H$_3$), 140.1 (*i*-*C*$_6$H$_3$), 125.8 (*p*-*C*$_6$H$_3$), 124.6 (*m*-*C*$_6$H$_3$), 124.2 (*m*-*C*$_6$*H*$_3$), 45.7 (N*Me*$_2$), 28.7 (CH*Me*$_2$), 27.7 (*C*HMe$_2$), 27.5 (*C*HMe$_2$), 27.4 (CH*Me*$_2$), 25.3 (CH*Me*$_2$), 24.8 (CH*Me*$_2$), 3.9 (Si*Me*$_2$), 3.1 (Si*Me*$_2$) ppm.

IR (KBr): 2963, 1462, 1440, 1257, 1061, 910, 793 cm^{-1}.

Analysis calculated for C$_{30}$H$_{52}$BiN$_3$OSi$_2$: C, 48.96; H, 7.12; N, 5.71%. Found: C, 48.61; H, 7.00; N, 5.10%.

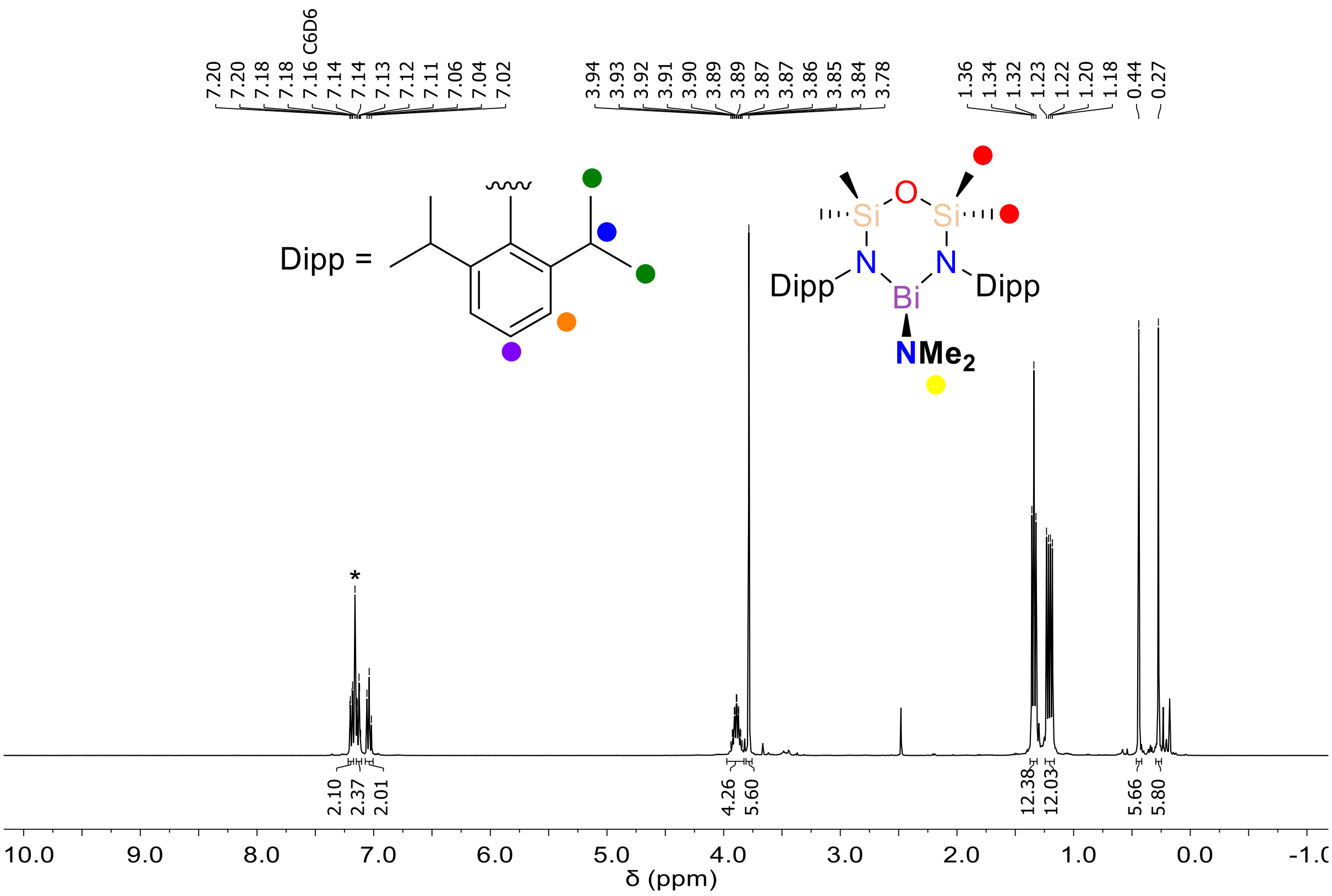

Figure S1: ^{1}H NMR spectrum (C_6D_6, 400 MHz) of **1**. *Denotes residual protio solvent fraction of C_6D_6.

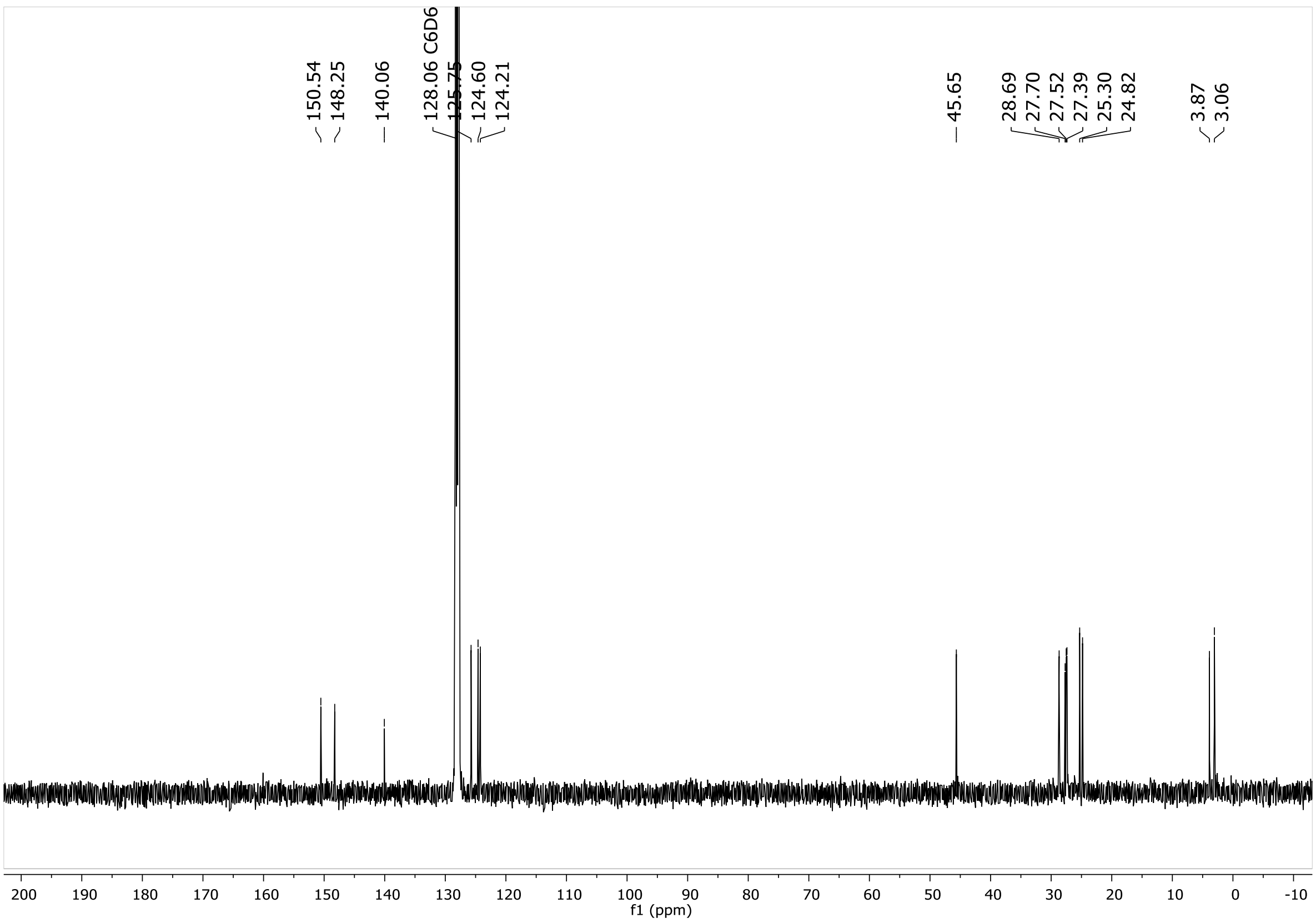

Figure S2: $^{13}C\{^{1}H\}$ NMR spectrum (101 MHz, benzene-d_6) of **1**.

2.2. [(NON^{Dipp})Bi(OCH(Me)C(O)NMe$_2$)] (2)

L-Lactide (7.0 mg, 0.0485 mmol) was added to a solution of [(NON^{Dipp})Bi(NMe$_2$)] (**1**, 72 mg, 0.0970 mmol) in benzene (1 mL), resulting in a pale-yellow reaction mixture. The solution was freeze-dried to afford **2** as a white solid. Yield: 32 mg (82%). Colourless block-shaped single crystals suitable for X-ray diffraction analysis were obtained from slow evaporation of a saturated *n*-hexane solution at room temperature.

^{1}H NMR (benzene-*d*$_6$, 600 MHz): δ 7.34 (dd, $^3J_{H-H}$ = 7.6 Hz, $^4J_{H-H}$ = 1.8 Hz, 1H, *m*-C$_6$*H*$_3$), 7.20 (dd, $^3J_{H-H}$ = 7.6 Hz, $^4J_{H-H}$ = 1.8 Hz, 1H, *m*-C$_6$*H*$_3$), 7.18 (overlapped with C$_6$D$_5$H, 1H, *m*-C$_6$*H*$_3$), 7.11 (dd, $^3J_{H-H}$ = 7.6 Hz, $^4J_{H-H}$ = 1.7 Hz, 1H, *m*-C$_6$*H*$_3$), 7.06 (t, $^3J_{H-H}$ = 7.6 Hz, 1H, *p*-C$_6$*H*$_3$), 6.94 (t, $^3J_{H-H}$ = 7.6 Hz, 1H, *p*-C$_6$*H*$_3$), 6.22 (q, $^3J_{H-H}$ = 6.6 Hz, 1H, OC*H*Me) 4.33 (sept, $^3J_{H-H}$ = 6.9 Hz, 1H, C*H*Me$_2$), 4.22 (sept, $^3J_{H-H}$ = 6.8 Hz, 1H, C*H*Me$_2$), 4.03 (sept, $^3J_{H-H}$ = 6.9 Hz, 1H, C*H*Me$_2$), 3.97 (sept, $^3J_{H-H}$ = 6.8 Hz, 1H, C*H*Me$_2$), 2.04 (s, 3H, N*Me*$_2$), 1.83 (s, 3H, N*Me*$_2$), 1.53 (d, $^3J_{H-H}$ = 6.7 Hz, 3H, CH*Me*$_2$, 1.42–1.37 (m, 9H, CH*Me*$_2$), 1.36–1.28 (m, 12H, CH*Me*$_2$), 0.78 (s, 3H, Si*Me*$_2$), 0.71 (d, $^3J_{H-H}$ = 6.6 Hz, 3H, OCH*Me*), 0.62 (s, 3H, Si*Me*$_2$), 0.44 (s, 3H, Si*Me*$_2$), 0.36 (s, 3H, Si*Me*$_2$) ppm.

^{13}C{^{1}H} NMR (benzene-*d*$_6$, 151 MHz): δ 182.6 (*C*O), 150.3 (*o*-*C*$_6$H$_3$), 150.0 (*o*-*C*$_6$H$_3$), 147.3 (*o*-*C*$_6$H$_3$), 146.7(*o*-*C*$_6$H$_3$), 142.3 (*i*-*C*$_6$H$_3$), 142.1 (*i*-*C*$_6$H$_3$), 124.6 (*p*-*C*$_6$H$_3$), 124.5 (*p*-*C*$_6$H$_3$), 124.2 (*m*-*C*$_6$H$_3$), 123.7 (*m*-*C*$_6$H$_3$), 123.5 (*m*-*C*$_6$H$_3$), 123.5 (*m*-*C*$_6$H$_3$), 67.1 (O*C*HMe), 35.7 (N*Me*$_2$), 35.4 (N*Me*$_2$), 27.9 (*C*HMe$_2$), 27.8 (*C*HMe$_2$), 27.6 (*C*HMe$_2$), 27.4 (*C*HMe$_2$), 27.3 (CH*Me*$_2$), 26.9 (CH*Me*$_2$), 26.3 (CH*Me*$_2$), 26.2 (CH*Me*$_2$), 26.1 (CH*Me*$_2$), 26.0 (CH*Me*$_2$), 26.0 (CH*Me*$_2$), 25.5 (CH*Me*$_2$), 23.5 (OCH*Me*), 4.4 (Si*Me*$_2$), 4.28 (Si*Me*$_2$), 3.7 (Si*Me*$_2$), 3.4 (Si*Me*$_2$) ppm.

IR (KBr): 2963, 1613 (C=O stretching), 1250, 1188, 936, 827 cm^{-1}.

Analysis calculated for C$_{33}$H$_{56}$BiN$_3$O$_3$Si$_2$: C, 49.06; H, 6.99; N, 5.20%. Found: C, 49.82; H, 6.88; N, 4.81%.

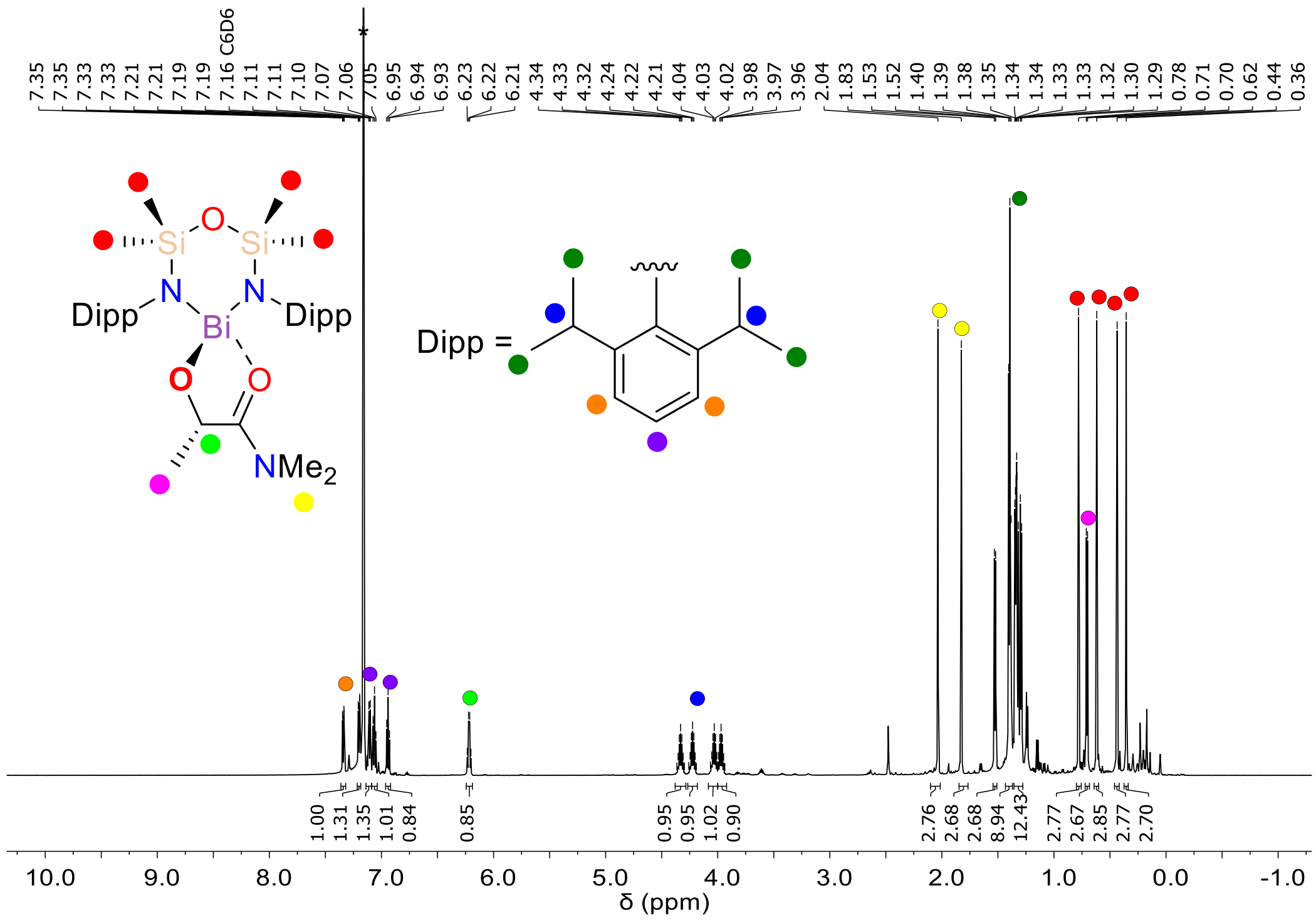

Figure S3: ^{1}H NMR spectrum (C$_6$D$_6$, 600 MHz) of **2**. *Denotes residual protio solvent fraction of C$_6$D$_6$.

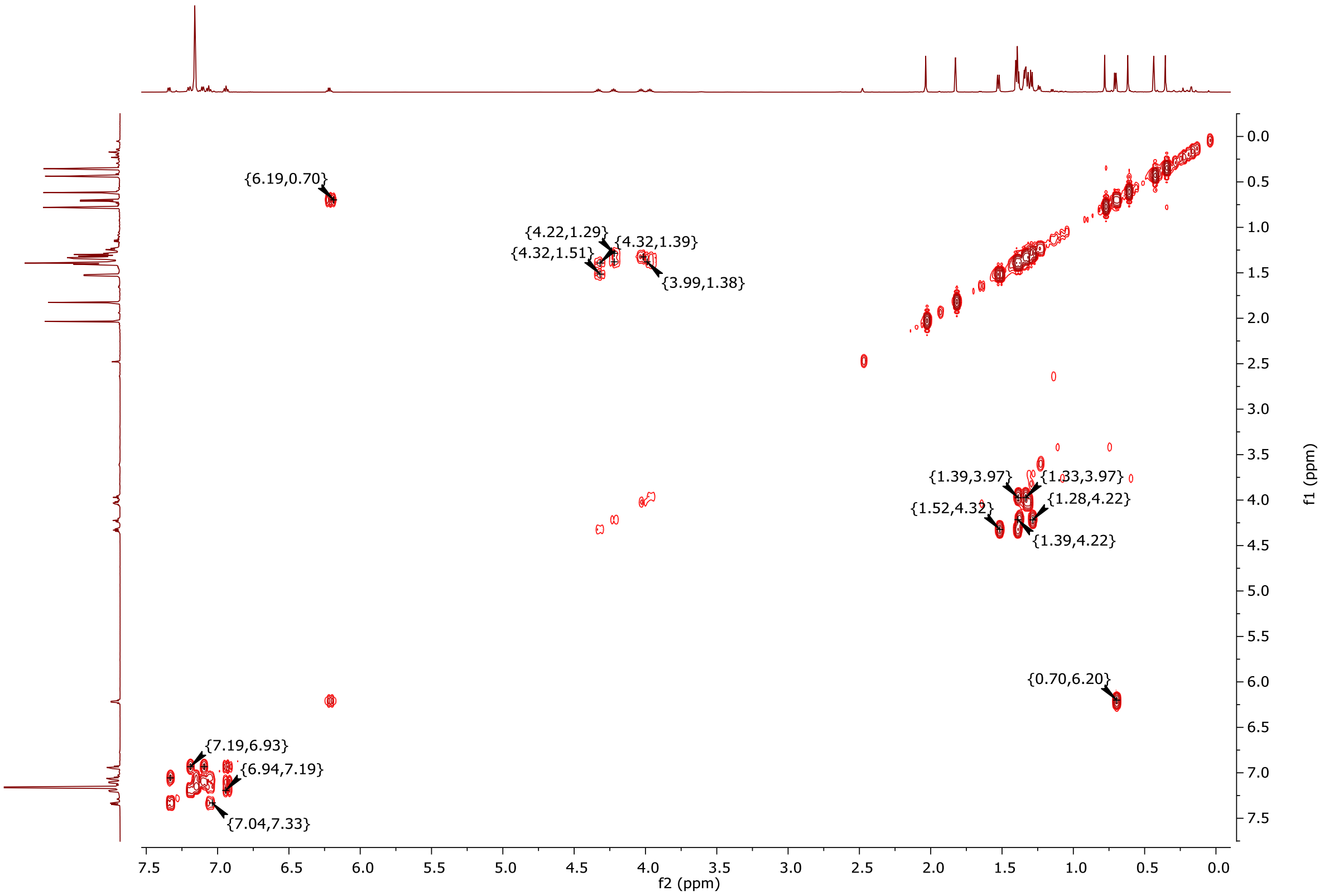

Figure S4: ^{1}H-^{1}H COSY NMR spectrum of **2** (C_6D_6).

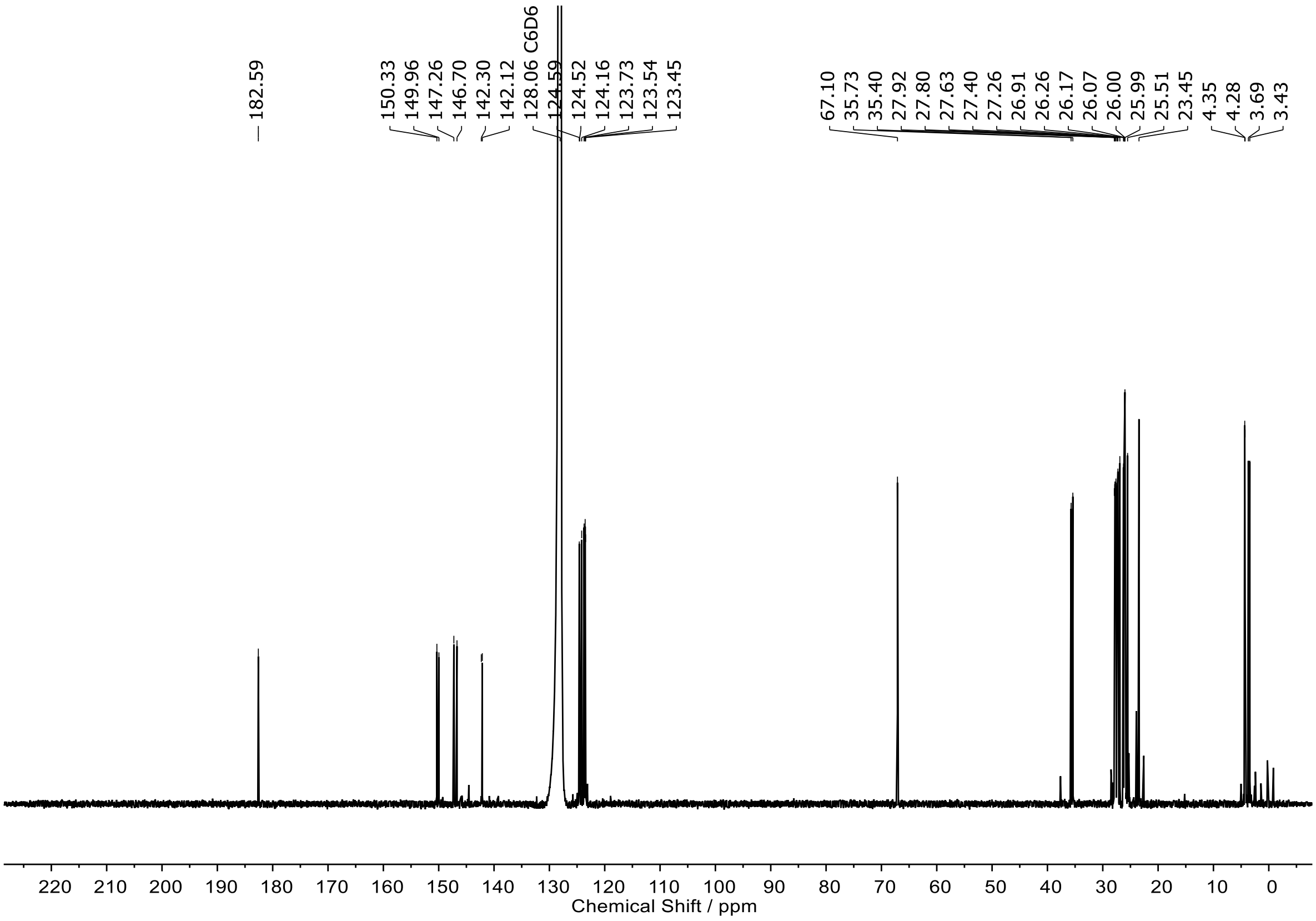

Figure S5: $^{13}C\{^1H\}$ NMR spectrum (151 MHz, benzene-d_6) of compound **2**.

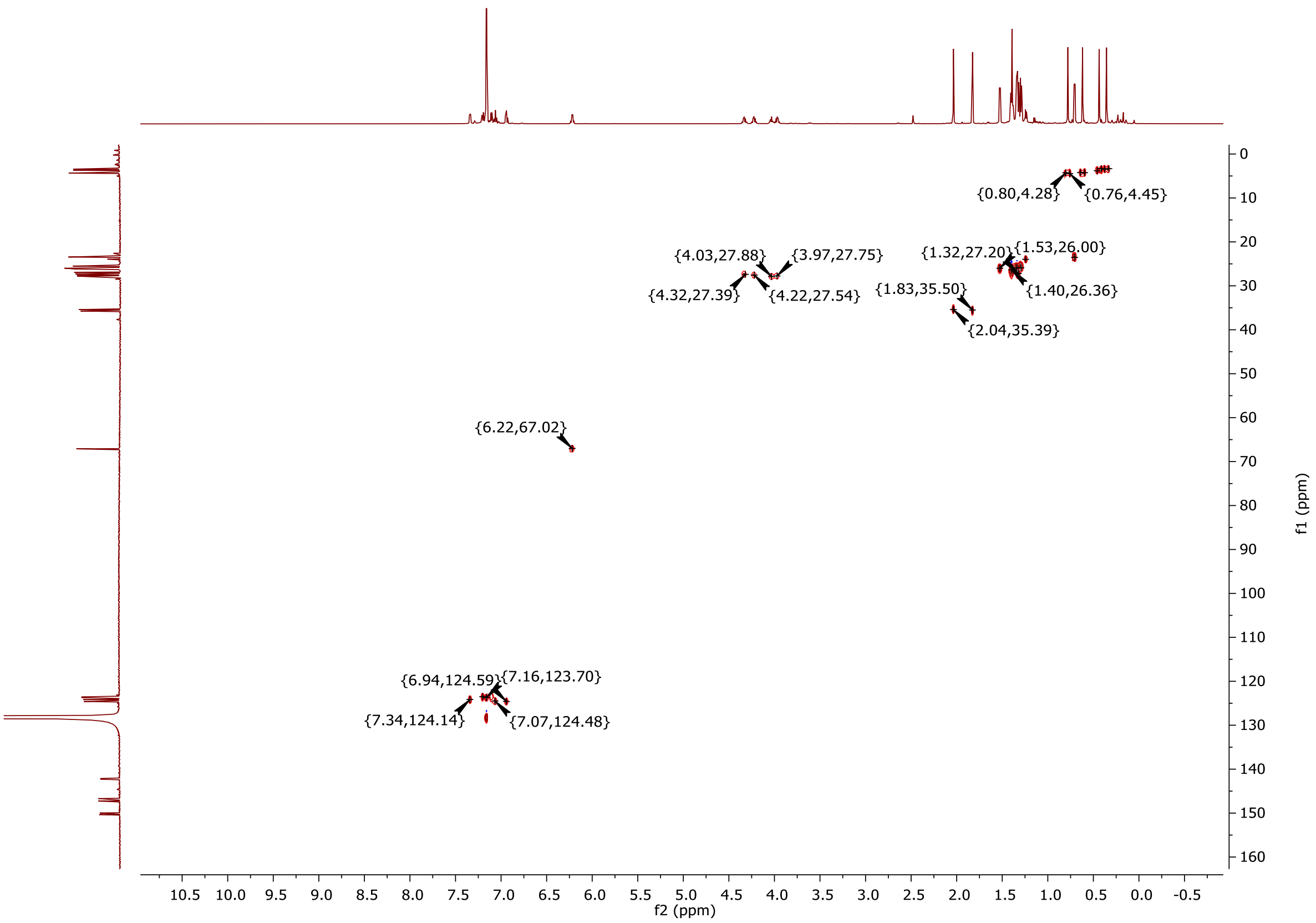

Figure S6: ^{13}C-^{1}H HSQC NMR spectrum of **2** (benzene-d_6).

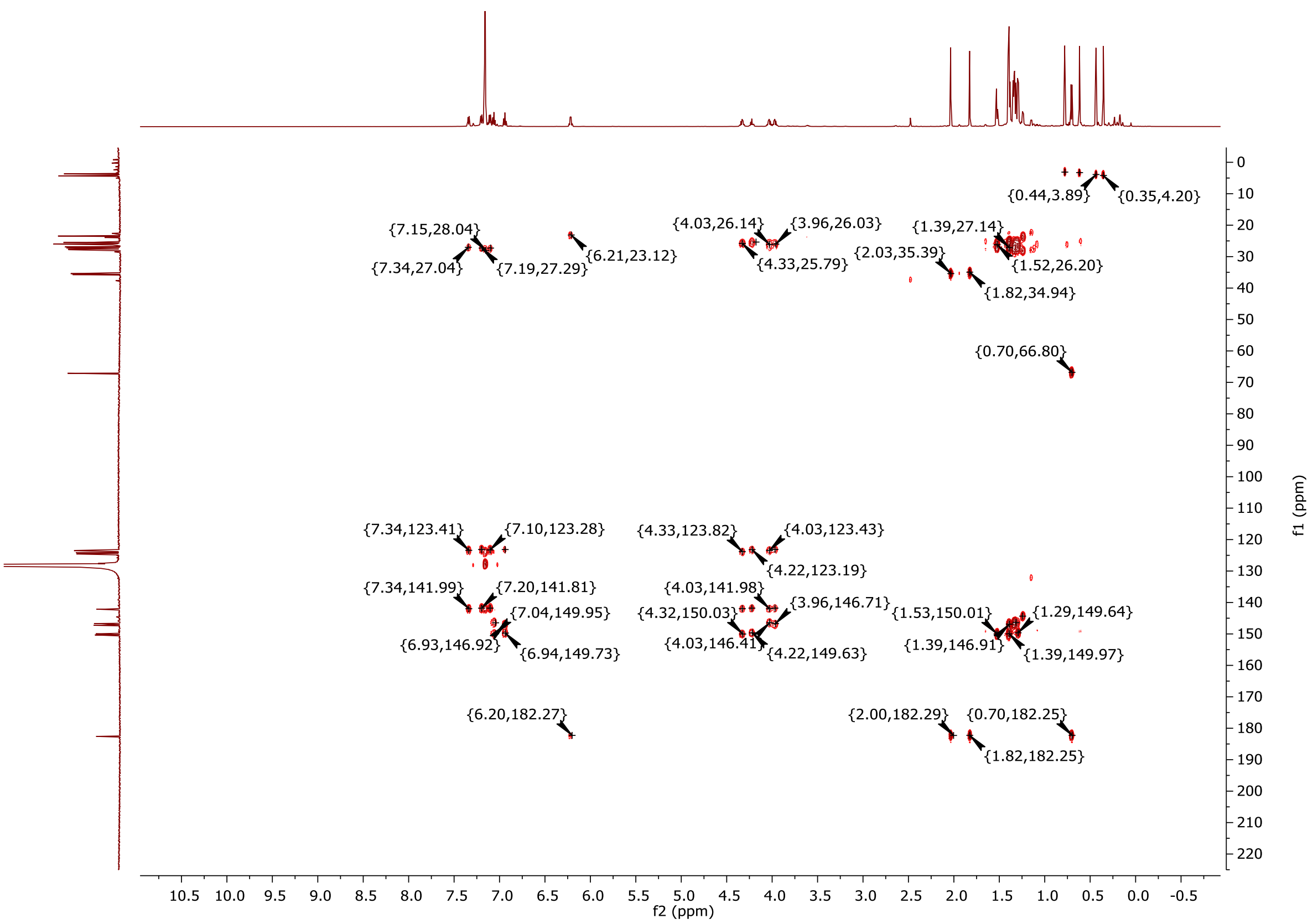

Figure S7: ^{13}C-^{1}H HMBC of **2** (benzene-d_6).

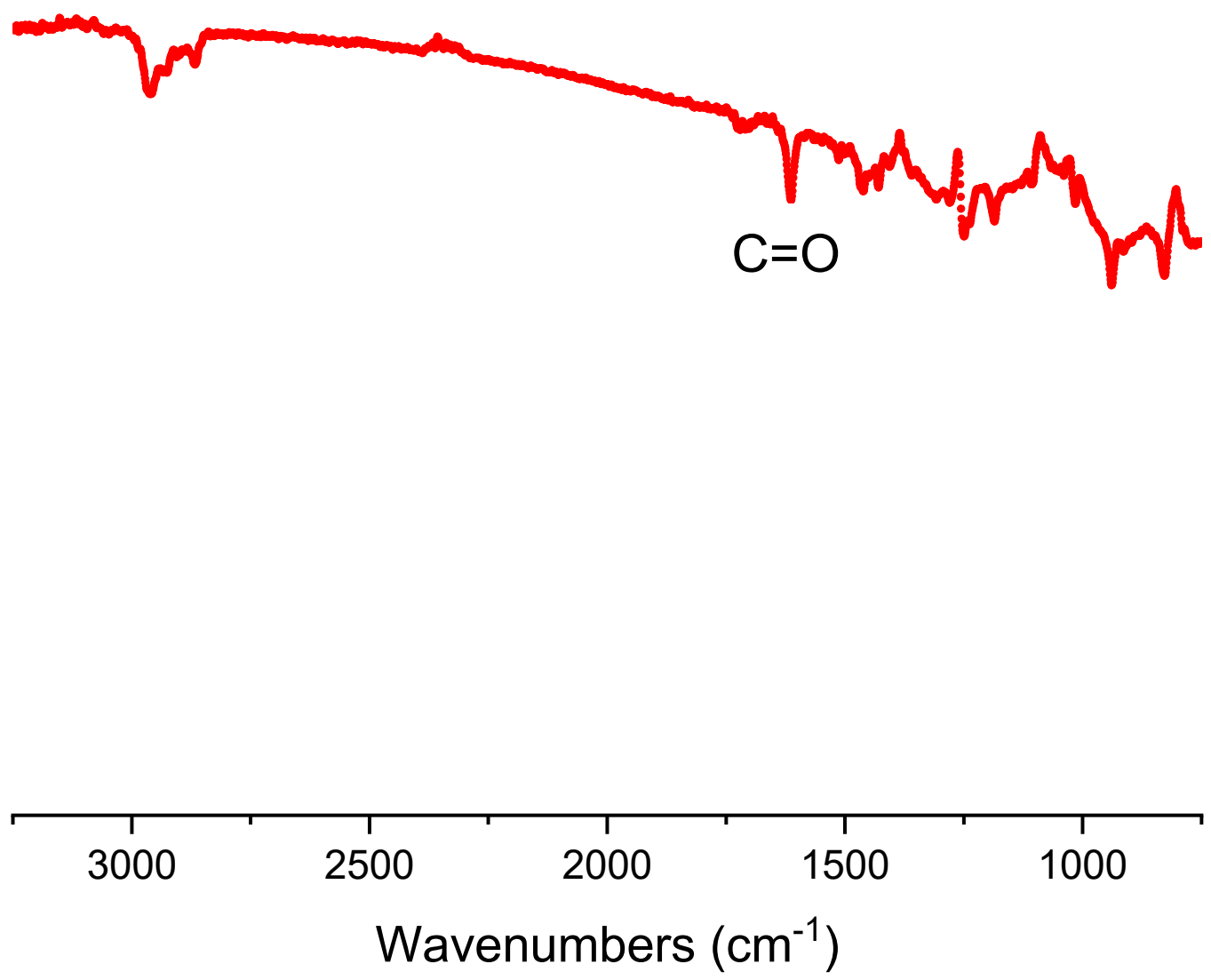

Figure S8: IR (KBr) spectrum of **2**.

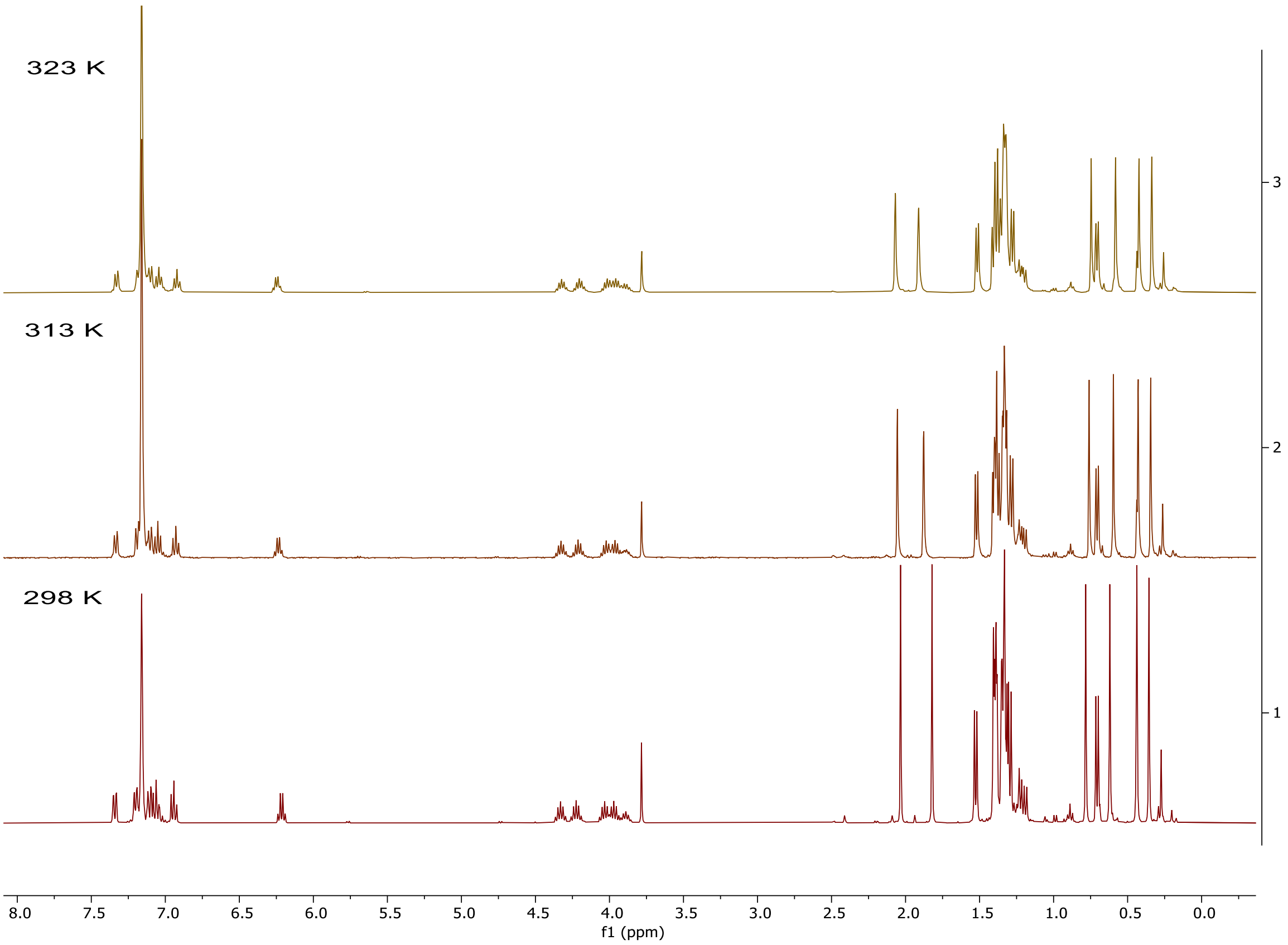

Figure S9: ^{1}H NMR spectra (400 MHz, benzene-d_6) of compound **2**, formed *in situ* from **1** + 0.5 *L*-LA at 323, 313, and 298 K.

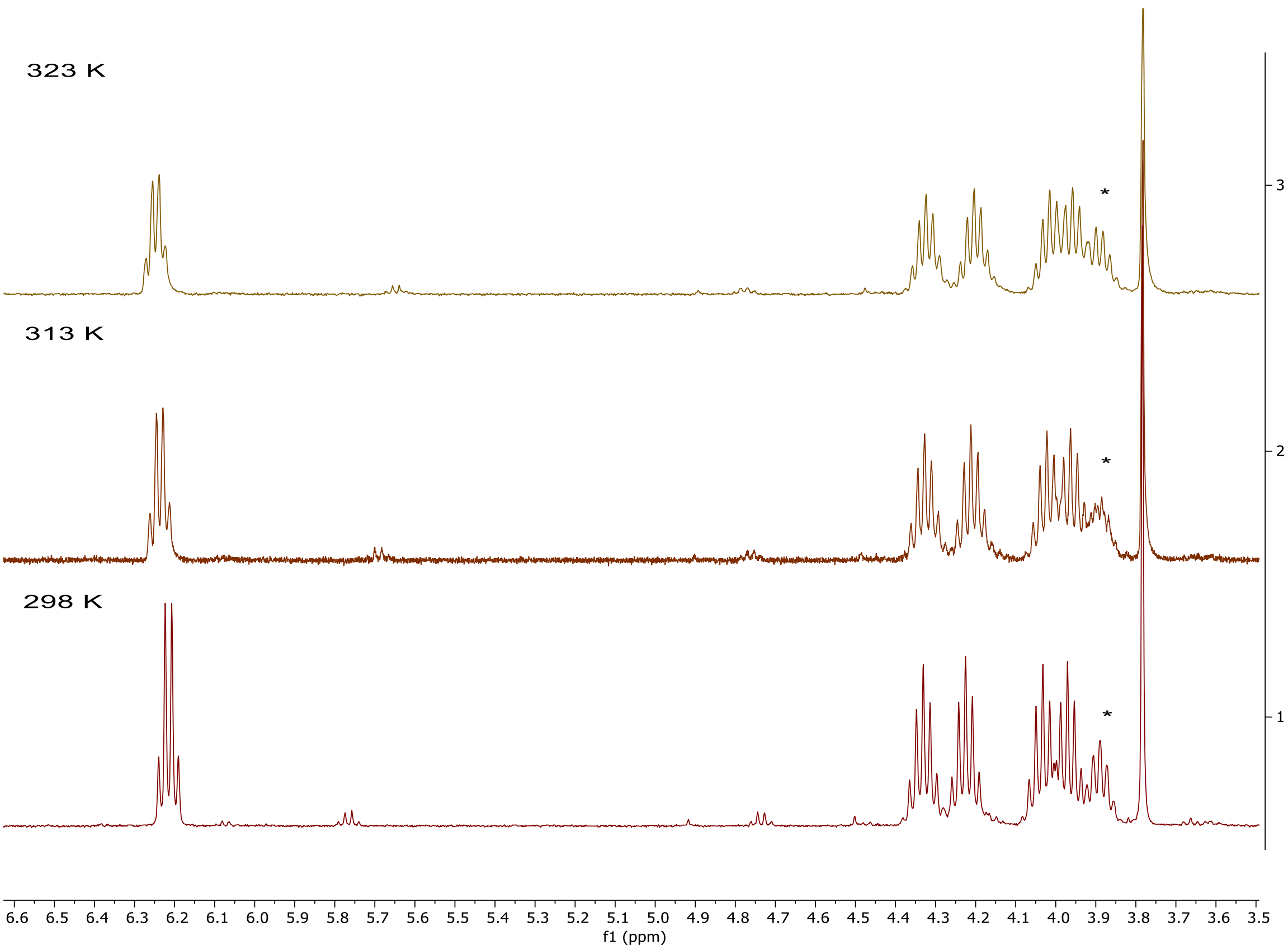

Figure S10: Selected region of the ^{1}H NMR spectra (400 MHz, benzene-d_6) of compound **2**, formed *in situ* from **1**(*) + 0.5 *L*-LA at 323, 313, and 298 K.

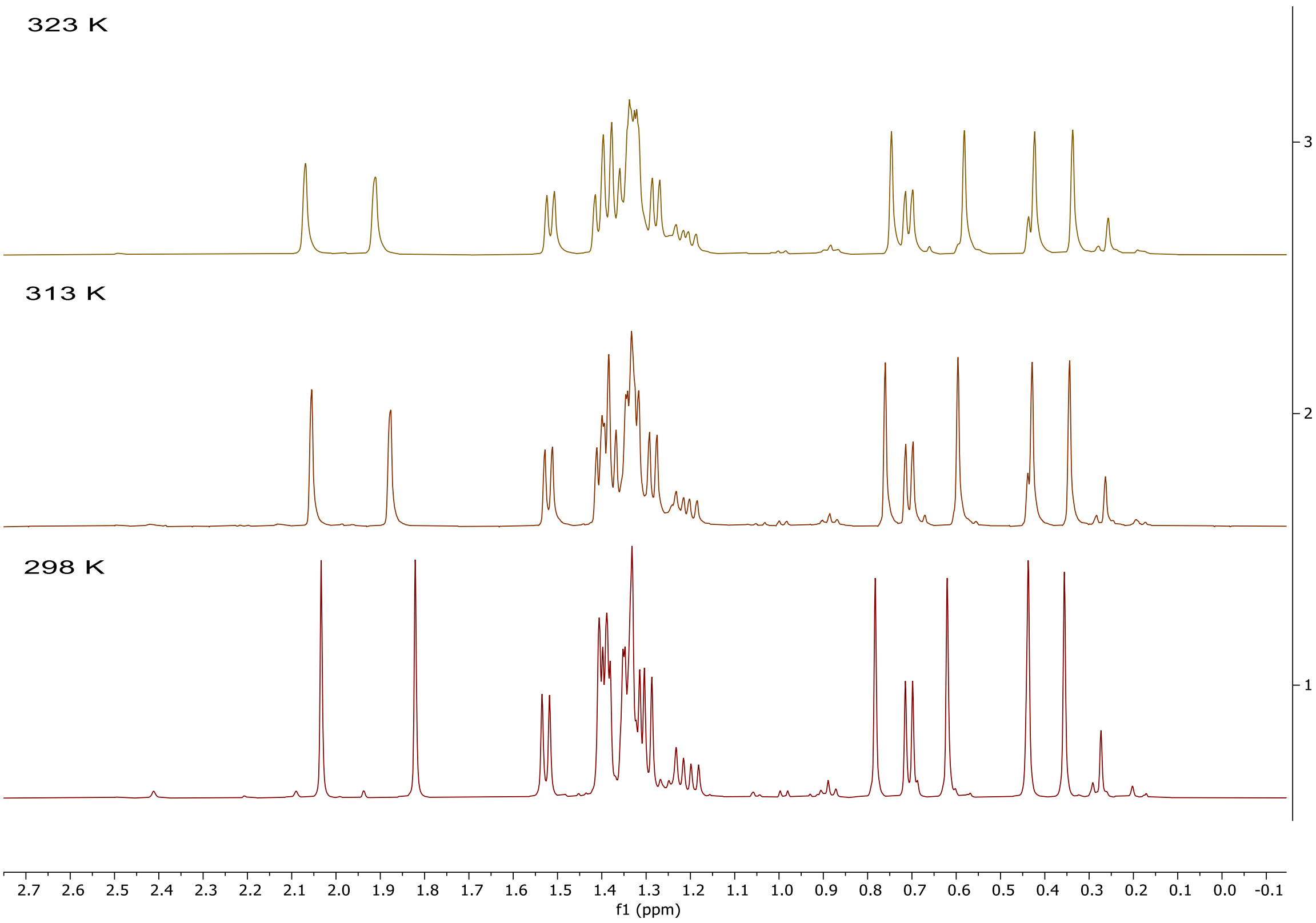

Figure S11: Selected region of the ^{1}H NMR spectra (400 MHz, benzene-d_6) of compound **2**, formed *in situ* from **1**(*) + 0.5 *L*-LA at 323, 313, and 298 K.

2.3. [(NON^{Dipp})Bi(OC$_5$H$_{10}$C(O)NMe$_2$)] (3)

ε-Caprolactone (5.8 μL, 0.052 mmol) was added to a solution of [(NON^{Dipp})Bi(NMe$_2$)] (**1**, 38 mg, 0.052 mmol) in benzene (1 mL). The resulting pale-yellow solution was freeze-dried to afford **3** as a yellow oil. Yield: 45 mg (91%).

^{1}H NMR (benzene-*d*$_6$, 500 MHz): δ δ 7.24 (dd, $^3J_{H-H}$ = 7.6 Hz, $^4J_{H-H}$ = 1.8 Hz, 2H, *m*-C$_6$*H*$_3$), 7.11 (dd, $^3J_{H-H}$ = 7.7 Hz, $^4J_{H-H}$ = 1.8 Hz, 2H, *m*-C$_6$*H*$_3$), 7.04 (t, $^3J_{H-H}$ = 7.6 Hz, 2H, *p*-C$_6$*H*$_3$), 4.15 (sept, $^3J_{H-H}$ = 6.8 Hz, 2H, C*H*Me$_2$), 4.08 (t, $^3J_{H-H}$ = 7.0 Hz, 2H, C*H*$_2$-O), 3.81 (sept, $^3J_{H-H}$ = 6.8 Hz, 2H, C*H*Me$_2$), 2.65 (s, 3H, N*Me*$_2$), 2.24 (s, 3H, N*Me*$_2$), 1.91 (t, $^3J_{H-H}$ = 7.4 Hz, 2H, C*H*$_2$-CO), 1.61 (p, $^3J_{H-H}$ = 7.4 Hz, 2H, C*H*$_2$-CH$_2$-CO), 1.46 (d, $^3J_{H-H}$ = 6.8 Hz, 6H, CH*Me*$_2$), 1.32 (d, $^3J_{H-H}$ = 6.8 Hz, 6H, CH*Me*$_2$), 1.29 (d, $^3J_{H-H}$ = 6.8 Hz, 6H, CH*Me*$_2$), 1.28–1.22 (overlapped with CH*Me*$_2$, 2H, C*H*$_2$-CH$_2$-O), 1.21 (d, $^3J_{H-H}$ = 7.0 Hz, 6H, CH*Me*$_2$), 1.17–1.07 (m, 2H, CH$_2$-C*H*$_2$-CH$_2$), 0.60 (s, 6H, Si*Me*$_2$), 0.28 (s, 6H, Si*Me*$_2$) ppm.

^{13}C{^{1}H} NMR (benzene-*d*$_6$, 126 MHz): δ 171.8 (*C*O), 150.5 (*o*-*C*$_6$H$_3$), 147.1 (*o*-*C*$_6$H$_3$), 140.4 (*i*-*C*$_6$H$_3$), 125.0 (*p*-*C*$_6$H$_3$), 123.9 (*m*-*C*$_6$H$_3$), 124.0 (*m*-*C*$_6$H$_3$), 66.4 (*C*H$_2$-O), 36.5 (N*Me*$_2$), 36.1 (*C*H$_2$-CH$_2$-O), 35.1 (N*Me*$_2$), 33.4 (*C*H$_2$-CO), 28.0 (CH*Me*$_2$), 27.7 (*C*HMe$_2$), 27.0 (CH*Me*$_2$), 26.1 (CH$_2$-*C*H$_2$-CH$_2$), 25.7 (CH*Me*$_2$), 25.6 (*C*H$_2$-CH$_2$-CO), 25.4 (CH*Me*$_2$), 4.1 (Si*Me*$_2$), 3.3 (Si*Me*$_2$) ppm.

IR (KBr): 2360, 2340, 1650 (C=O stretch), 1464, 1431, 1397, 1253, 1185, 1043 cm^{-1}.

Satisfactory CHN analysis could not be achieved due to the persistence of the starting materials in the reaction mixture.

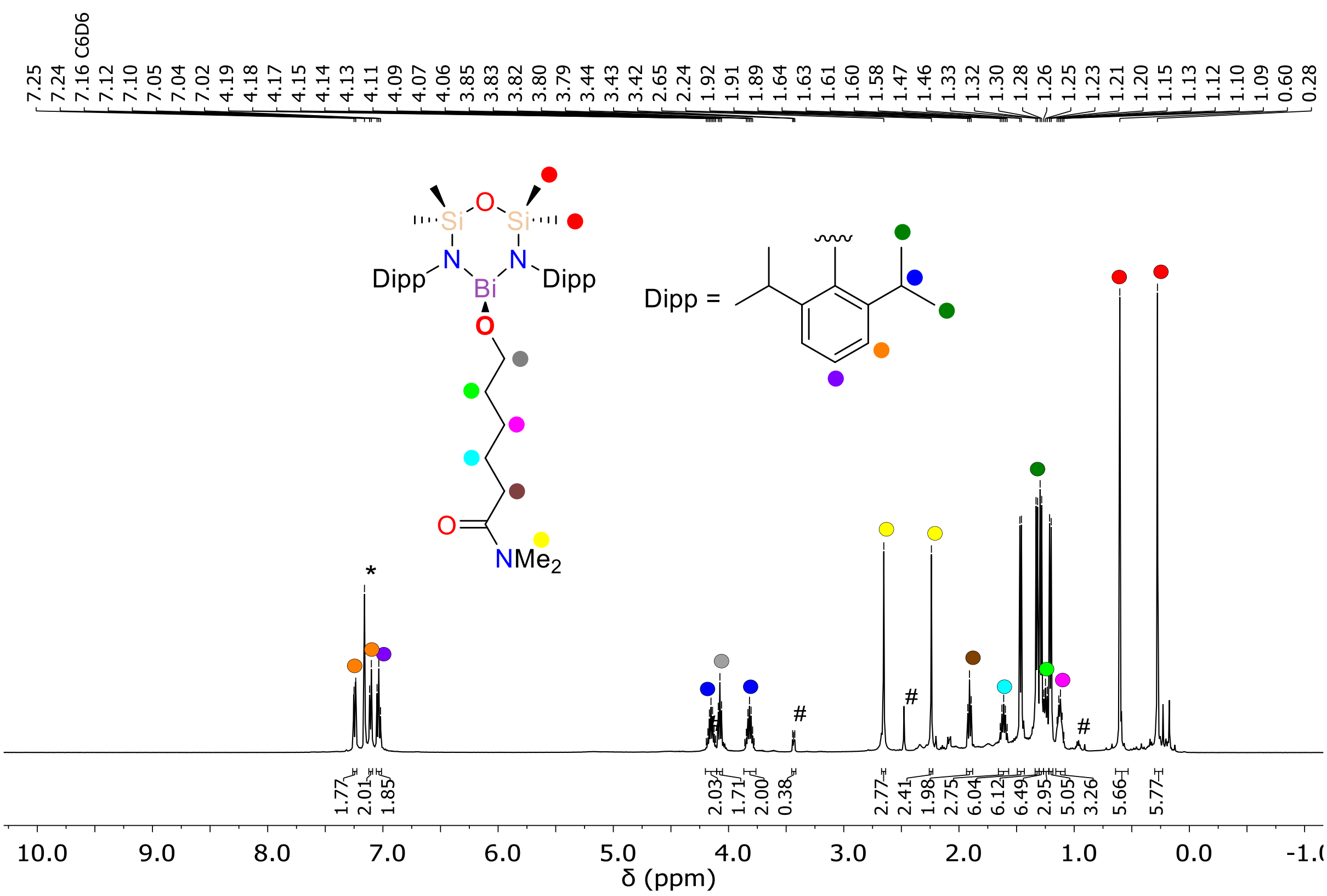

Figure S12: ^{1}H NMR spectrum (C$_6$D$_6$, 600 MHz) of **3**. #Represents residual ε-caprolactone. *Denotes residual protio solvent fraction of C$_6$D$_6$.

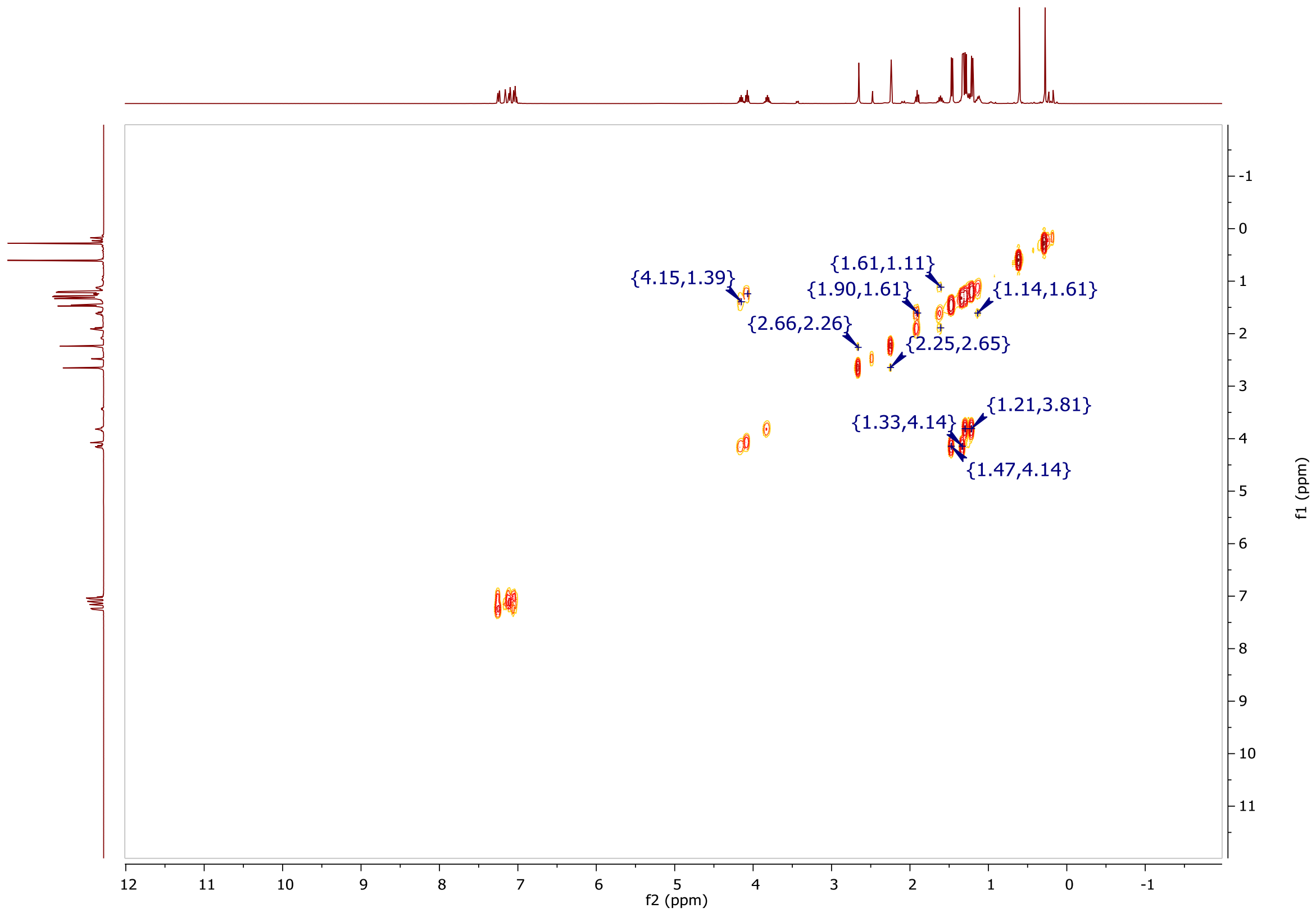

Figure S13: ^{1}H-^{1}H COSY spectrum of **3** (benzene-d_6).

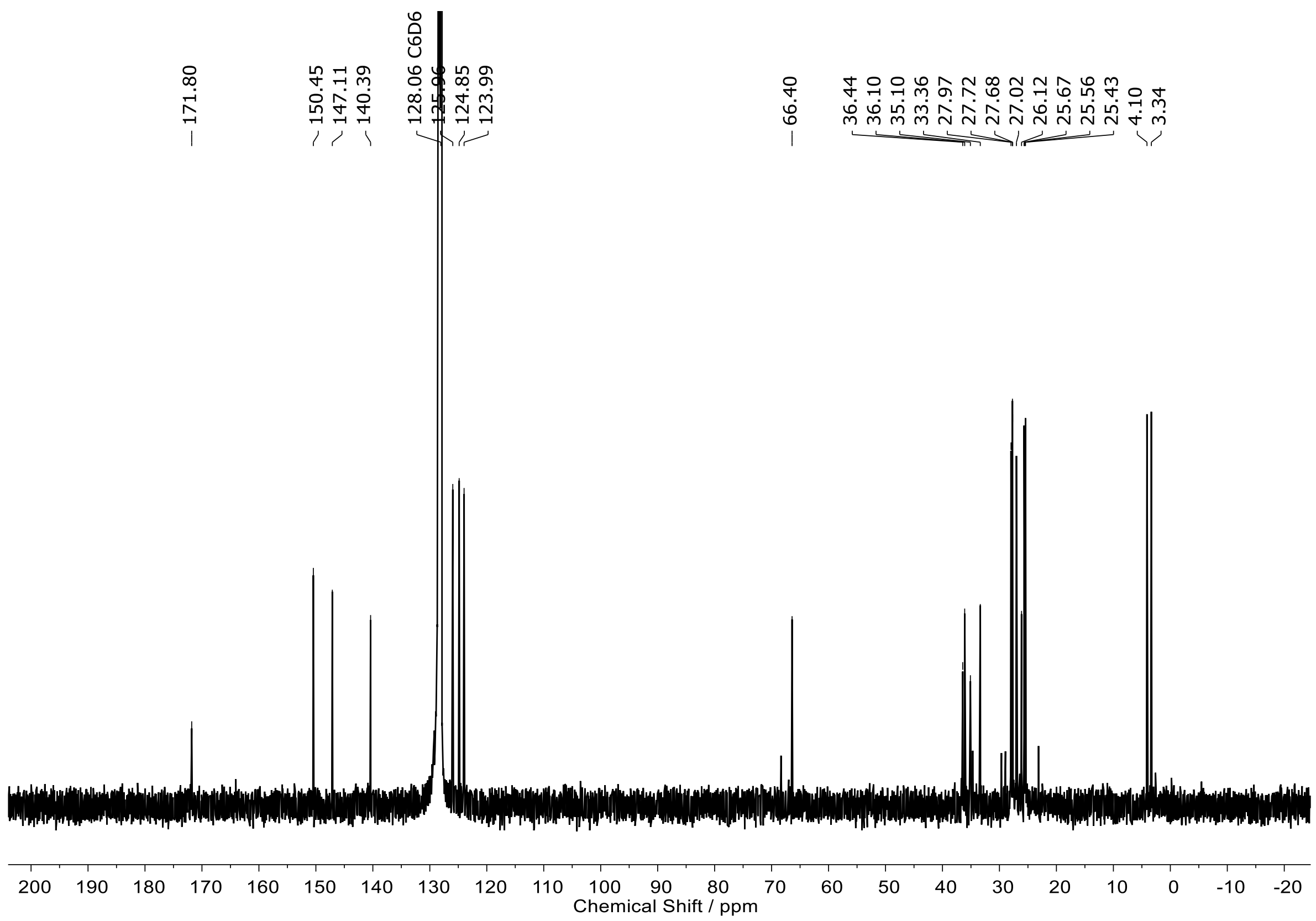

Figure S14: $^{13}C\{^{1}H\}$ NMR spectrum of **3** (126 MHz, benzene-d_6).

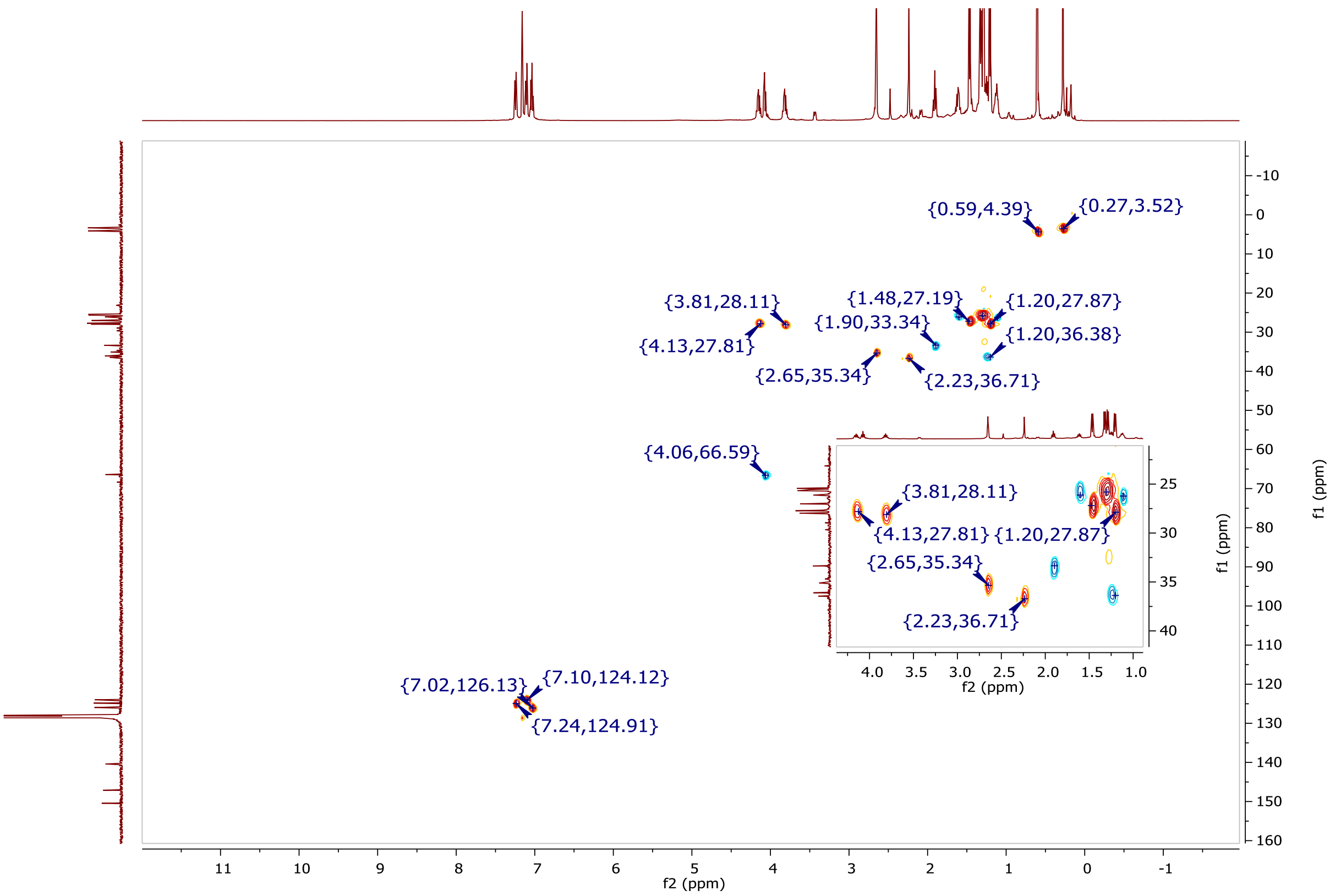

Figure S15: ^{13}C-^{1}H HSQC spectrum of **3** (benzene-d_6).

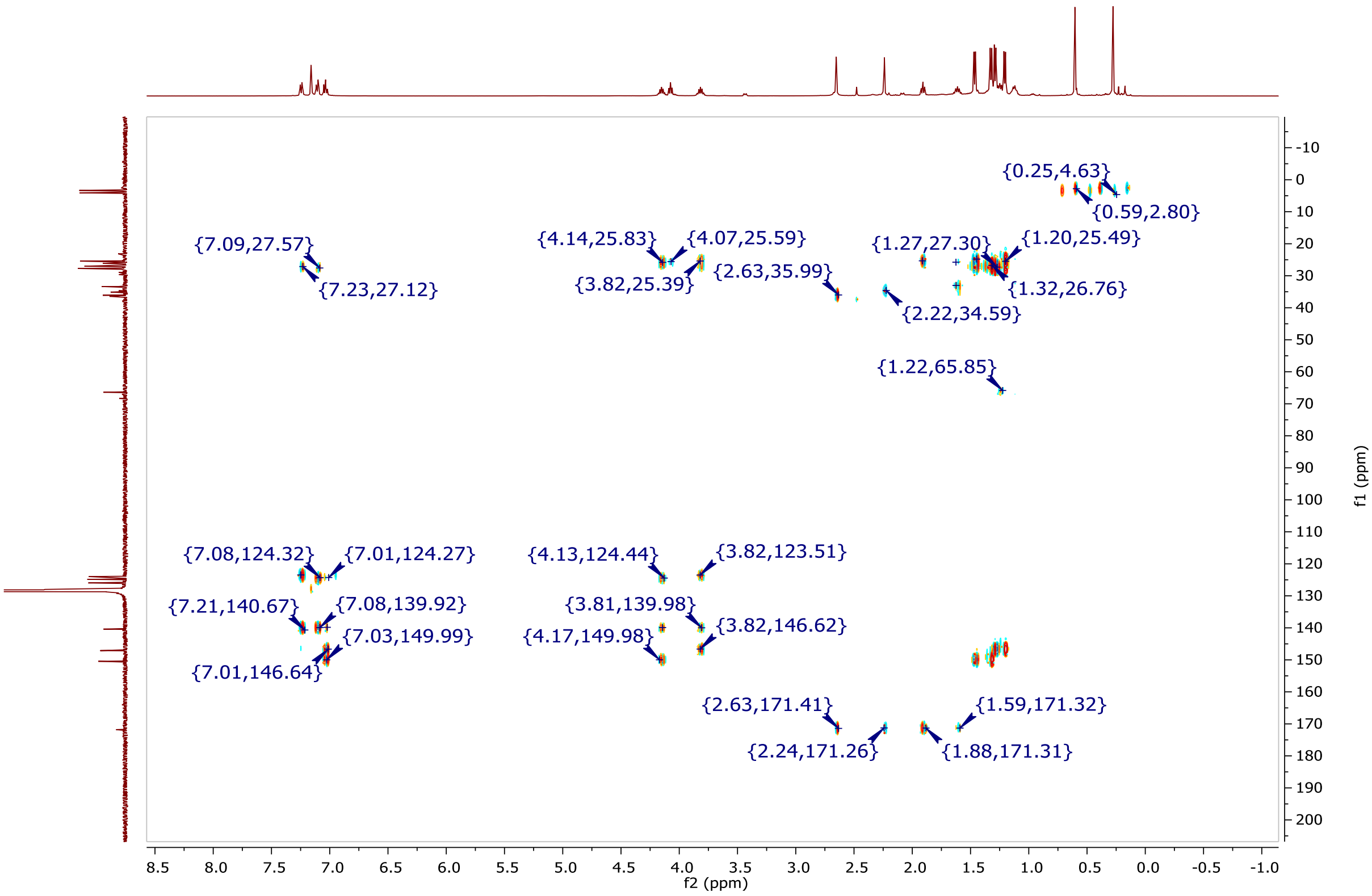

Figure S16: ^{13}C-^{1}H HMBC spectrum of **3** (benzene-d_6).

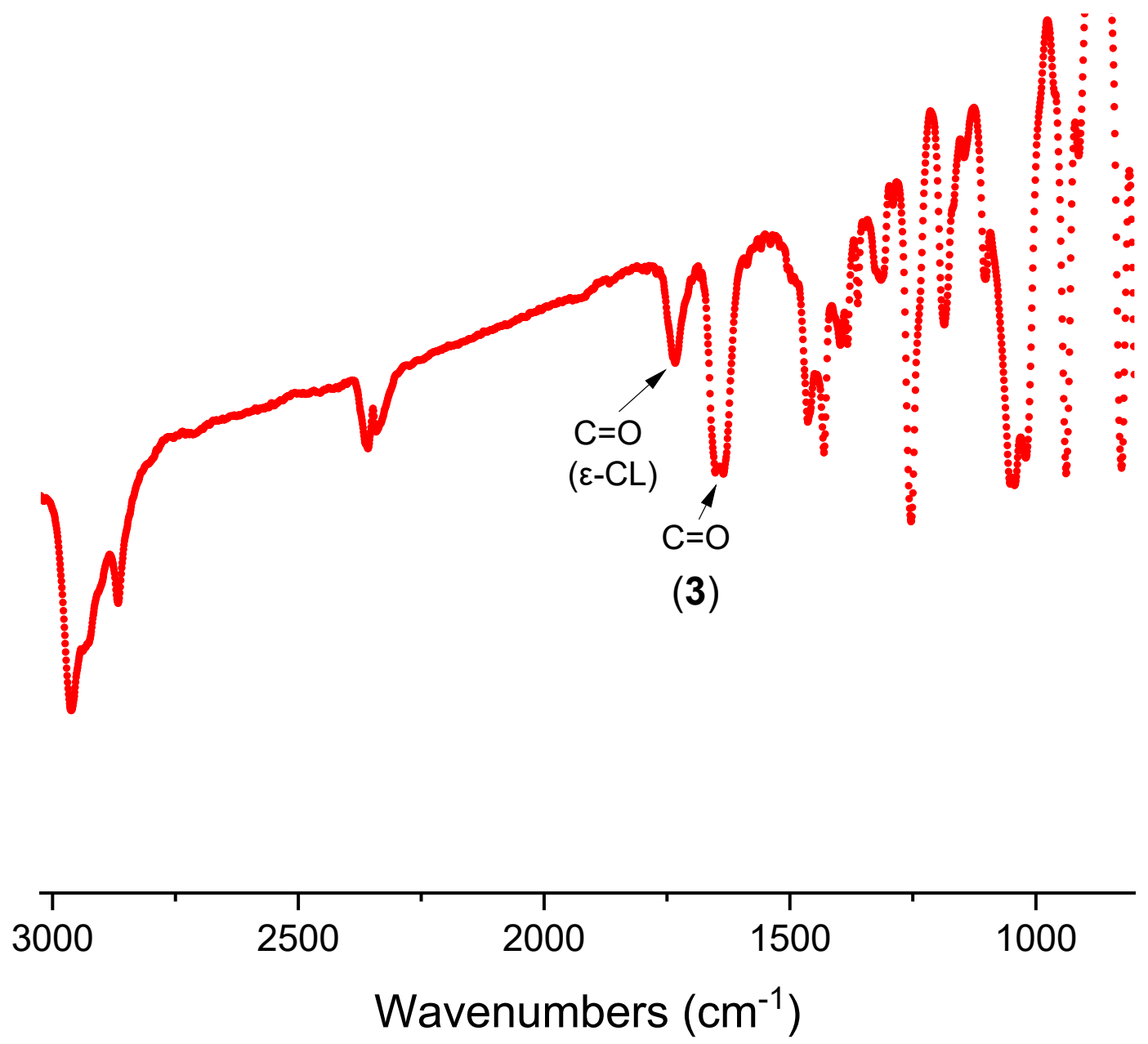

Figure S17. IR (KBr) spectrum of **3**.*Contains residual ε-CL.

2.4. [(NON^{Dipp})Bi(OC$_3$H$_6$C(O)NMe$_2$)] (4)

γ-Butyrolactone (5.75 μL, 0.0670 mmol) was added to a solution of [(NON^{Dipp})Bi(NMe$_2$)] (**1**, 49.3 mg, 0.0670 mmol) in benzene (1 mL). The solution was heated to 50 °C for 16 hours and freeze-dried to afford **4** as a pale-yellow solid. Yield: 48 mg (87%). Colourless block-shaped single crystals suitable for X-ray diffraction analysis were obtained from slow evaporation of a saturated *n*-hexane solution at room temperature.

^{1}H NMR (benzene-*d*$_6$, 400 MHz): δ 7.24 (dd, $^3J_{H-H}$ = 7.6 Hz, $^4J_{H-H}$ = 1.8 Hz, 2H, *m*-C$_6$*H*$_3$), 7.11 (dd, $^3J_{H-H}$ = 7.7 Hz, $^4J_{H-H}$ = 1.8 Hz, 2H, *m*-C$_6$*H*$_3$), 7.01 (t, $^3J_{H-H}$ = 7.6 Hz, 2H, *p*-C$_6$*H*$_3$), 4.30 (t, $^3J_{H-H}$ = 6.2 Hz, 2H, C*H*$_2$-O), 4.19 (sept, $^3J_{H-H}$ = 6.9 Hz, 2H, C*H*Me$_2$), 3.86 (sept, $^3J_{H-H}$ = 6.9 Hz, 2H, C*H*Me$_2$), 2.47 (s, 3H, N*Me*$_2$), 2.15 (s, 3H, N*Me*$_2$), 1.82 (t, $^3J_{H-H}$ = 6.7 Hz, 2H, C*H*$_2$-CO), 1.62 (pent, $^3J_{H-H}$ = 6.4 Hz, 2H, CH$_2$-C*H*$_2$-CH$_2$), 1.45 (d, $^3J_{H-H}$ = 6.7 Hz, 6H, CH*Me*$_2$), 1.35 (d, $^3J_{H-H}$ = 6.8 Hz, 6H, CH*Me*$_2$), 1.30 (d, $^3J_{H-H}$ = 6.7 Hz, 6H, CH*Me*$_2$), 1.26 (d, $^3J_{H-H}$ = 6.9 Hz, 6H, CH*Me*$_2$), 0.62 (s, 6H, Si*Me*$_2$), 0.29 (s, 6H, Si*Me*$_2$) ppm.

^{13}C{^{1}H} NMR (benzene-*d*$_6$, 101 MHz): δ 171.6 (*C*O), 149.9 (*o*-*C*$_6$H$_3$), 147.0 (*o*-*C*$_6$H$_3$), 140.5 (*i*-*C*$_6$H$_3$), 125.6 (*p*-*C*$_6$H$_3$), 124.5 (*m*-*C*$_6$H$_3$), 123.8 (*m*-*C*$_6$H$_3$), 65.2 (*C*H$_2$-O), 36.3 (N*Me*$_2$), 34.8 (N*Me*$_2$), 30.7 (*C*H$_2$), 29.4 (*C*H$_2$-CO), 27.5 (*C*HMe$_2$), 27.4 (*C*HMe$_2$), 27.3 (CH*Me*$_2$), 26.8 (CH*Me*$_2$), 25.6 (CH*Me*$_2$), 25.4 (CH*Me*$_2$), 3.8 (Si*Me*$_2$), 2.9 (Si*Me*$_2$) ppm.

IR (KBr): 2959, 2868, 1630, 1464, 1441, 1259, 1059, 940 cm^{-1}.

Satisfactory CHN analysis could not be achieved due to the persistence of the starting materials in the reaction mixture.

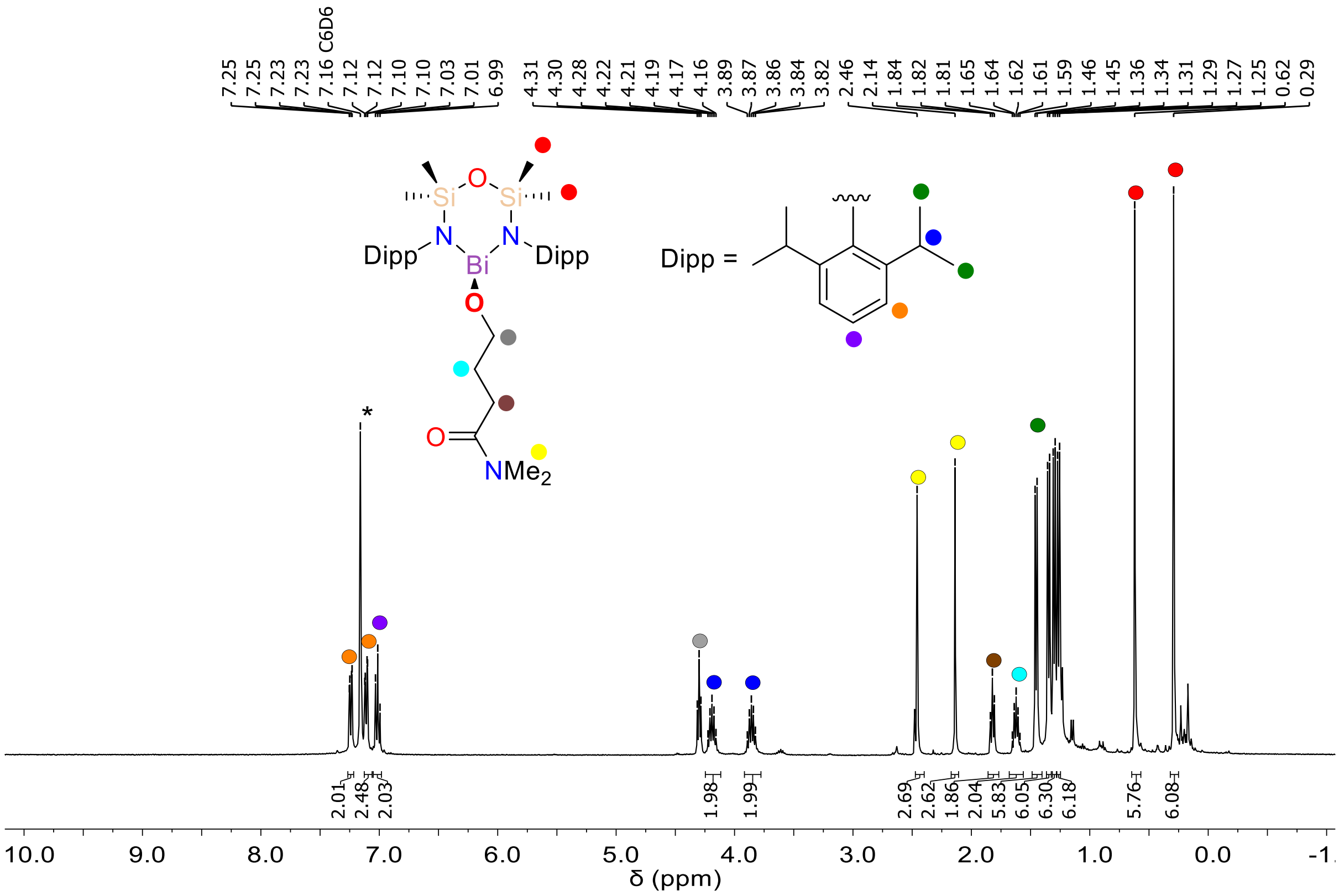

Figure S18: ^{1}H NMR spectrum (C_6D_6, 400 MHz) of **4**. *Denotes residual protio solvent fraction of C_6D_6.

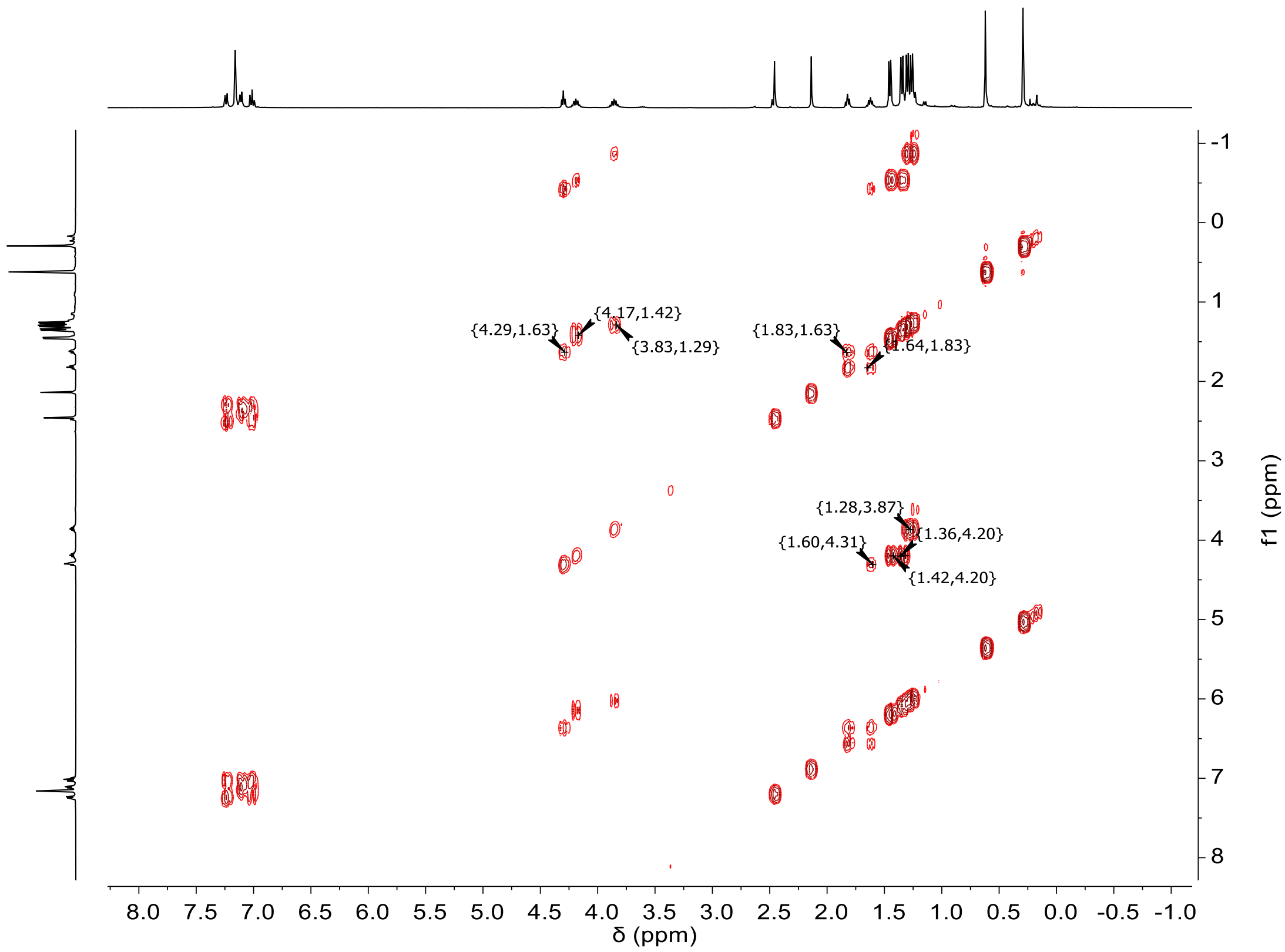

Figure S19: ^{1}H-^{1}H COSY spectrum of **4** (benzene-d_6).

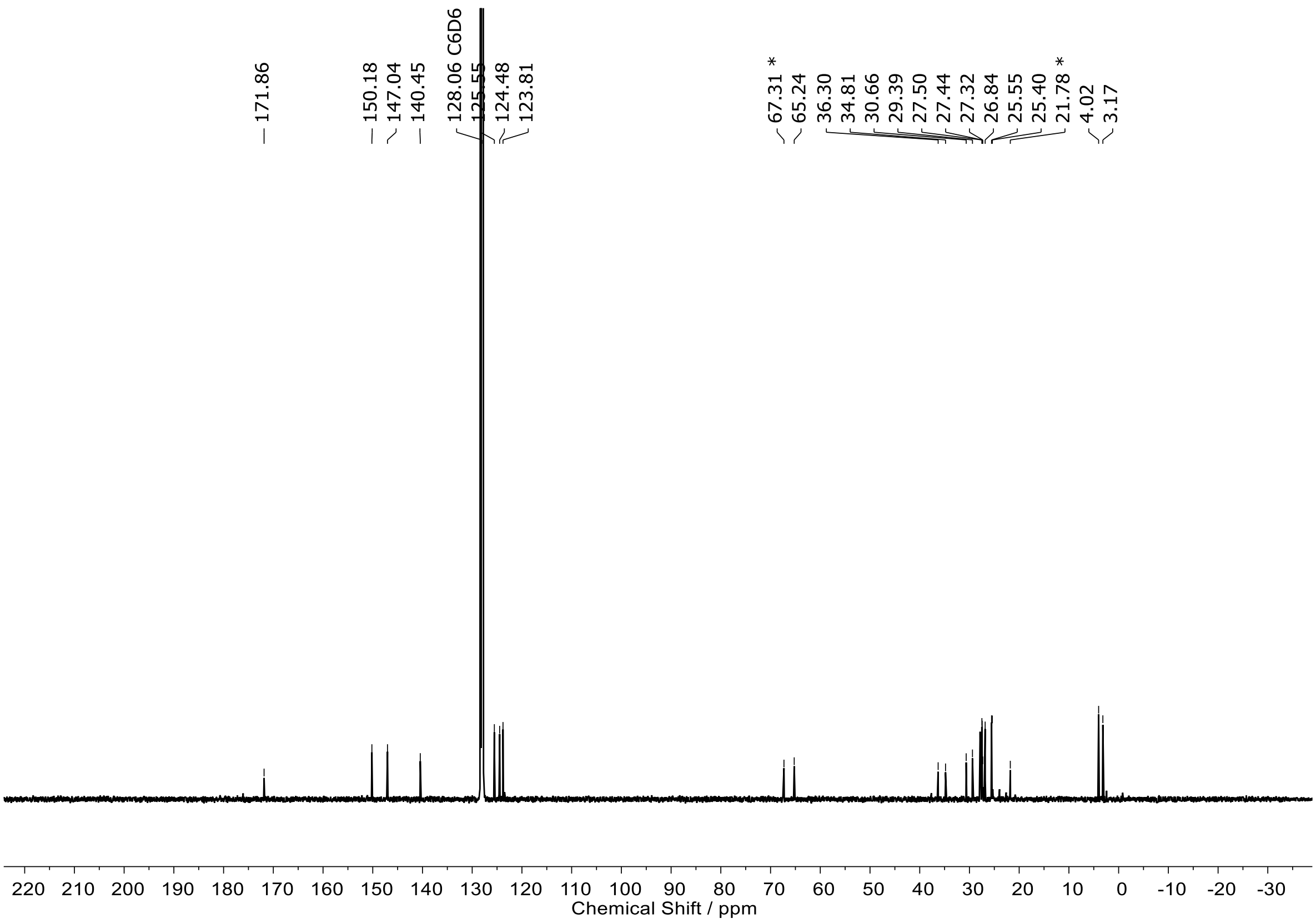

Figure S20: $^{13}C\{^{1}H\}$ NMR spectrum (101 MHz, benzene-d_6) of compound **4**.*Contains residual γ-BL.

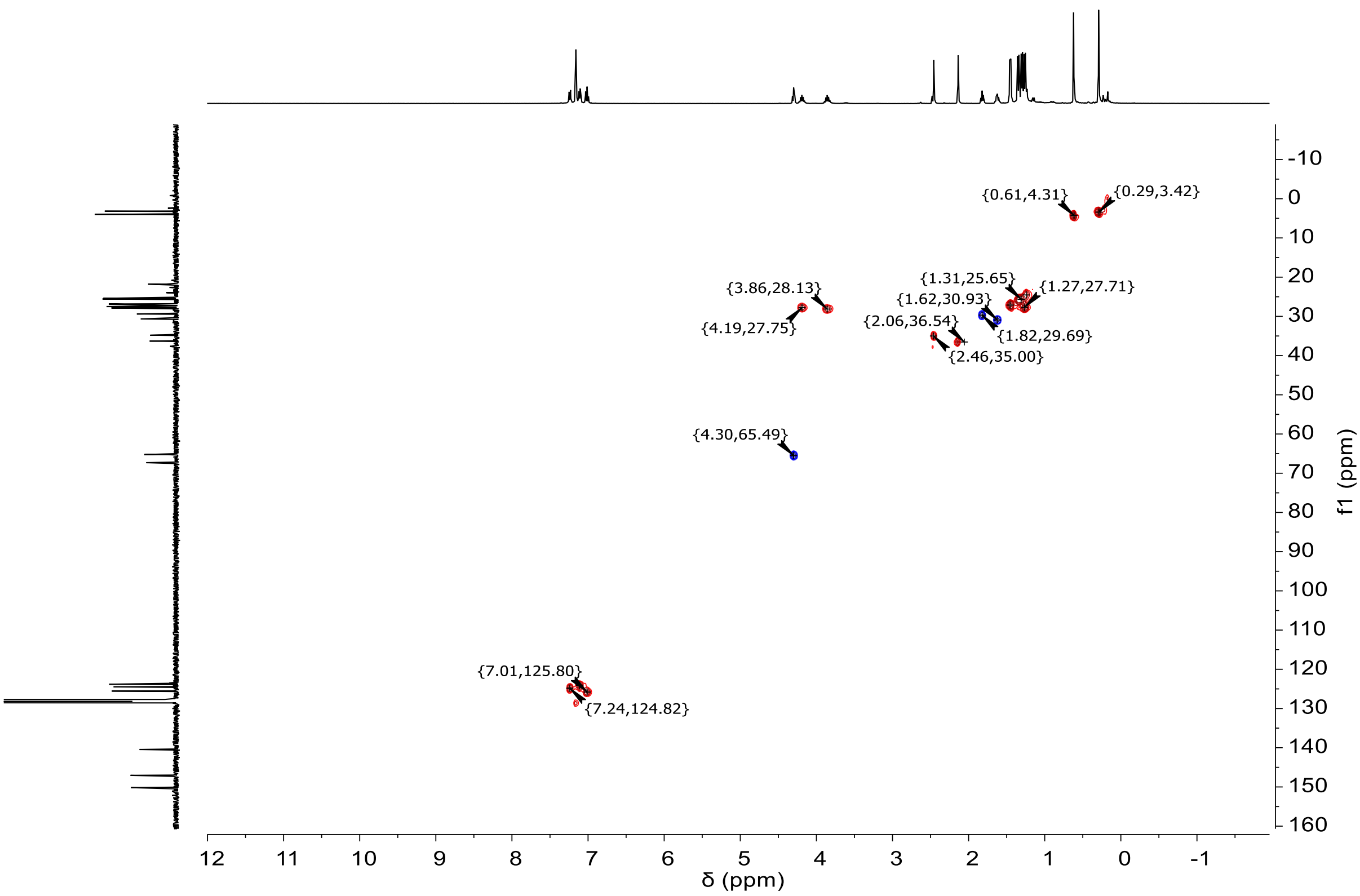

Figure S21: ^{13}C-^{1}H HSQC of **4** (benzene-d_6).

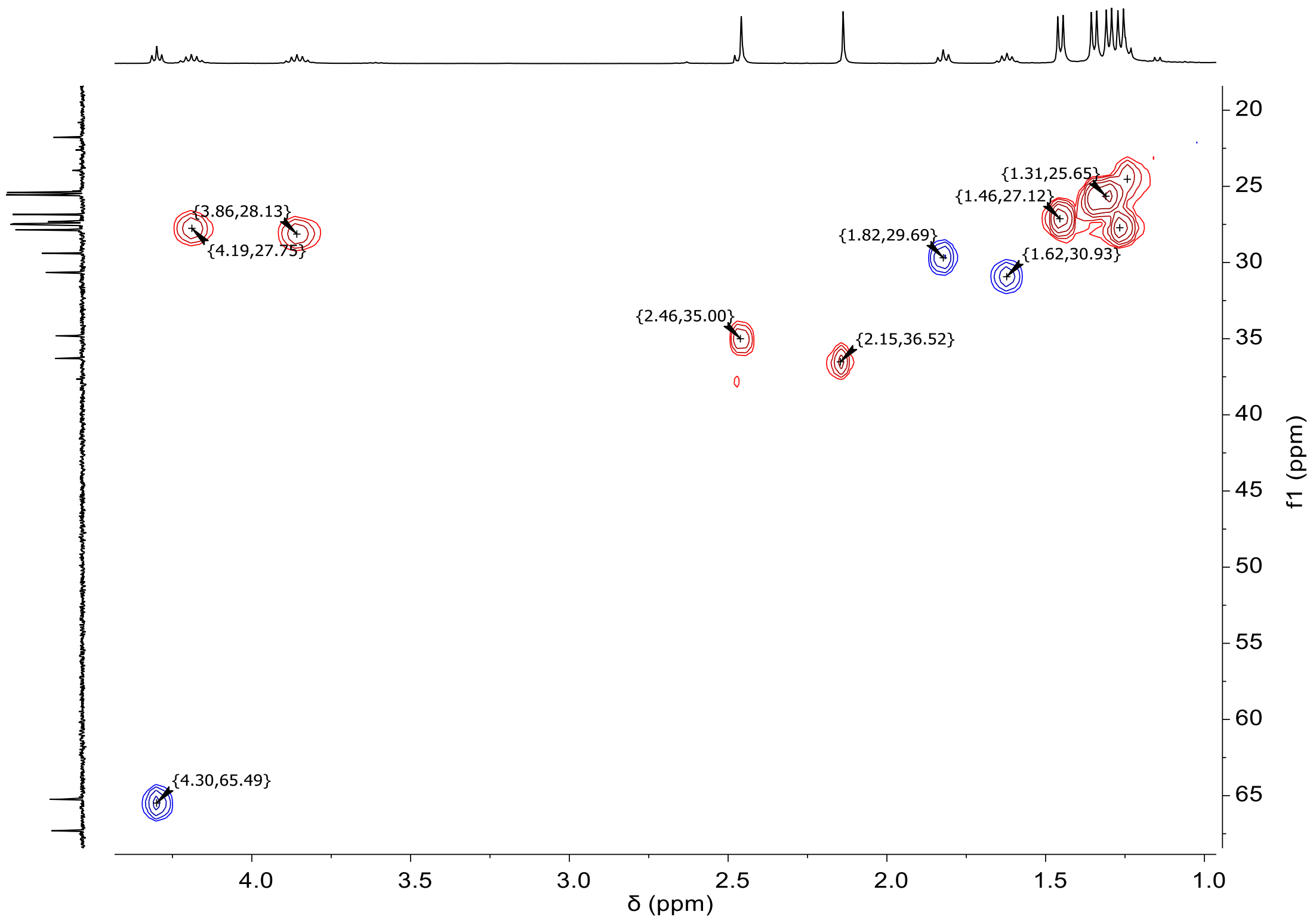

Figure S22: Expanded ^{13}C-^{1}H HSQC spectrum of **4** (benzene-d_6).

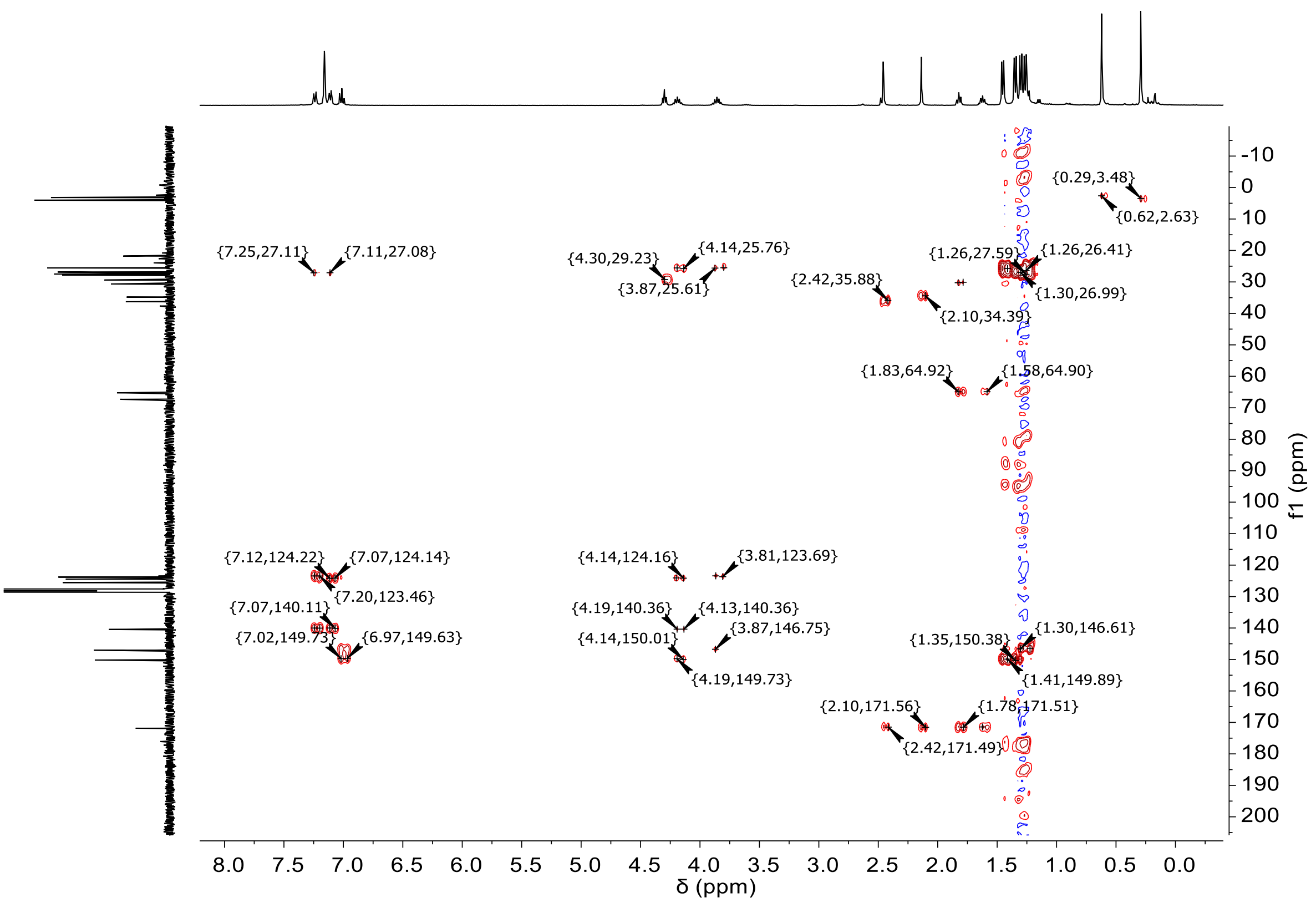

Figure S23: ^{13}C-^{1}H HMBC spectrum of **4** (benzene-d_6).

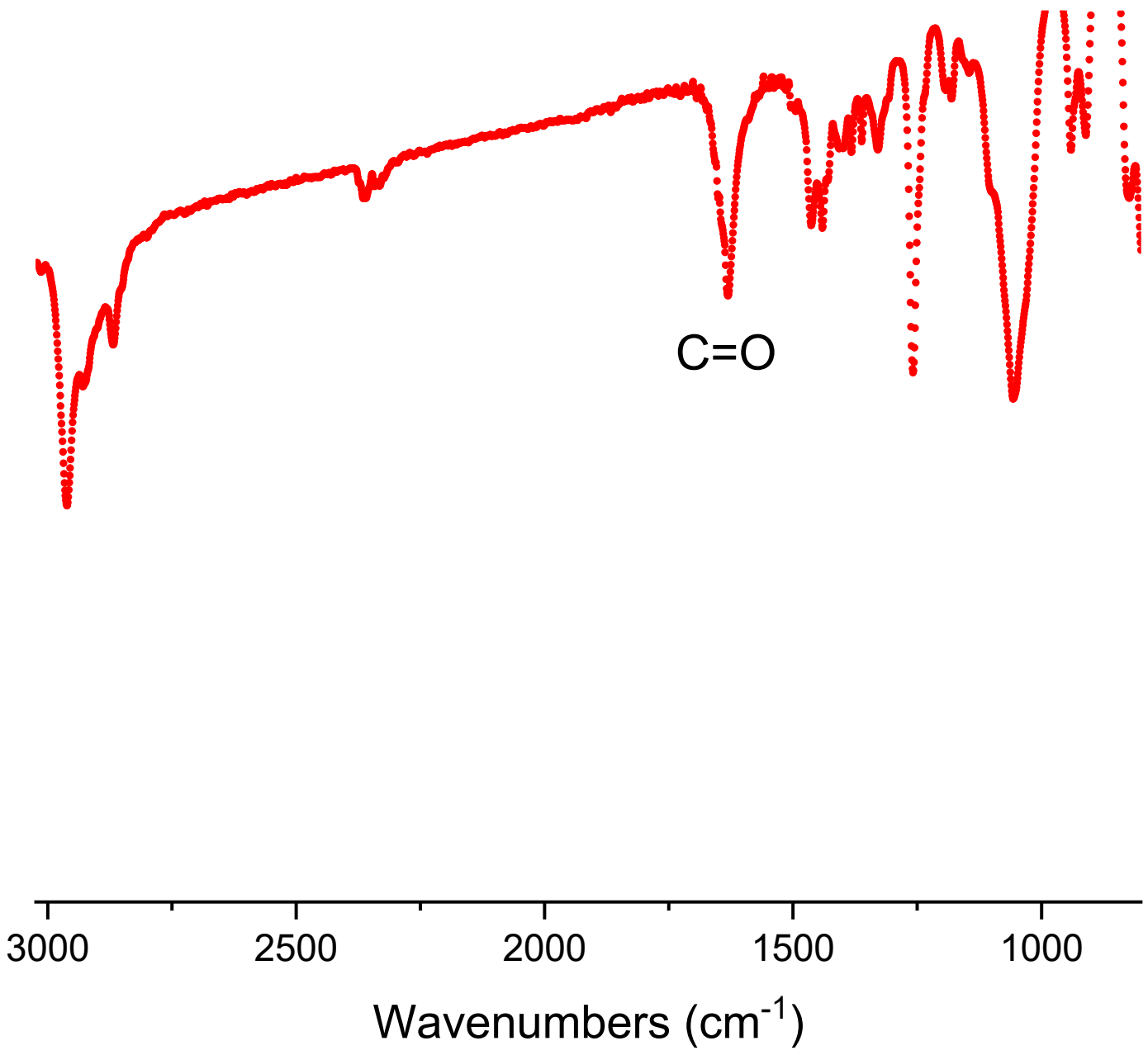

Figure S24: IR (KBr) spectrum of **4**.

3. Polymerisation studies

3.1. General procedure for polymerisation

In a glove box, a J. Young's tap NMR tube was loaded with a monomer: either *L*-, *D*-, *rac*- or *meso*-lactide (43 mg, 0.298 mmol), or ε-caprolactone (33.3 µL, 0.298 mmol), or γ-butyrolactone (22.7 µL, 0.298 mmol). The required amount of the initiator in a deuterated polymerisation solvent (0.6 mL) was then added so that the initial monomer concentration was 0.5 M. Polymerisations were carried out at stated temperatures, monitored by ^{1}H NMR spectroscopy, halted by cooling in an ice bath, and quenched with wet pentane. The solvent was allowed to evaporate from the isolated polymer samples before characterisation.

^{1}H NMR spectroscopy was utilised to determine the monomer conversion based on the integrated area ratios for *methine* proton resonances (lactide polymerisation) and *methylene* CH_2O resonances (ε-caprolactone polymerisation), unless otherwise stated. The isolated samples were analysed by SEC and MALDI-ToF analyses. PLA tacticity was determined by the integrations of the tetrad levels in the ^{1}H{^{1}H} NMR spectrum according to Bernoullian statistics.[5]

3.2. Monomer stereochemistry variation

The ROP activity of **1** towards *L*-, *D*-, *rac*-, and *meso*-lactide stereoisomers was investigated. A linear relationship in the plots of $\ln([LA]_0/[LA]_t)$ *vs*. polymerisation time was confirmed for all four monomers, indicating a first-order dependence on monomer concentration. The polymerisations proceeded without an induction period. The ROP initiation capability of **1** is also superior to that of a series of related β-diketiminato tin(II) amide initiators, which exhibited induction periods of more than 2 hours at 60 °C.[6] The polymerisation of *meso*-lactide displayed the fastest rate (k_{obs} = 0.738 ± 0.005 h^{-1}), consistent with the high ground-state energy of the monomer.[7] *rac*-Lactide was polymerised faster than *D*-lactide, followed by *L*-lactide

polymerisation (k_{obs} = 0.531 ± 0.041 h^{-1} (*rac*-LA) > 0.365 ± 0.022 h^{-1} (*D*-LA) > 0.219 ± 0.008 h^{-1} (*L*-LA)). The polymerisation of *meso*-, *D*-, and *L*-lactide resulted in monomodal, intermediate polymer molecular weight distributions ($Đ_M$ = 1.22–1.36), whilst *rac*-lactide yielded a broadened, bimodal molecular weight distribution ($Đ_M$ = 2.07). Polymers produced from *L*- and *D*-lactide were defined by a relatively good agreement between their $M_{n,SEC}$ and $M_{n,theo}$ values, whereas lower than expected polymer molecular weights were recorded for both *rac*- and *meso*-lactide. These results suggest that the formation of *racemic* and *meso* linkages in the polymer chain was susceptible to transesterification side reactions. Analysis of isolated polymers by ^{1}H{^{1}H} NMR spectroscopy using the Bernoulli model[5] revealed the absence of epimerisation for *L*- and *D*-lactide, with isotactic polymers formed (P_m = 1.00). The polymerisation of *meso*-lactide produced heterotactic-enriched PLA (P_m = 0.86), likely proceeding *via* a chain-end control mechanism, where *meso*-enchainment is favoured by the last monomer unit inserted in the propagating chain. To date, there are very few reported examples of bismuth initiators active for the ROP of *meso*-lactide.[8, 9] While operating *via* an analogous chain end mechanism, a series of pyridine dipyrrolide bismuth aryloxide initiators exhibited a significantly lower heterotactic bias (P_m = 0.54–0.56).[9] In contrast, atactic PLA (P_r = 0.50) was formed during the polymerisation of *rac*-lactide.

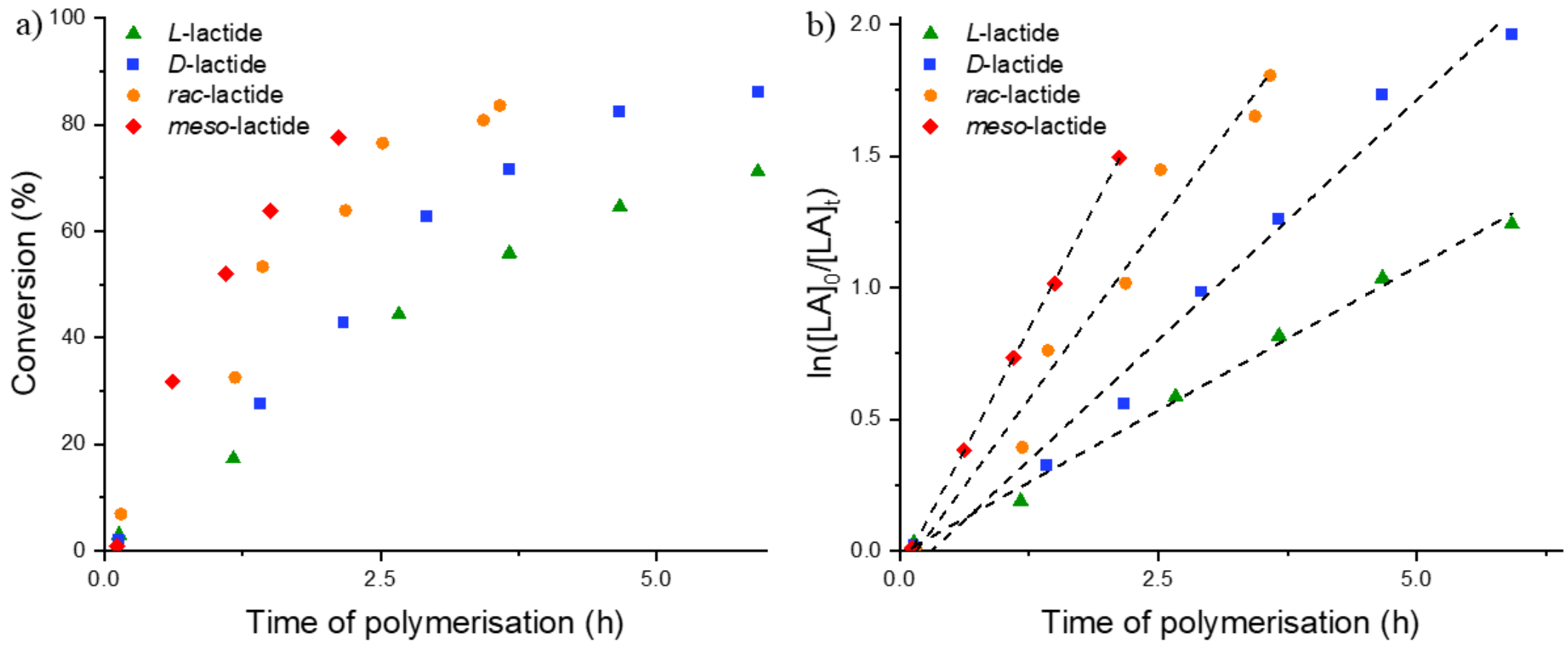

Figure S25: a) Plots of percentage conversion *vs*. polymerisation time for polymerisationof; b) plots of ln([LA]$_0$/[LA]$_t$) *vs*. polymerisation time for lactide polymerisation. Green up triangles: *L*-LA, k_{obs} = 0.219 ± 0.008 h^{-1}, R^2 = 0.994; blue squares: *D*-LA, k_{obs} = 0.365 ± 0.022 h^{-1}, R^2 = 0.982; orange circles: *rac*-LA, k_{obs} = 0.531 ± 0.041 h^{-1}, R^2 = 0.971; red rhombi: *meso*-LA, k_{obs} = 0.738 ± 0.005 h^{-1}, R^2 = 0.999. Polymerisation conditions: benzene-d_6, 50 °C, [LA]$_0$/[Bi]$_0$ = 50:1, and [LA]$_0$ = 0.5 M.

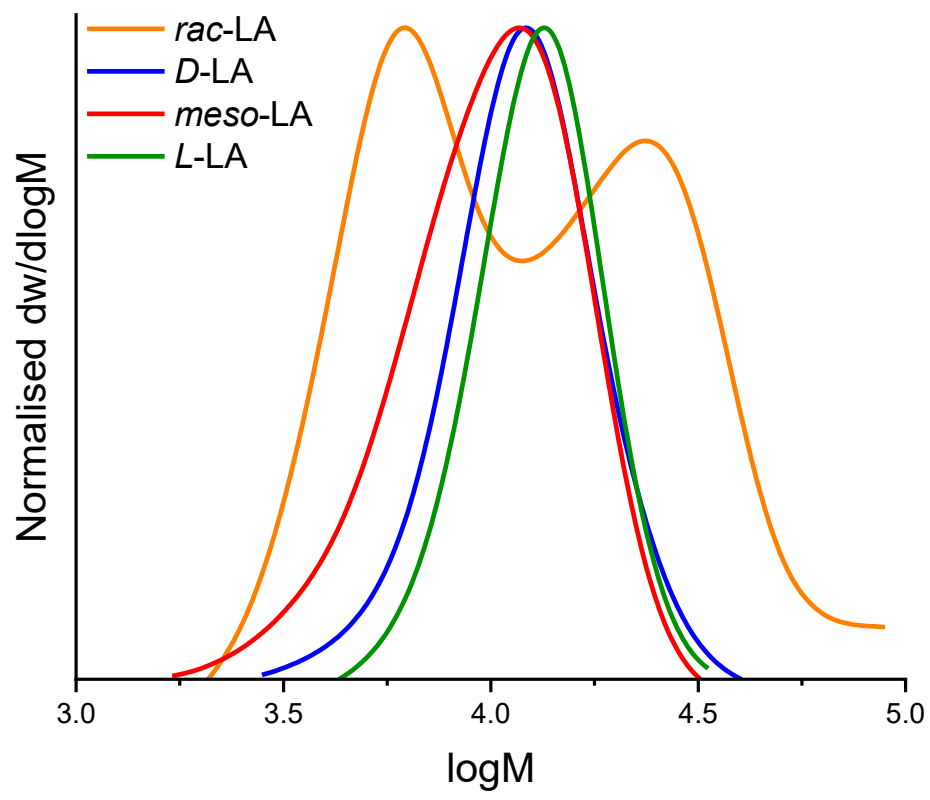

Figure S26: SEC plots of the PLAs produced from *rac*-LA (orange, M_n = 5 060 g mol^{-1}, $Đ_M$ = 2.07); *D*-LA (blue, M_n = 6 170 g mol^{-1}, $Đ_M$ = 1.22); *meso*-LA (red, M_n = 4 770 g mol^{-1}, $Đ_M$ = 1.33); *L*-LA (green, M_n = 5 210 g mol^{-1}, $Đ_M$ = 1.36). Polymerisation conditions: benzene-d_6, 50 °C, $[LA]_0/[Bi]_0$ = 50:1, and $[LA]_0$ = 0.5 M.

Table S1: ROP of *L*-, *D*-, *rac*-, and *meso*-lactide monomers using **1**.[a]

**Monomer**	**Conv.**[b] **(%)**	k_{obs}[c] **(h^{-1})**	R^2[c]	$M_{n,SEC}$[d] **(g mol^{-1})**	$M_{n,theo}$[e] **(g mol^{-1})**	M_w/M_n[d]
L-LA	78	0.219 ± 0.008	0.994	5 210	5 743	1.36
D-LA	96	0.365 ± 0.022	0.982	6 170	6 963	1.22
meso-LA	84	0.738 ± 0.005	0.999	4 770	6 099	1.33
rac-LA	83	0.531 ± 0.041	0.971	5 060	6 026	2.07

[a]Polymerisation conditions: benzene-d_6, 50 °C, $[LA]_0/[Bi]_0$ = 50:1, $[LA]_0$ = 0.5 M. [b]Measured by ^{1}H NMR spectroscopy. [c]Obtained from plots of ln($[LA]_0/[LA]_t$) *vs.* polymerisation time. [d]Determined by SEC analysis calibrated with PS standards in THF with the appropriate Mark-Houwink corrections.[3] [e]Theoretical M_n for PLA = (conv.(%) × $[LA]_0/[Bi]_0$) × 144.13 + (M_w of end groups).

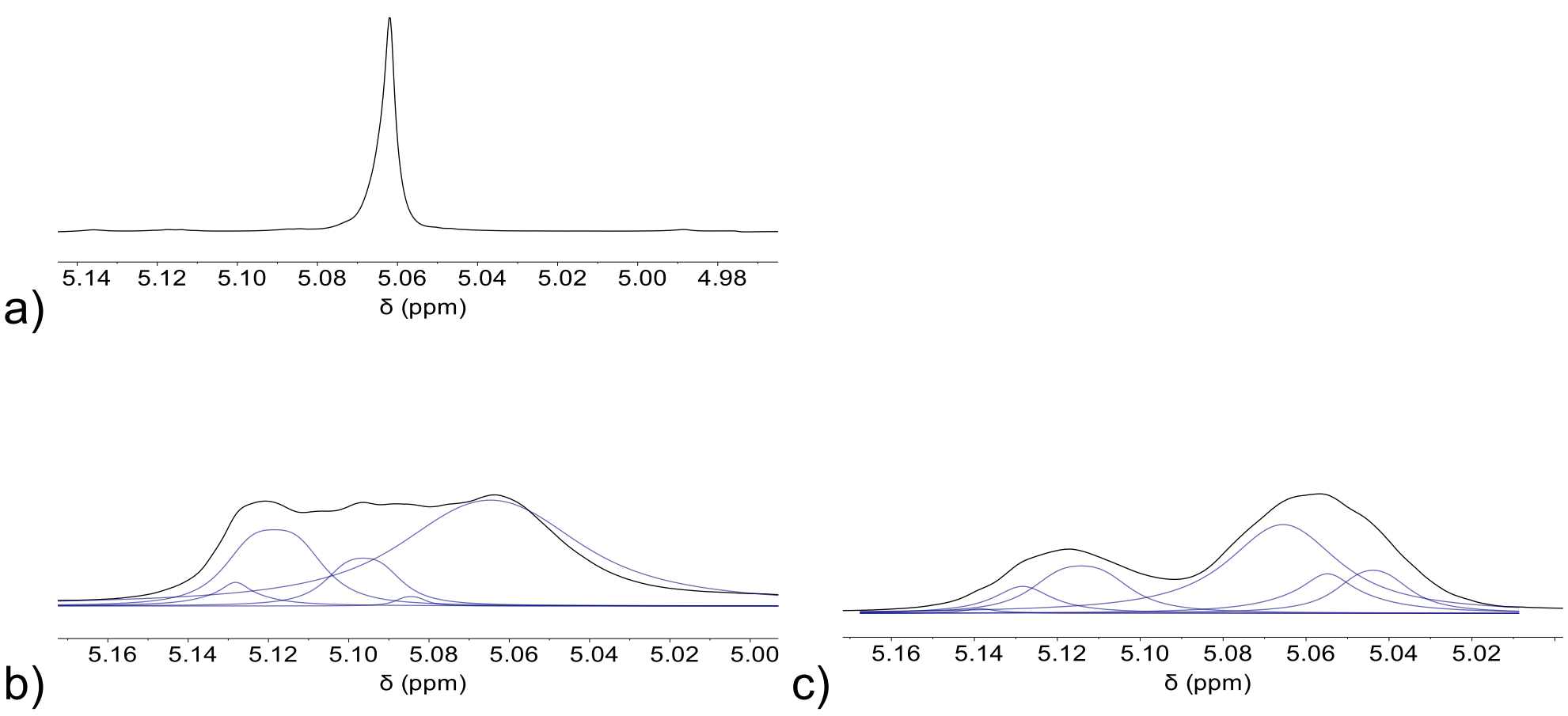

Figure S27: ^{1}H{^{1}H} NMR spectra (chloroform-*d*, 500 MHz) of the *methine* protons in the PLAs produced by **1** from a) *L*- and *D*-LA (P_m = 1.00), b) *meso*-LA (P_m = 0.85), c) *rac*-LA (P_r = 0.50).[5] Polymerisation conditions: benzene-d_6, 50 °C, $[LA]_0/[Bi]_0$ = 50:1, and $[LA]_0$ = 0.5 M.

3.3. Initiator concentration dependence

The rates and control of the polymerisation of *L*-lactide were examined at various initiator loadings ($[L\text{-LA}]_0$:$[\text{Bi}]_0$ = 50:1, 100:1, 200:1, 300:1) in benzene-d_6 at 50 °C. A plot of k_{obs} *vs.* $[\text{Bi}]_0$ was constructed, and the k_p value for **1** was determined to be 24.68 ± 1.82 M^{-1} h^{-1} (R^2 = 0.988). A linear relationship of −ln(k_{obs}) *vs.* −ln($[\text{Bi}]_0$) displayed a gradient of 1.45 ± 0.21 (R^2 = 0.961), indicative of a first-order dependence on initiator within the associated error. A non-integer dependence of up to 1.56 has been previously attributed to the aggregation of catalytically active species resulting in different levels of ROP activity, although its mechanistic interpretation is not well-understood.[10-13] The idealised overall rate law for the ROP of *L*-lactide is therefore described by **Equation S1:**

$$-\frac{d[\text{LA}]}{dt} = k_{\text{p}}[\text{LA}]_0[\mathbf{1}]_0$$

Where k_p = 24.68 ± 1.82 M^{-1} h^{-1}.

k_p = propagation rate constant, $[\text{LA}]_0$ = initial lactide concentration, $[\mathbf{1}]_0$ = initial initiator concentration.

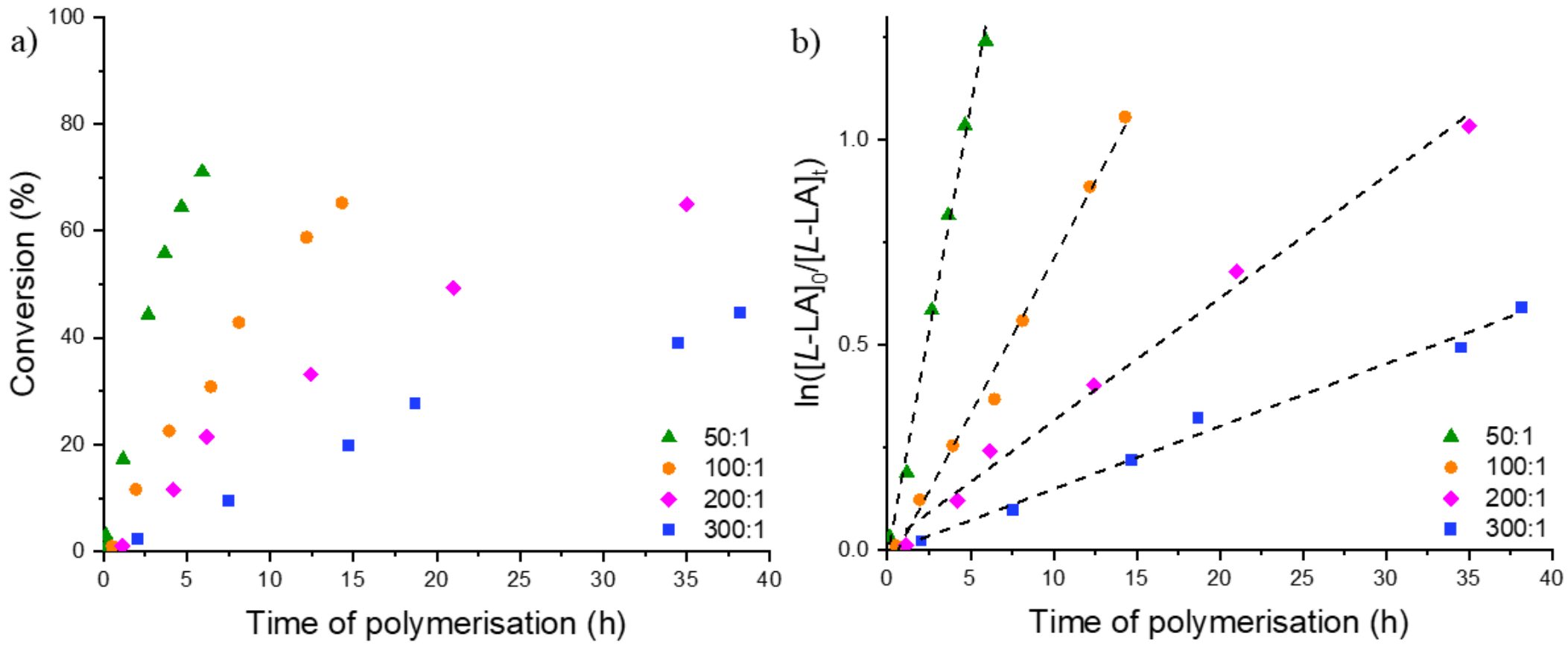

Figure S28: a) Plots of percentage conversion *vs.* polymerisation time for *L*-lactide polymerisation; b) plots of ln($[L\text{-LA}]_0/[L\text{-LA}]_t$) *vs.* polymerisation time for *L*-lactide polymerisation. Green up triangles: $[L\text{-LA}]_0$:$[\mathbf{1}]_0$ = 50:1, k_{obs} = 0.219 ± 0.008 h^{-1}, R^2 = 0.994; orange circles: $[L\text{-LA}]_0$:$[\mathbf{1}]_0$ = 100:1, k_{obs} = 0.076 ± 0.003 h^{-1}, R^2 = 0.993; pink rhombi: $[L\text{-LA}]_0$:$[\mathbf{1}]_0$ = 200:1, k_{obs} = 0.030 ± 0.001 h^{-1}, R^2 = 0.992; blue squares: $[L\text{-LA}]_0$:$[\mathbf{6}]_0$ = 300:1, k_{obs} = 0.015 ± 0.001 h^{-1}, R^2 = 0.988. Polymerisation conditions: benzene-d_6, 50 °C, $[L\text{-LA}]_0$ = 0.5 M.

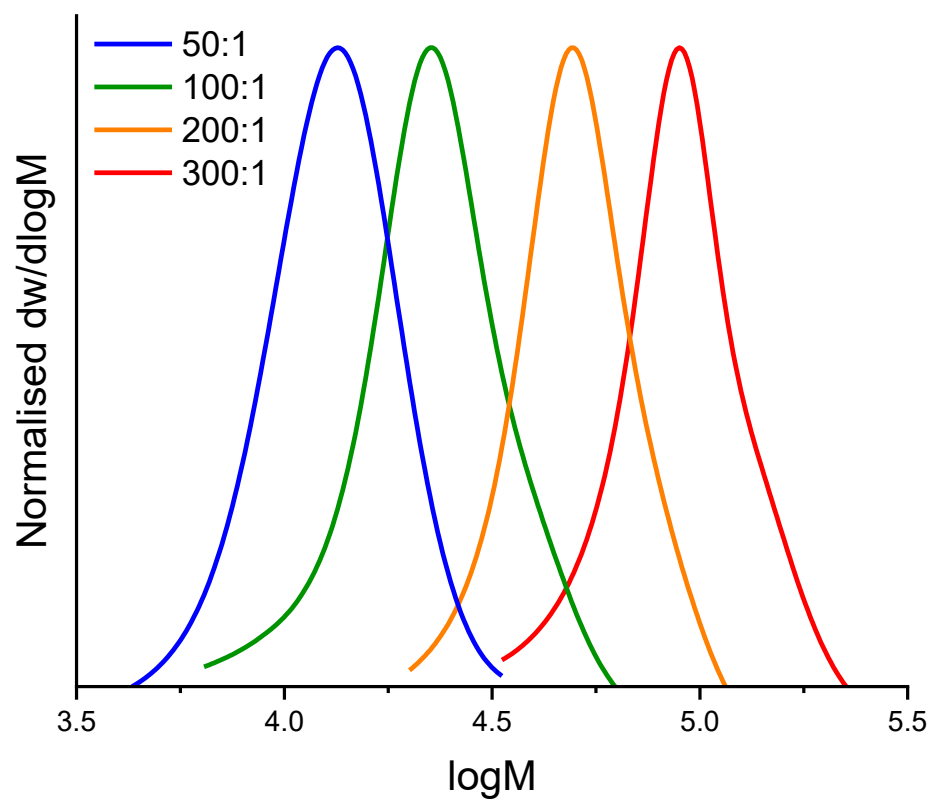

Figure S29: SEC plots of the PLLAs produced with $[L\text{-LA}]_0$:$[6]_0$ = 50:1 (blue, M_n = 5 210 g mol^{-1}, $Đ_M$ = 1.36); $[L\text{-LA}]_0$:$[6]_0$ = 100:1 (green, M_n = 11 520 g mol^{-1}, $Đ_M$ = 1.22); $[L\text{-LA}]_0$:$[6]_0$ = 200:1 (orange, M_n = 26 850 g mol^{-1}, $Đ_M$ = 1.14); $[L\text{-LA}]_0$:$[6]_0$ = 300:1 (red, M_n = 35 460 g mol^{-1}, $Đ_M$ = 1.15). Polymerisation conditions: benzene-d_6, 50 °C, $[L\text{-LA}]_0$ = 0.5 M.

Table S2: ROP of *L*-lactide at different $[L\text{-LA}]_0/[\text{Bi}]_0$ ratios using **1**.[a]

$[L\text{-LA}]_0/[\text{Bi}]_0$	**Conv.**[b] **(%)**	k_{obs}[c] **(h^{-1})**	R^2[c]	$M_{n,SEC}$[d] **(g mol^{-1})**	$M_{n,theo}$[e] **(g mol^{-1})**	M_w/M_n[d]
50:1	78	0.219 ± 0.008	0.994	5 200	5 400	1.36
100:1	84	0.076 ± 0.003	0.993	11 500	12 200	1.22
200:1	80	0.030 ± 0.001	0.992	26 800	23 100	1.14
300:1	77	0.015 ± 0.001	0.988	35 500	33 300	1.15

[a]Polymerisation conditions: benzene-d_6, 50 °C, $[L\text{-LA}]_0$ = 0.5 M. [b]Measured by ^{1}H NMR spectroscopy. [c]Obtained from plots of $\ln([L\text{-LA}]_0/[L\text{-LA}]_t)$ *vs.* polymerisation time. [d]Determined by SEC analysis calibrated with PS standards in THF with the appropriate Mark-Houwink corrections.[3] [e]Theoretical M_n for PLA = (conv.(%) × $[L\text{-LA}]_0/[\text{Bi}]_0$) × 144.13 + ($M_w$ of end groups).

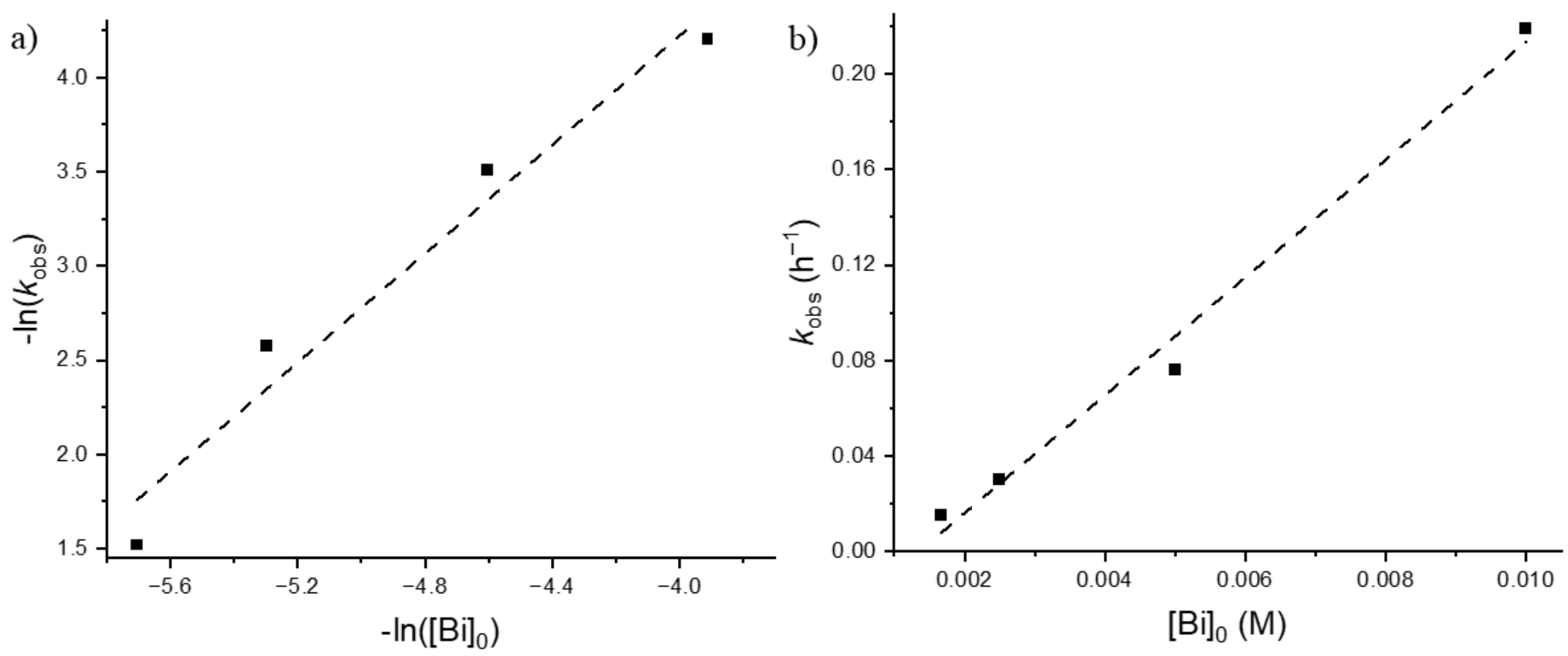

Figure S30: a) Plot of $-\ln(k_{obs})$ *vs.* $-\ln([\text{Bi}]_0)$ for *L*-lactide polymerisation using **1**; slope = 1.45 ± 0.21 (R^2 = 0.961); b) plot of k_{obs} *vs.* $[\text{Bi}]_0$ for *L*-lactide polymerisation using **1**; slope = 24.68 ± 1.82 M^{-1} h^{-1} (R^2 = 0.988). Polymerisation conditions: benzene-d_6, 50 °C, $[L\text{-LA}]_0$ = 0.5 M.

3.4. Temperature variation

The effect of temperature was studied for *L*-lactide polymerisation carried out at 35−70 °C in benzene-d_6. As expected, the activity of **1** increased with increasing temperature (k_{obs} = 0.466 ± 0.053 h^{-1} (70 °C) > 0.055 ± 0.003 h^{-1} (35 °C)). Moderate polymerisation control was observed across the entire temperature range, as indicated by narrow-to-intermediate $Đ_M$ values (1.16−1.36). In addition, the $M_{n,SEC}$ and $M_{n,theo}$ of the polymers produced were in a good agreement. The Eyring plot of ln(k_{obs}/T) *vs.* 1/*T* was constructed to deduce the thermodynamic parameters for the ROP of *L*-lactide initiated by **1**. The corresponding $\Delta H^‡$, $\Delta S^‡$, and $\Delta G^‡$ values were calculated to be 50 ± 12 kJ mol^{-1}, −173 ± 36 J K^{-1} mol^{-1}, and 103−109 kJ mol^{-1} at 35−70 °C, respectively. These data are characteristic of a high-order transition state inherent in the coordination-insertion mechanism.[14] Comparable yet slightly lower thermodynamic parameters have been reported using other sterically constrained Lewis acidic metal systems: pyridine dipyrrolide bismuth *tert*-butoxide: $\Delta H^‡$ = 37 ± 1 kJ mol^{-1}; $\Delta S^‡$ = −162 ± 10 J K^{-1} mol^{-1}), an alkoxy-bridged dinuclear indium initiator ($\Delta H^‡$ = 49 ± 2 kJ mol^{-1}; $\Delta S^‡$ = −140 ± 12 J K^{-1} mol^{-1}), and amine-bridged *bis*(phenolato) neodymium alkoxide ($\Delta H^‡$ = 40 kJ mol^{-1}; $\Delta S^‡$ = −120 J K^{-1} mol^{-1}).[9, 15, 16]

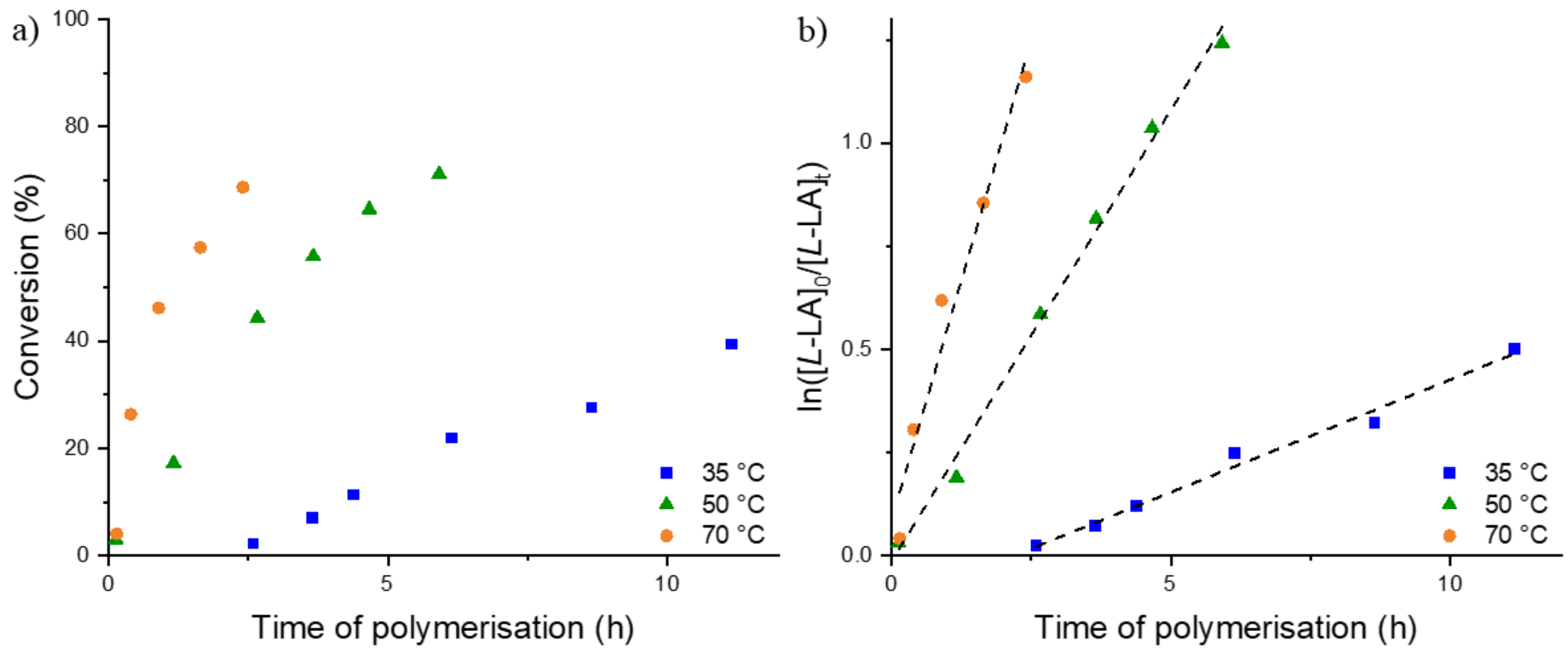

Figure S31: a) Plots of percentage conversion *vs*. polymerisation time for *L*-lactide polymerisation; b) Plots of ln([*L*-LA]$_0$/[*L*-LA]$_t$) *vs*. polymerisation time for *L*-lactide polymerisation. Blue squares: 35 °C, k_{obs} = 0.055 ± 0.003 h^{-1}, R^2 = 0.987; green up triangles: 50 °C, k_{obs} = 0.219 ± 0.008 h^{-1}, R^2 = 0.994; orange circles: 70 °C, k_{obs} = 0.466 ± 0.053 h^{-1}, R^2 = 0.962. Polymerisation conditions: benzene-d_6, [*L*-LA]$_0$/[Bi]$_0$ = 50:1, and [*L*-LA]$_0$ = 0.5 M.

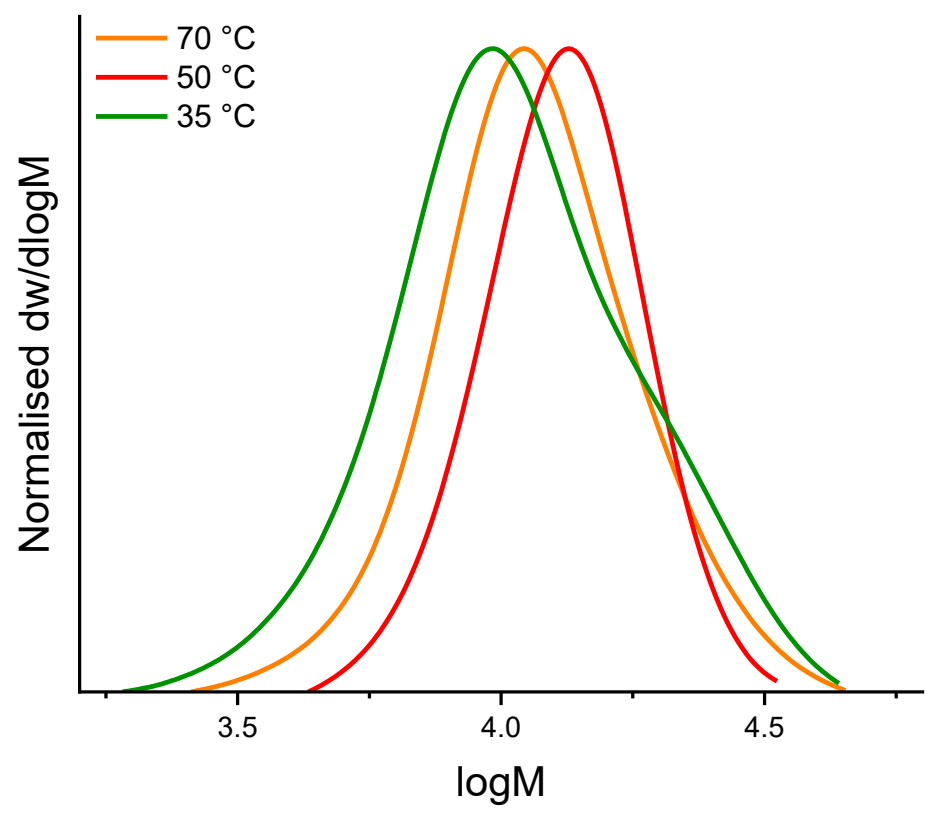

Figure S32: SEC plots of the PLLAs produced at 70 °C (orange, M_n = 5 960 g mol^{-1}, $Đ_M$ = 1.26); 50 °C (red, M_n = 5 210 g mol^{-1}, $Đ_M$ = 1.36); 35 °C (green, M_n = 6 910g mol^{-1}, $Đ_M$ = 1.16). Polymerisation conditions: benzene-d_6, [*L*-LA]$_0$/[Bi]$_0$ = 50:1, and [*L*-LA]$_0$ = 0.5 M.

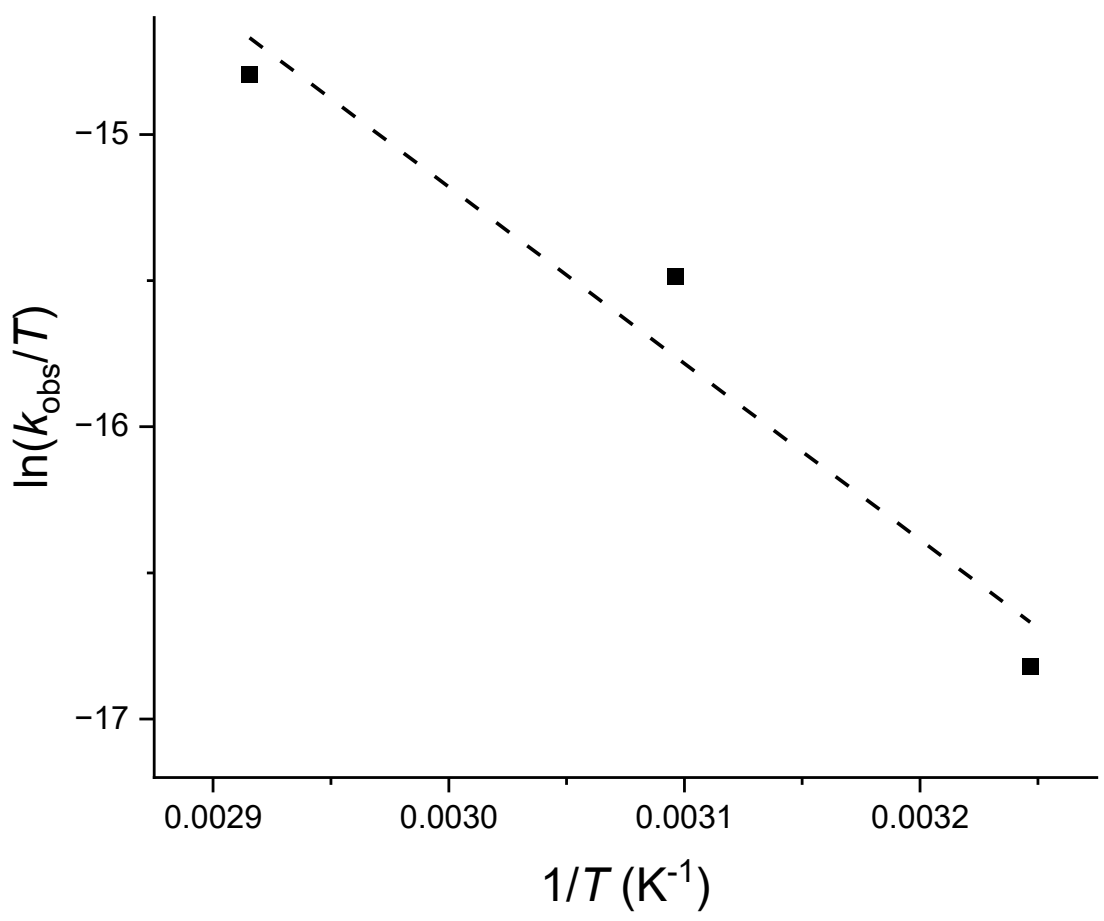

Figure S33: Eyring plot of ln(k_{obs}/T) *vs*. 1/T using **1** ($\Delta H^‡$ = 50 ± 12 kJ mol^{-1}; $\Delta S^‡$ = −173 ± 36 J K^{-1} mol^{-1}; slope = −6 036 ± 1 428, R^2 = 0.947; intercept = 2.9 ± 4.4). Polymerisation conditions: benzene-d_6, [*L*-LA]$_0$/[Bi]$_0$ = 50:1, and [*L*-LA]$_0$ = 0.5 M.

Table S3: ROP of *L*-lactide at different temperatures using **1**.[a]

T (°C)	**Conv.[b]** (%)	***k*****obs**[c] (h^{-1})	***R*****[2c]**	***M*****n,SEC**[d] (g mol^{-1})	***M*****n,theo**[e] (g mol^{-1})	***M*****w/*M*****n**[d]
35	94	0.055 ± 0.003	0.987	6 910	6 819	1.16
50	78	0.219 ± 0.008	0.994	5 210	5 378	1.36
70	90	0.466 ± 0.053	0.962	5 960	6 531	1.26

[a]Polymerisation conditions: benzene-d_6, $[L\text{-LA}]_0/[\text{Bi}]_0$ = 50:1, $[L\text{-LA}]_0$ = 0.5 M. [b]Measured by ^{1}H NMR spectroscopy. [c]Obtained from plots of $\ln([L\text{-LA}]_0/[L\text{-LA}]_t)$ *vs.* polymerisation time. [d]Determined by SEC analysis calibrated with PS standards in THF with the appropriate Mark-Houwink corrections.[3] [e]Theoretical M_n for PLA = (conv.(%) × $[L\text{-LA}]_0/[\text{Bi}]_0$) × 144.13 + ($M_w$ of end groups).

3.5. Addition of 1 equiv. *L*-LA to compound 2

One equiv. *L*-LA was added to a benzene-d_6 solution of **2** and the reaction mixture was monitored by ^{1}H NMR spectroscopy. No reactivity was observed at room temperature after 24 hours. After heating at 50°C for 2 hours, the formation of small quantities of oligomeric PLLA was observed alongside unreacted **2**.

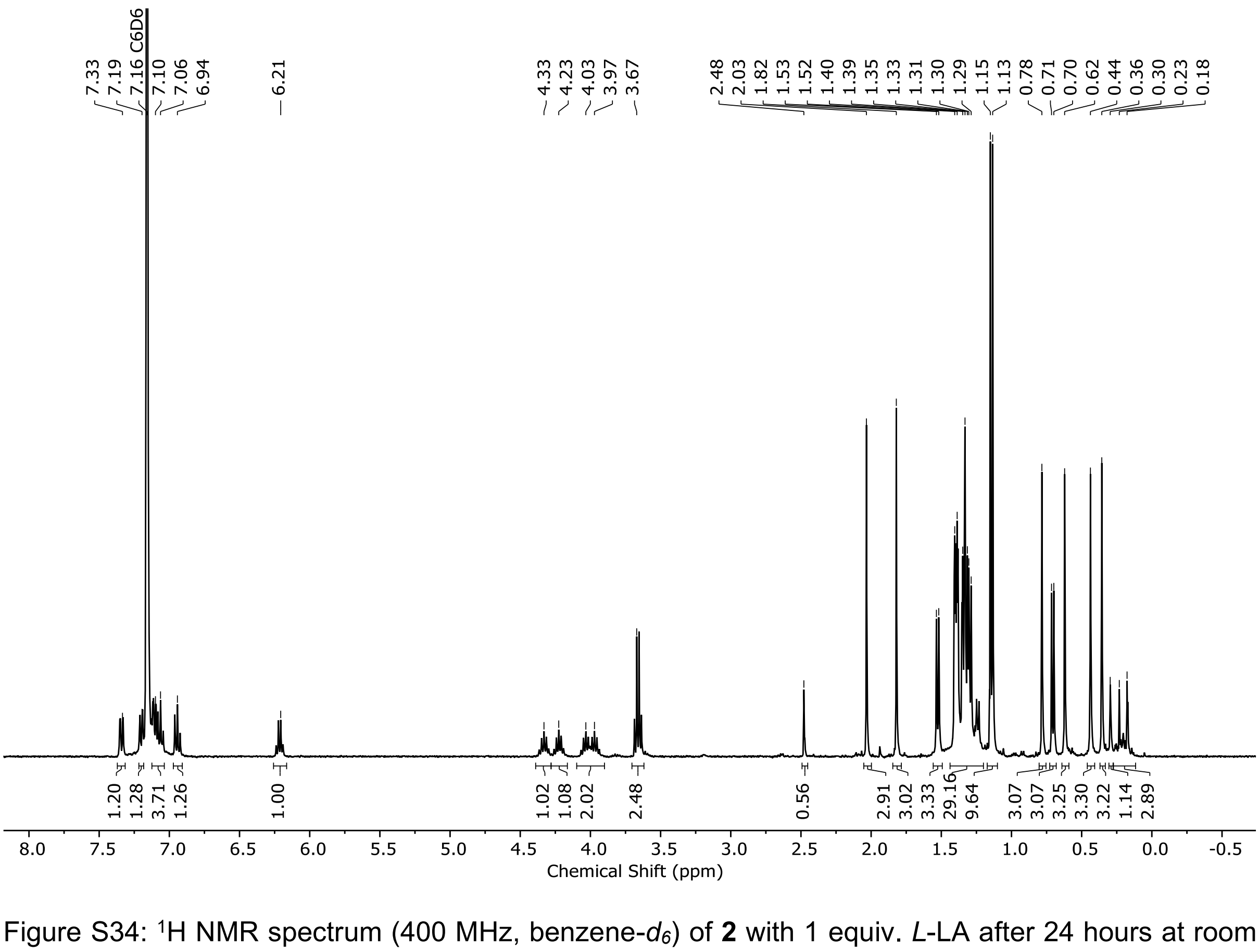

Figure S34: ^{1}H NMR spectrum (400 MHz, benzene-d_6) of **2** with 1 equiv. *L*-LA after 24 hours at room temperature, showing unreacted starting material only.

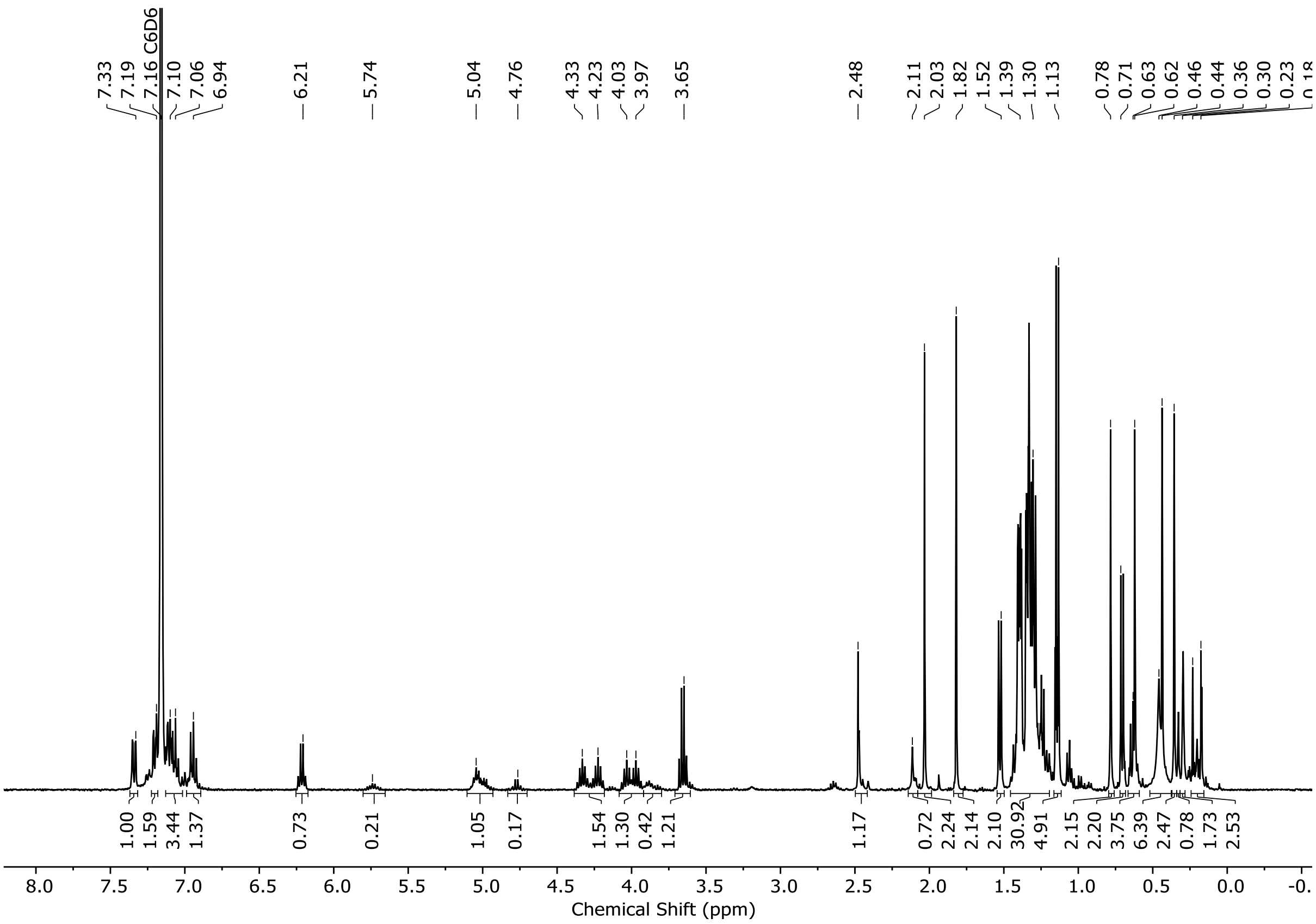

Figure S35: ^{1}H NMR spectrum (400 MHz, benzene-d_6) of **2** with 1 equiv. *L*-LA after heating at 50°C for 2 hours.

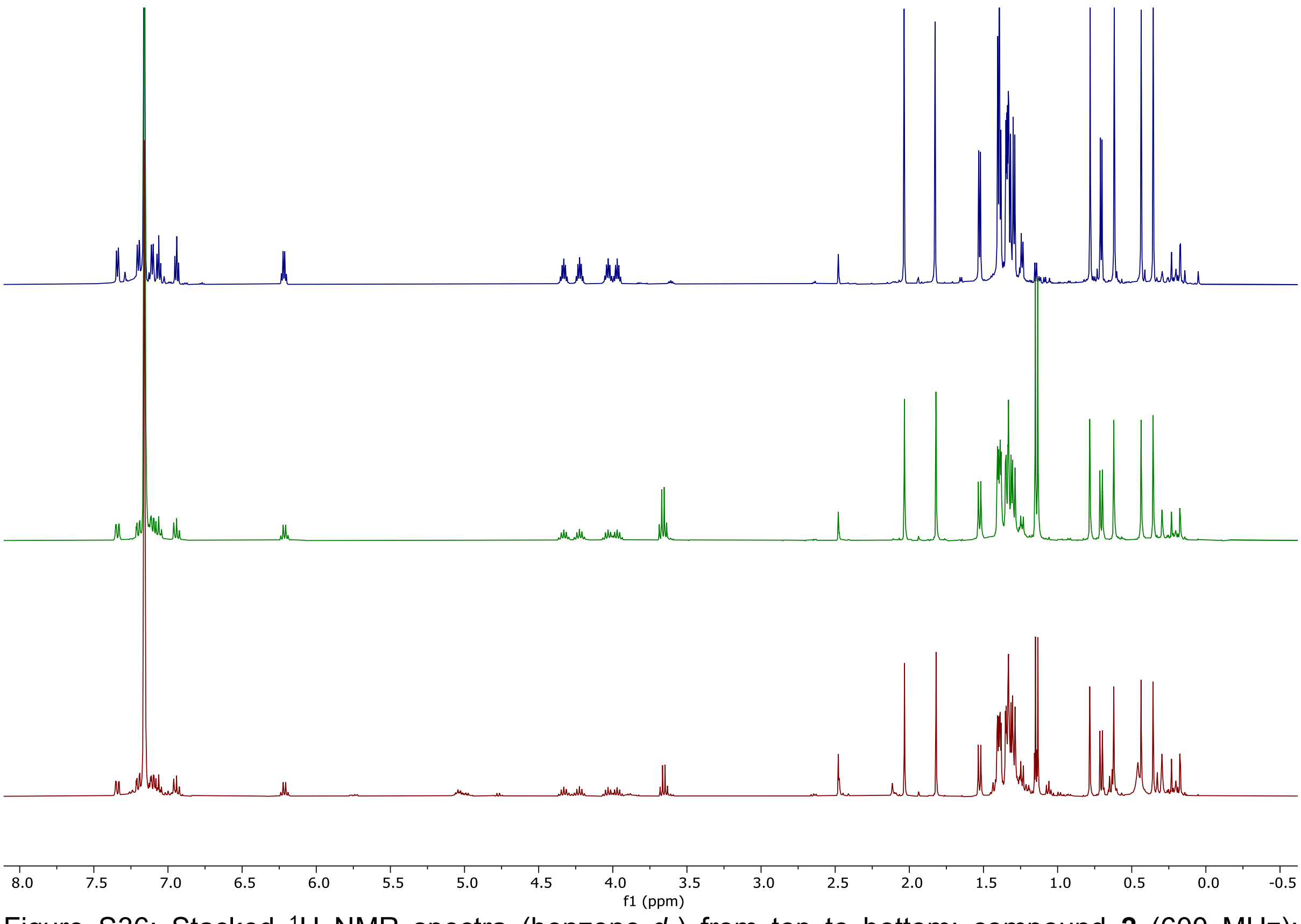

Figure S36: Stacked ^{1}H NMR spectra (benzene-d_6) from top to bottom: compound **2** (600 MHz); compound **2** with 1 equiv. *L*-LA after 24 hours at room temperature (400 MHz); compound **2** with 1 equiv. *L*-LA after 2 hours at 50°C (400 MHz).

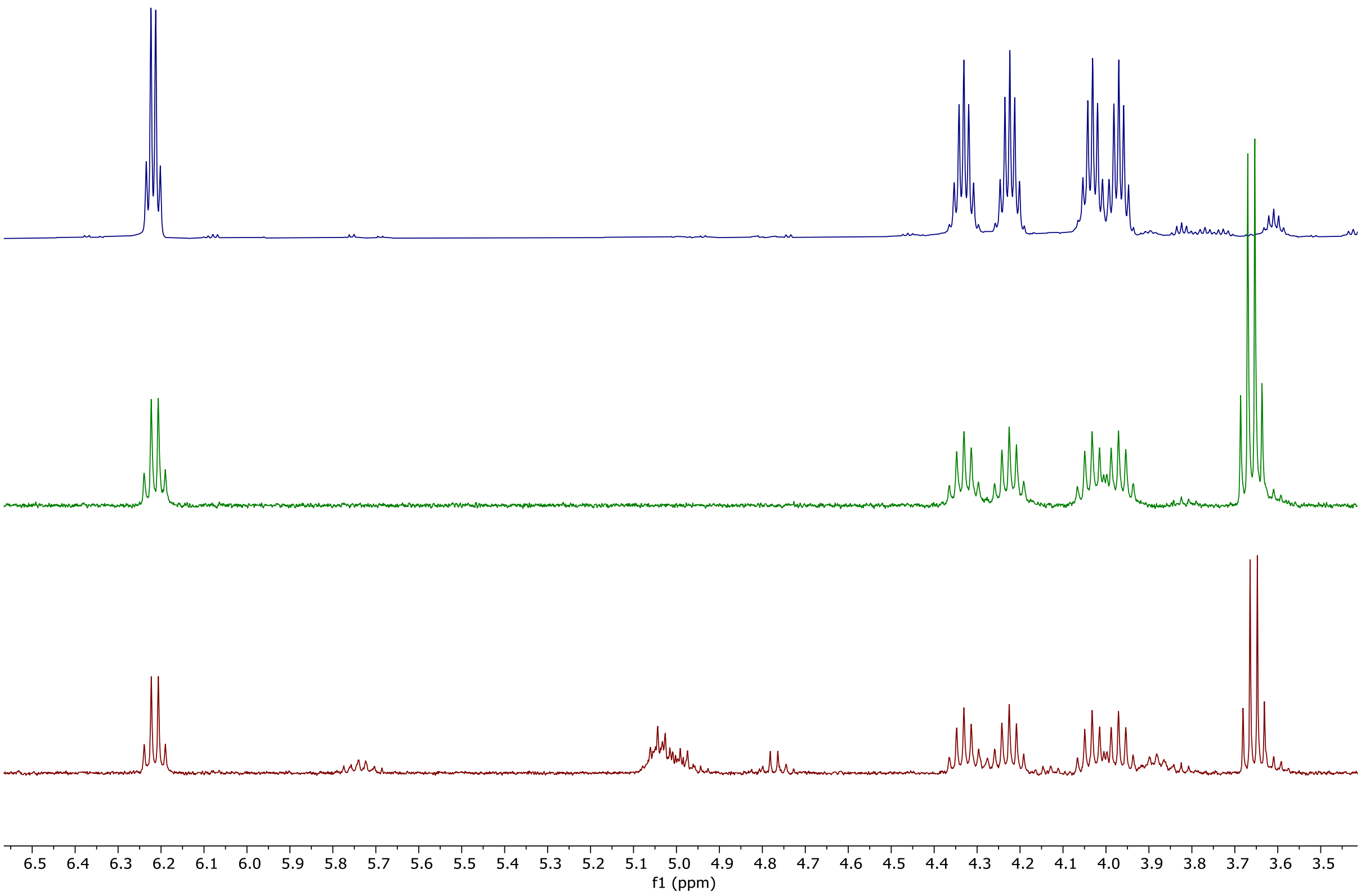

Figure S37: Expanded stacked ^1H NMR spectra (benzene-d_6) from top to bottom: compound **2** (600 MHz); compound **2** with 1 equiv. *L*-LA after 24 hours at room temperature (400 MHz); compound **2** with 1 equiv. *L*-LA after 2 hours at 50°C (400 MHz).

3.6. Sequential addition of increasing equivalents of *L*-LA to 1

A 0.2 M stock solution of *L*-LA in benzene-d_6 was prepared, and 25 µl of this solution (0.005 mmol *L*-LA) was added to a 0.5 ml benzene-d_6 solution of compound **1** (7.3 mg, 0.01 mmol) in a J. Young's NMR tube. Analysis of the reaction mixture by ^1H NMR spectroscopy after 16 hours at 25°C confirmed formation of compound **2**. Portions of the stock solution were added sequentially, and the solution analysed by ^1H NMR spectroscopy after each addition, such that the total amount of *L*-LA added was 0.025 mmol (2.5 equiv.), 0.050 mmol (5 equiv.), 0.100 mmol (10 equiv.). After that, 14.4 mg *L*-LA (0.100 mmol) was added as a solid, and the solution analysed by ^1H NMR spectroscopy (total 0.20 mmol, 20 equiv. *L*-LA). Then a further 43.2 mg *L*-LA (0.300 mmol) was added (total 0.50 mmol, 50 equiv.). The solution was again analysed by ^1H NMR spectroscopy, first at 298 K, then at 323 K. The chemical shift of the resonances corresponding to **2** were not observed to change significantly with increasing amounts of added *L*-LA, but were found to change significantly depending on temperature.

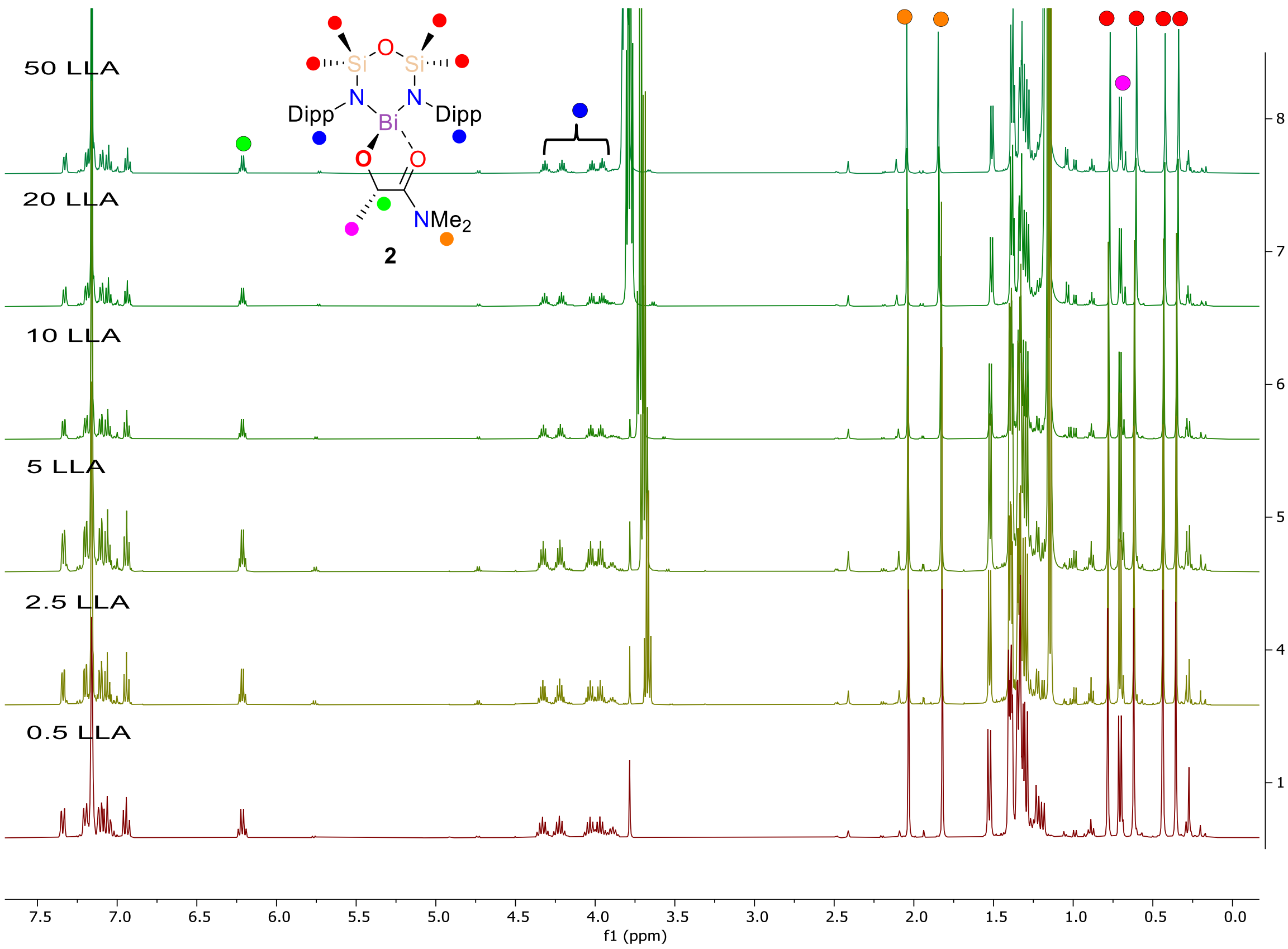

Figure S38: Stacked ^{1}H NMR spectra (500 MHz, benzene-d_6, 298 K) obtained after the sequential addition of increasing amounts of *L*-LA to compound **1** at 298 K, showing formation of compound **2**.

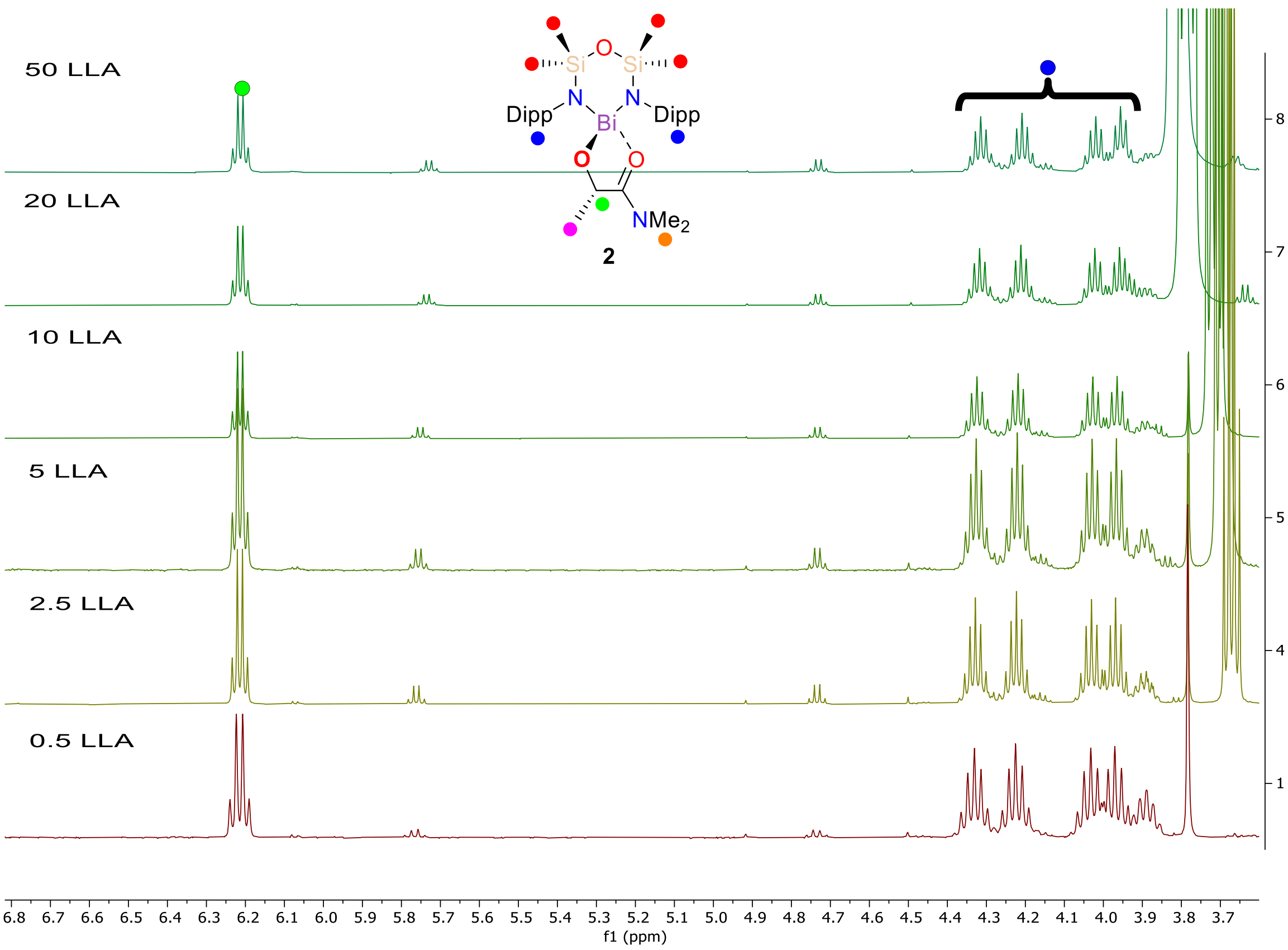

Figure S39: Selected region of the stacked ^{1}H NMR spectra (500 MHz, benzene-d_6, 298 K) obtained after the sequential addition of increasing amounts of *L*-LA to compound **1** at 298 K, showing

formation of compound **2**. Resonances at 5.76 and 4.72 ppm correspond to the terminal C*H*(Me) proton of oligomeric PLLA.

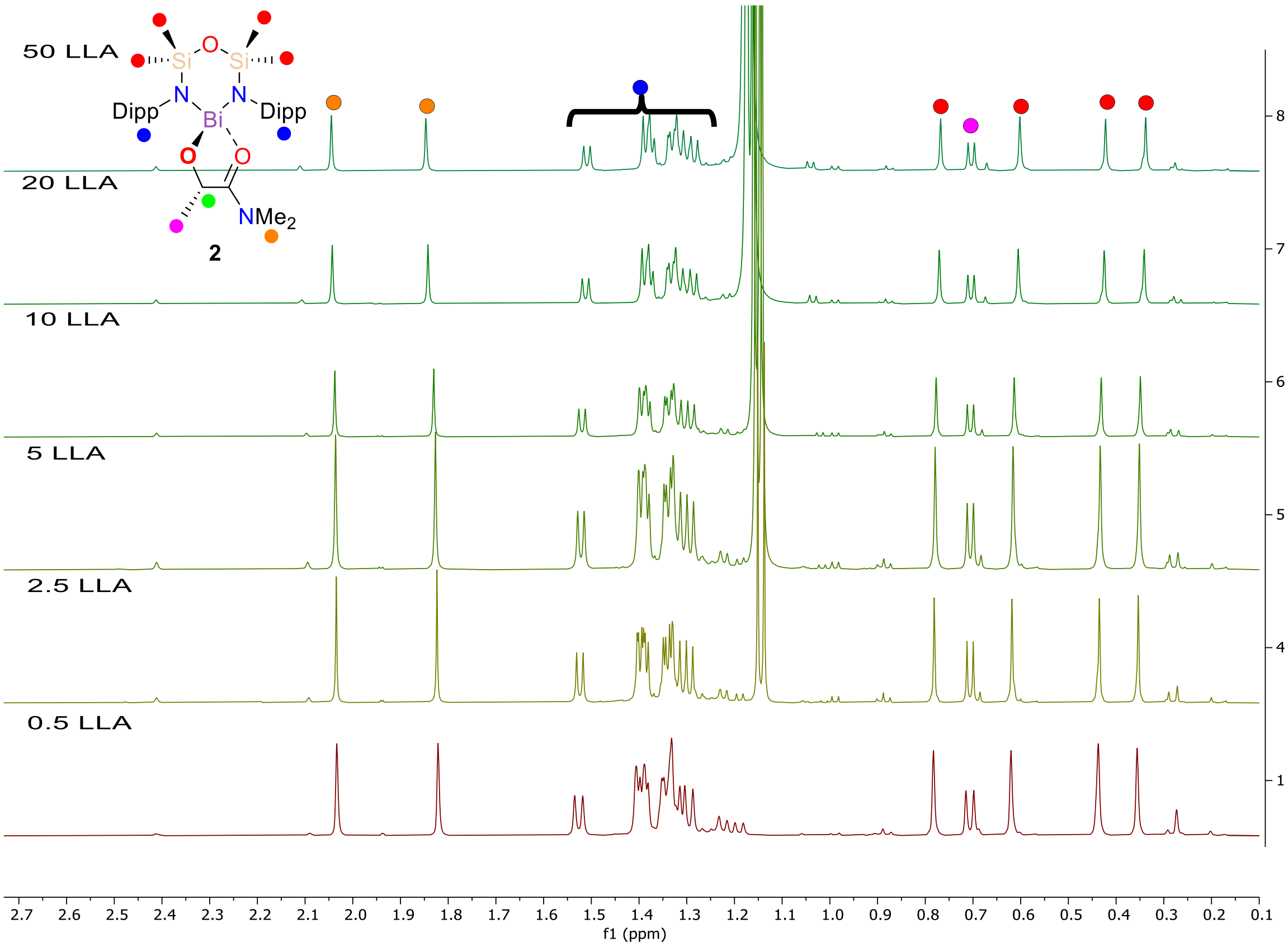

Figure S40: Selected region of the stacked ^{1}H NMR spectra (500 MHz, benzene-d_6, 298 K) obtained after the sequential addition of increasing amounts of *L*-LA to compound **1** at 298 K, showing formation of compound **2**.

3.7. In situ NMR monitoring for the reaction of 1 with *L*-LA

Analysis of the polymerisation of *L*-LA with **1** at 50°C by *in situ* ^{1}H NMR spectroscopy after 10 minutes showed the formation of trace amounts of oligomeric PLLA and ligand resonances due to the the bismuth-bound propagating chain, in addition to compound **2**, whose resonances were identified by comparison to the ^{1}H NMR spectrum of the compound without additional *L*-LA at 323 K. Analysis after 4.5 hours showed transformation into a (NON^{dipp})Bi-ligated ω-NMe_2 capped propagating polymer chain.

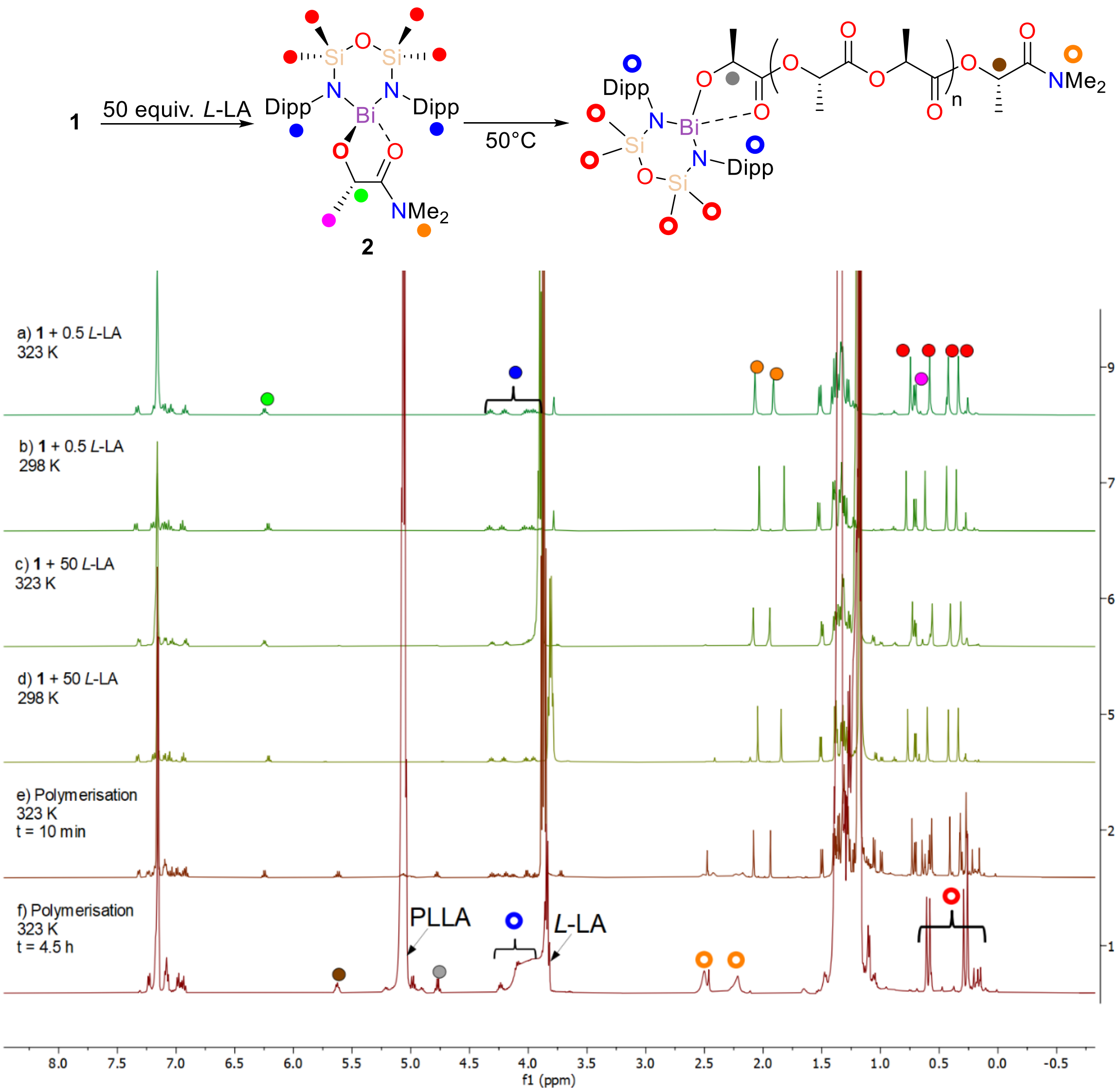

Figure S41: Stacked ^{1}H NMR spectra (benzene-d_6, 400 MHz): a) compound **2** formed in situ from room temperature reaction of **1** + 0.5 *L*-LA (323 K); b) the same sample at 298 K; c) sample obtained after room temperature reaction of 50 equiv. *L*-LA with **1** (323 K); d) the same sample at 298 K; e) L-lactide polymerisation using **1** at t = 10 minutes; f) L-lactide polymerisation using **1** at t = 4.7 hours. Polymerisation conditions: benzene-d_6, 50 °C, $[L\text{-}LA]_0/[Bi]_0 = 50$, and $[L\text{-}LA]_0 = 0.5$ M.

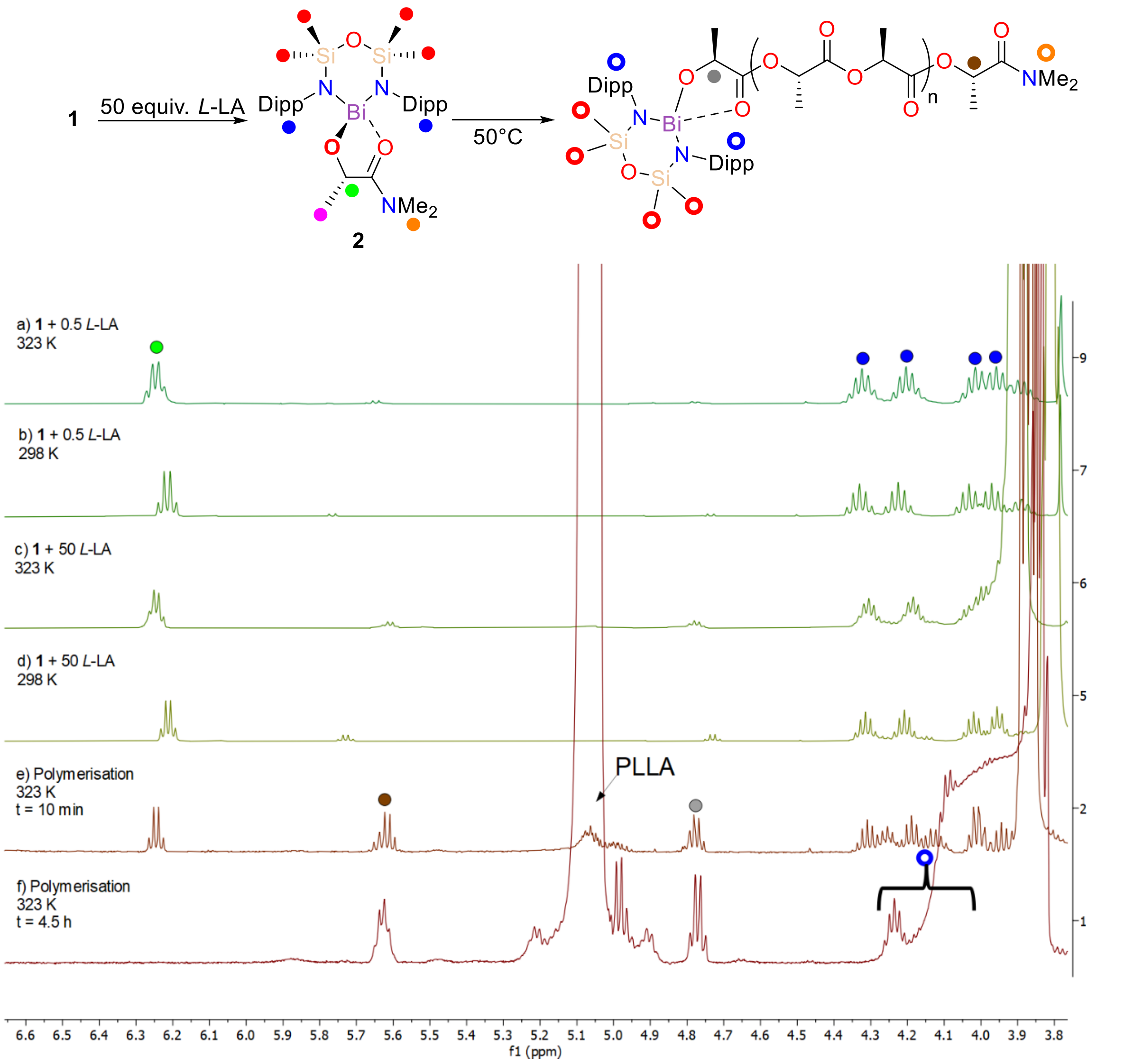

Figure S42: Selected region of the stacked ^{1}H NMR spectra (benzene-*d*$_6$, 400 MHz): a) compound **2** formed in situ from room temperature reaction of **1** + 0.5 *L*-LA (323 K); b) the same sample at 298 K; c) sample obtained after room temperature reaction of 50 equiv. *L*-LA with **1** (323 K); d) the same sample at 298 K; e) L-lactide polymerisation using **1** at t = 10 minutes; f) L-lactide polymerisation using **1** at t = 4.7 hours. Polymerisation conditions: benzene-*d*$_6$, 50 °C, $[L\text{-}LA]_0/[Bi]_0 = 50$, and $[L\text{-}LA]_0 = 0.5$ M.

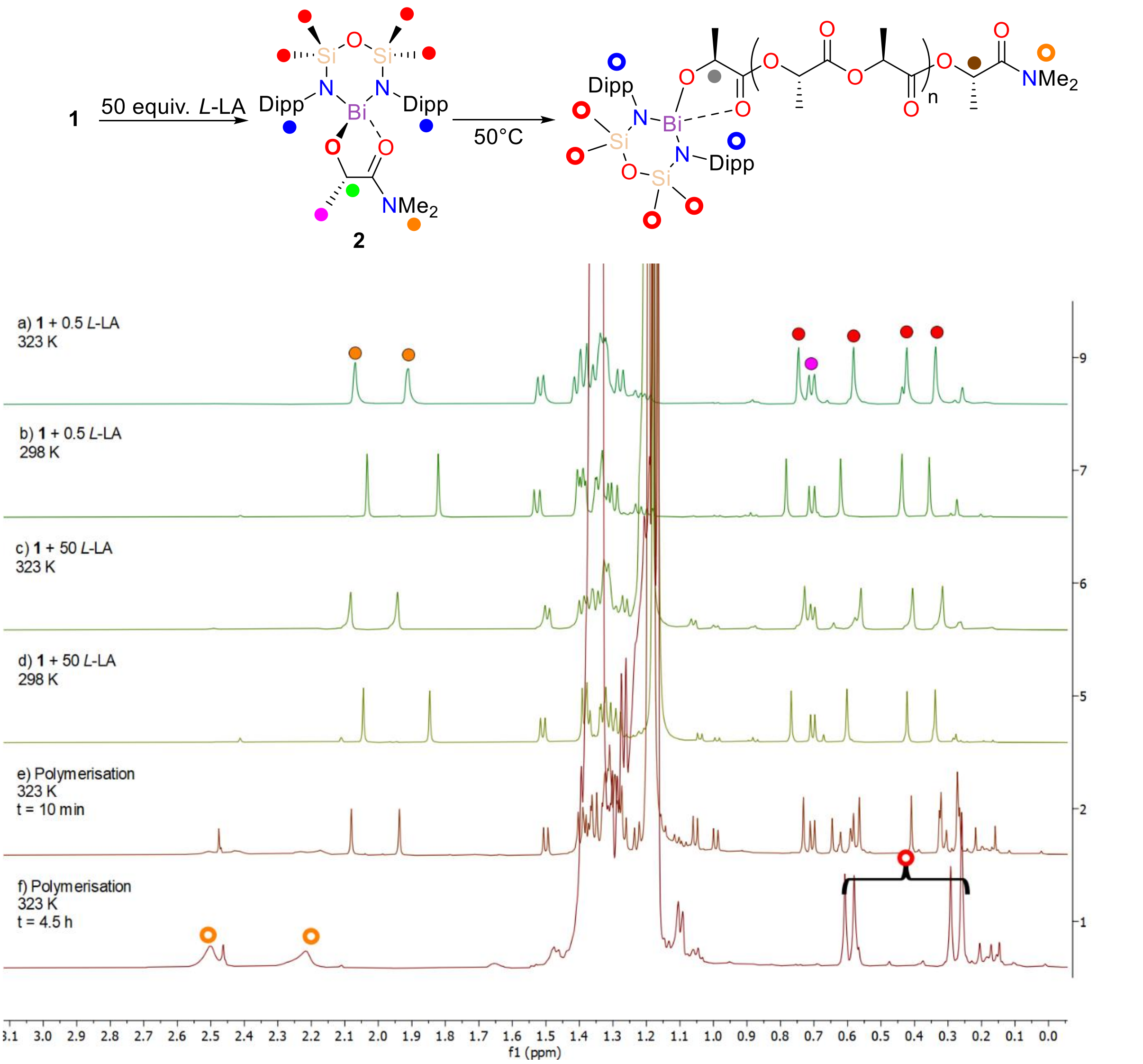

Figure S43: Selected region of the stacked ^{1}H NMR spectra (benzene-d_6, 400 MHz): a) compound **2** formed in situ from room temperature reaction of **1** + 0.5 *L*-LA (323 K); b) the same sample at 298 K; c) sample obtained after room temperature reaction of 50 equiv. *L*-LA with **1** (323 K); d) the same sample at 298 K; e) L-lactide polymerisation using **1** at t = 10 minutes; f) L-lactide polymerisation using **1** at t = 4.7 hours. Polymerisation conditions: benzene-d_6, 50 °C, $[L\text{-}LA]_0/[Bi]_0 = 50$, and $[L\text{-}LA]_0 = 0.5$ M.

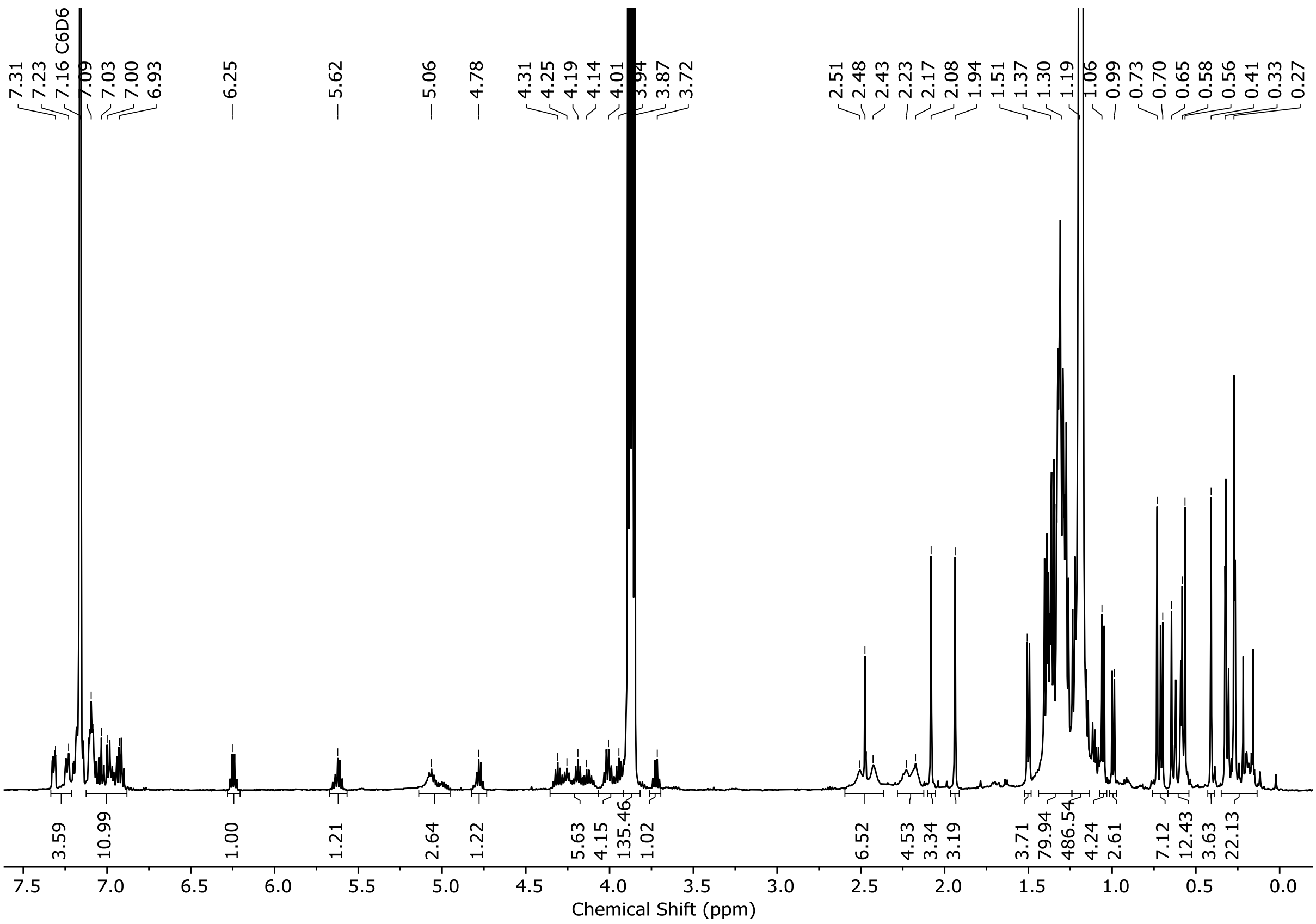

Figure S44: ^{1}H NMR spectrum (benzene-d_6, 400 MHz) of L-lactide polymerisation using **1** at t = 10 minutes. Polymerisation conditions: benzene-d_6, 50 °C, $[\text{L-LA}]_0/[\text{Bi}]_0 = 50$, and $[\text{L-LA}]_0 = 0.5$ M.

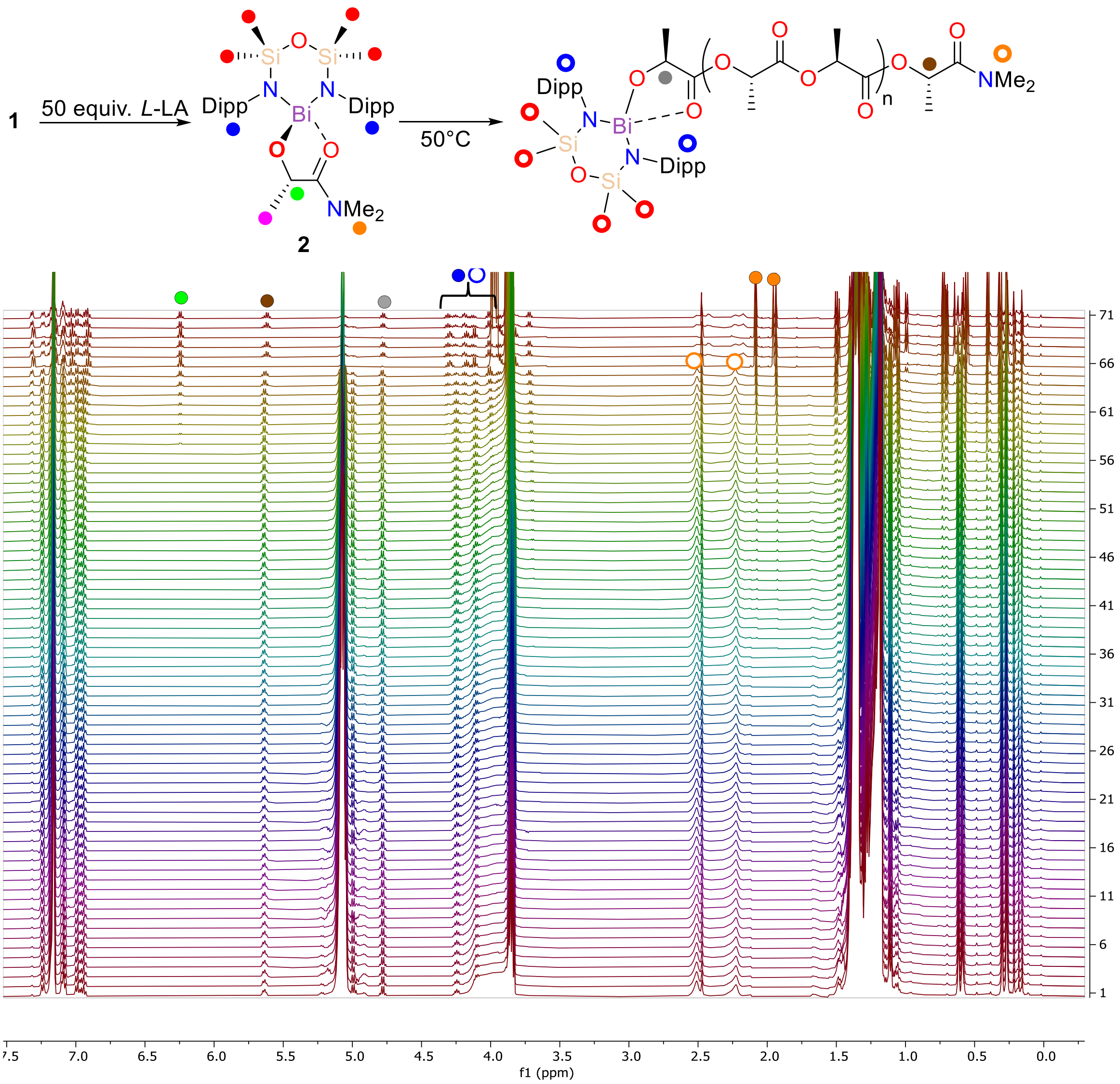

Figure S45: Stacked ^{1}H NMR spectra (benzene-d_6, 400 MHz) of representative L-lactide polymerisation *vs.* time from 10 minutes (top) to 4.7 hours (bottom), highlighting consumption of **2** and evolution of propagating polymer chain.

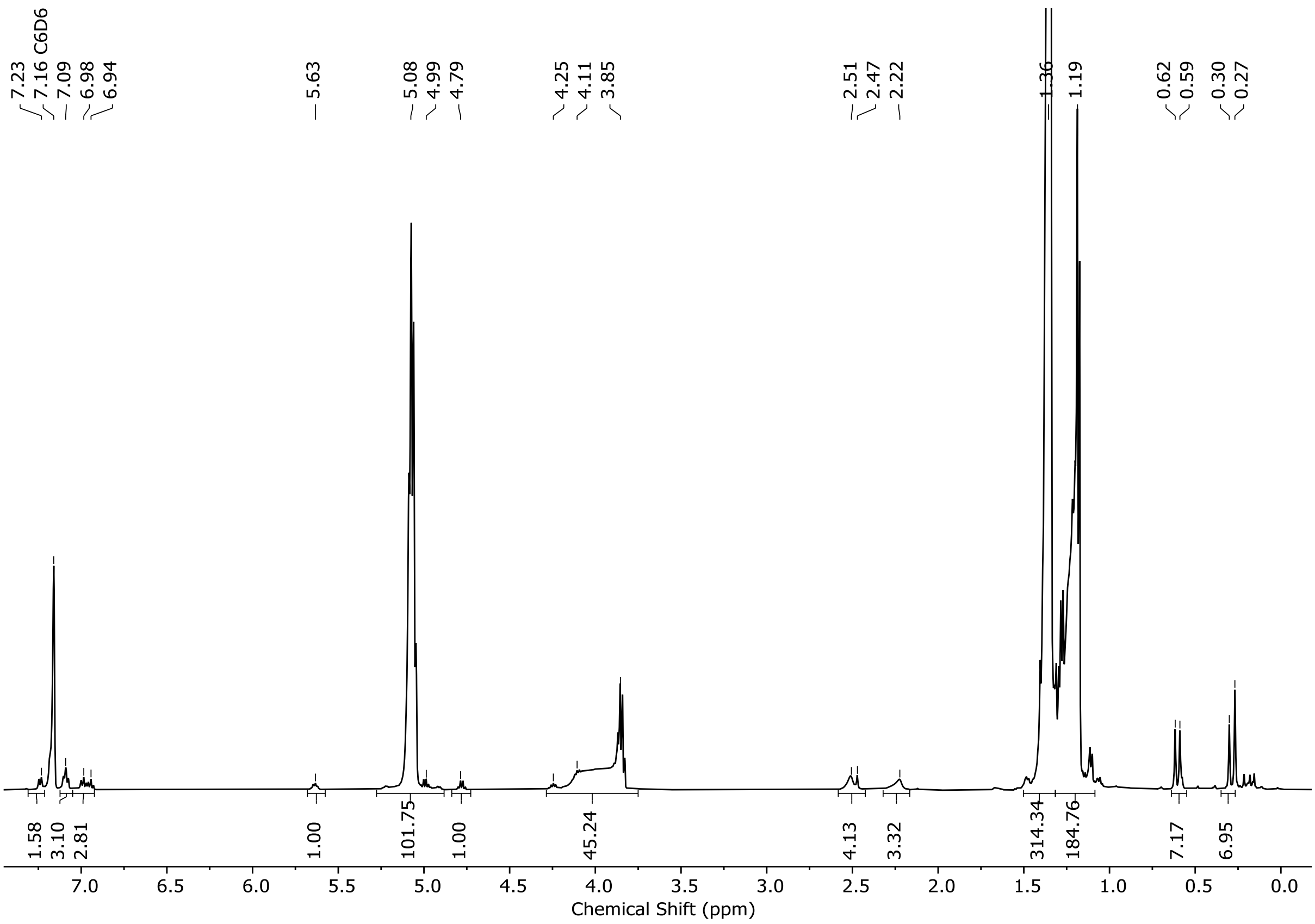

Figure S46: ^{1}H NMR spectrum (benzene-d_6, 400 MHz) of L-lactide polymerisation using **1** at t = 4.7 hours. Polymerisation conditions: benzene-d_6, 50 °C, $[L\text{-}LA]_0/[Bi]_0 = 50$, and $[L\text{-}LA]_0 = 0.5$ M.

3.8. Lactide polymerisation studies using bismuth amidolactate 2

Both **1** and **2** exhibited similar activities ($k_{obs} = 0.219 \pm 0.008$ and 0.191 ± 0.007 h^{-1}, respectively). Furthermore, the polymer afforded by **2** showed a good agreement between its $M_{n,SEC}$ and $M_{n,theo}$, in conjunction with a relatively narrow $Đ_M$ of 1.20, hence implying good polymerisation control.

The initial ^{1}H NMR spectra (*L*-lactide conversion < 3%) were essentially identical for both **1** and **2** as initiator. Comparing the initial *in situ* NMR spectrum of each of these polymerisation runs with the NMR spectra of compound **2** at room temperature and at 50 °C with and without added 50 equiv. *L*-LA confirmed that compound **2** is initially formed in polymerisation runs initiated by **1**. Thus, after initial ring-opening mediated by **1** and transesterification to generate **2**, compound **2** is likely to represent the genuine polymerisation initiator prior to propagation.

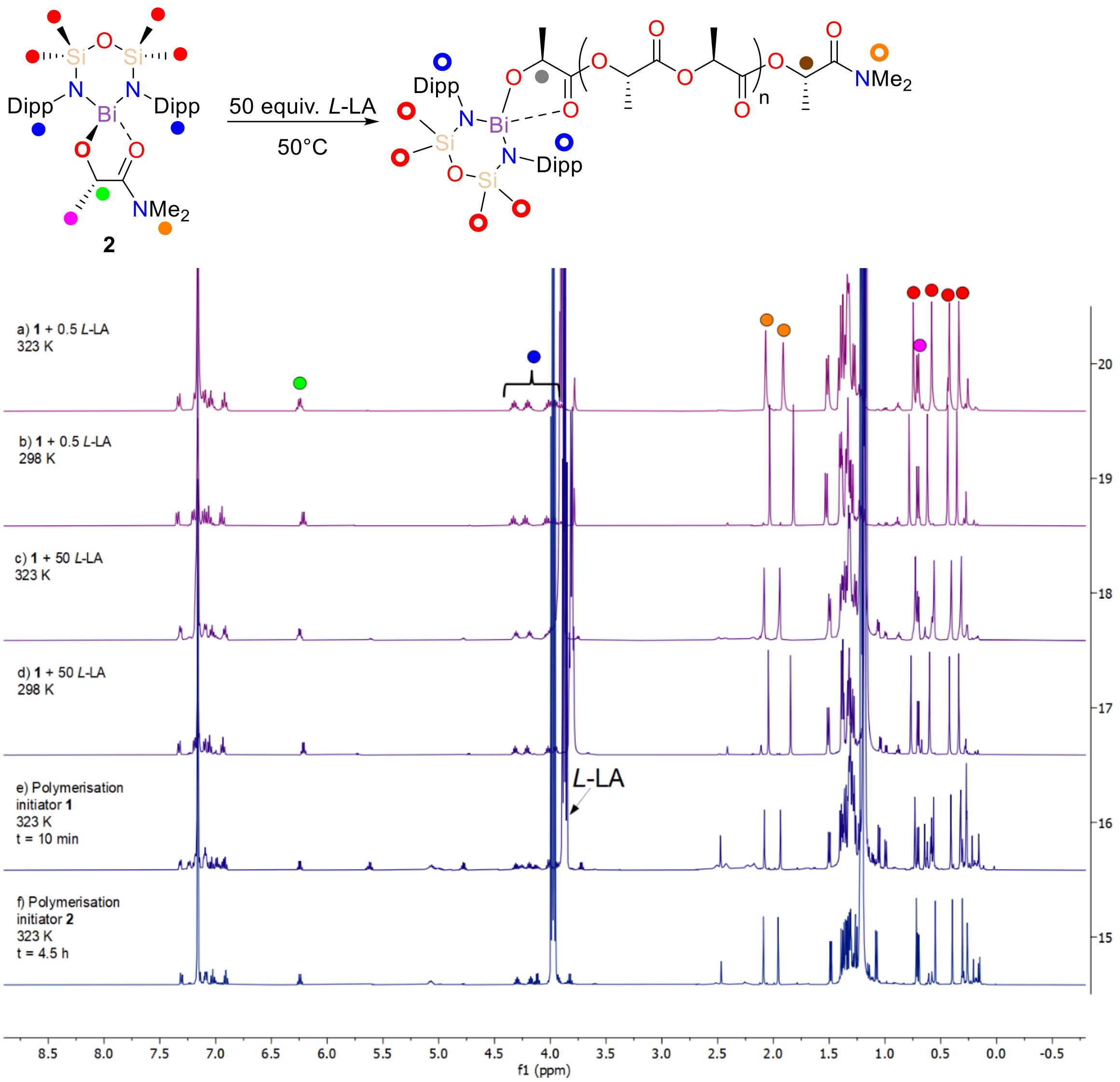

Figure S47: Stacked ^{1}H NMR spectra (benzene-d_6, 400 MHz): a) compound **2** formed in situ from room temperature reaction of **1** + 0.5 *L*-LA (323 K); b) the same sample at 298 K; c) sample obtained after room temperature reaction of 50 equiv. *L*-LA with **1** (323 K); d) the same sample at 298 K; e) L-lactide polymerisation using **1** at t = 10 minutes; f) L-lactide polymerisation using **2** at t = 10 minutes. Polymerisation conditions: benzene-d_6, 50 °C, $[L\text{-}LA]_0/[Bi]_0 = 50$, and $[L\text{-}LA]_0 = 0.5$ M. This highlights that compound **2** is the initially formed propagating species when **1** is used as initiator.

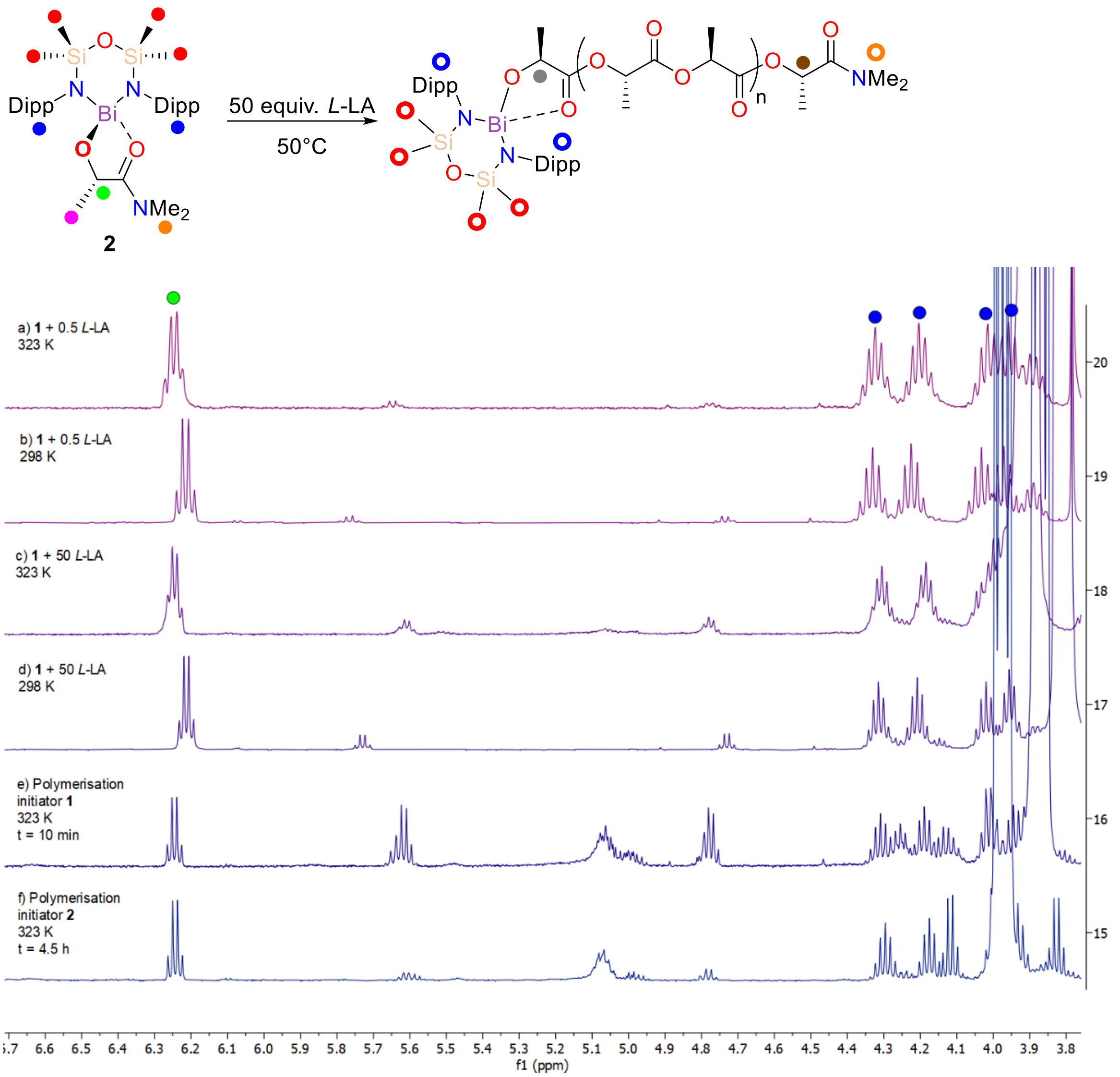

Figure S48: Selected region of the stacked ^{1}H NMR spectra (benzene-d_6, 400 MHz): a) compound **2** formed in situ from room temperature reaction of **1** + 0.5 *L*-LA (323 K); b) the same sample at 298 K; c) sample obtained after room temperature reaction of 50 equiv. *L*-LA with **1** (323 K); d) the same sample at 298 K; e) L-lactide polymerisation using **1** at t = 10 minutes; f) L-lactide polymerisation using **2** at t = 10 minutes. Polymerisation conditions: benzene-d_6, 50 °C, $[L\text{-}LA]_0/[Bi]_0 = 50$, and $[L\text{-}LA]_0 = 0.5$ M. This highlights that compound **2** is the initially formed propagating species when **1** is used as initiator.

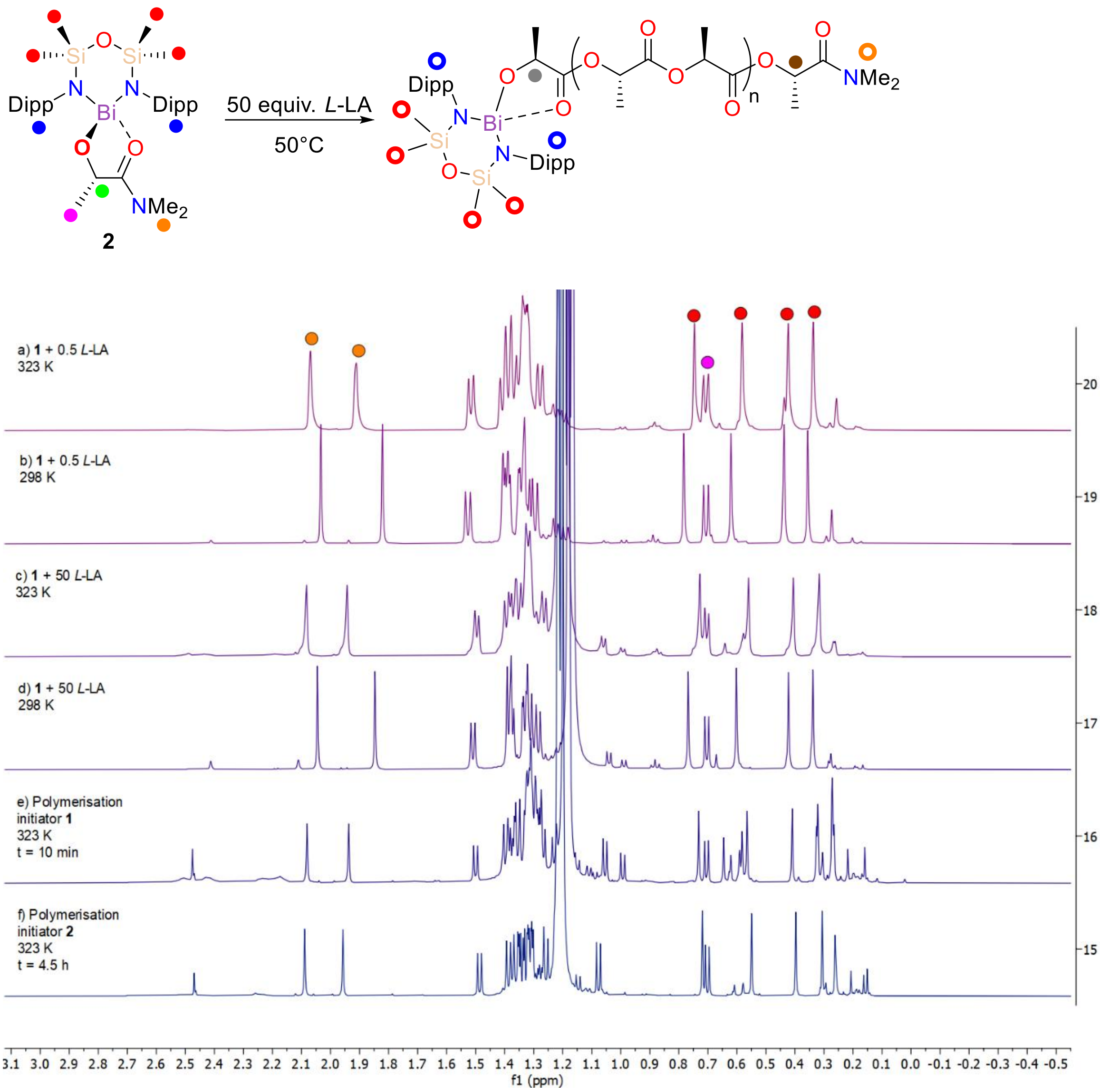

Figure S49: Stacked ^{1}H NMR spectra (benzene-d_6, 400 MHz): a) compound **2** formed in situ from room temperature reaction of **1** + 0.5 *L*-LA (323 K); b) the same sample at 298 K; c) sample obtained after room temperature reaction of 50 equiv. *L*-LA with **1** (323 K); d) the same sample at 298 K; e) L-lactide polymerisation using **1** at t = 10 minutes; f) L-lactide polymerisation using **2** at t = 10 minutes. Polymerisation conditions: benzene-d_6, 50 °C, $[\text{L-LA}]_0/[\text{Bi}]_0 = 50$, and $[\text{L-LA}]_0 = 0.5$ M. This highlights that compound **2** is the initially formed propagating species when **1** is used as initiator.

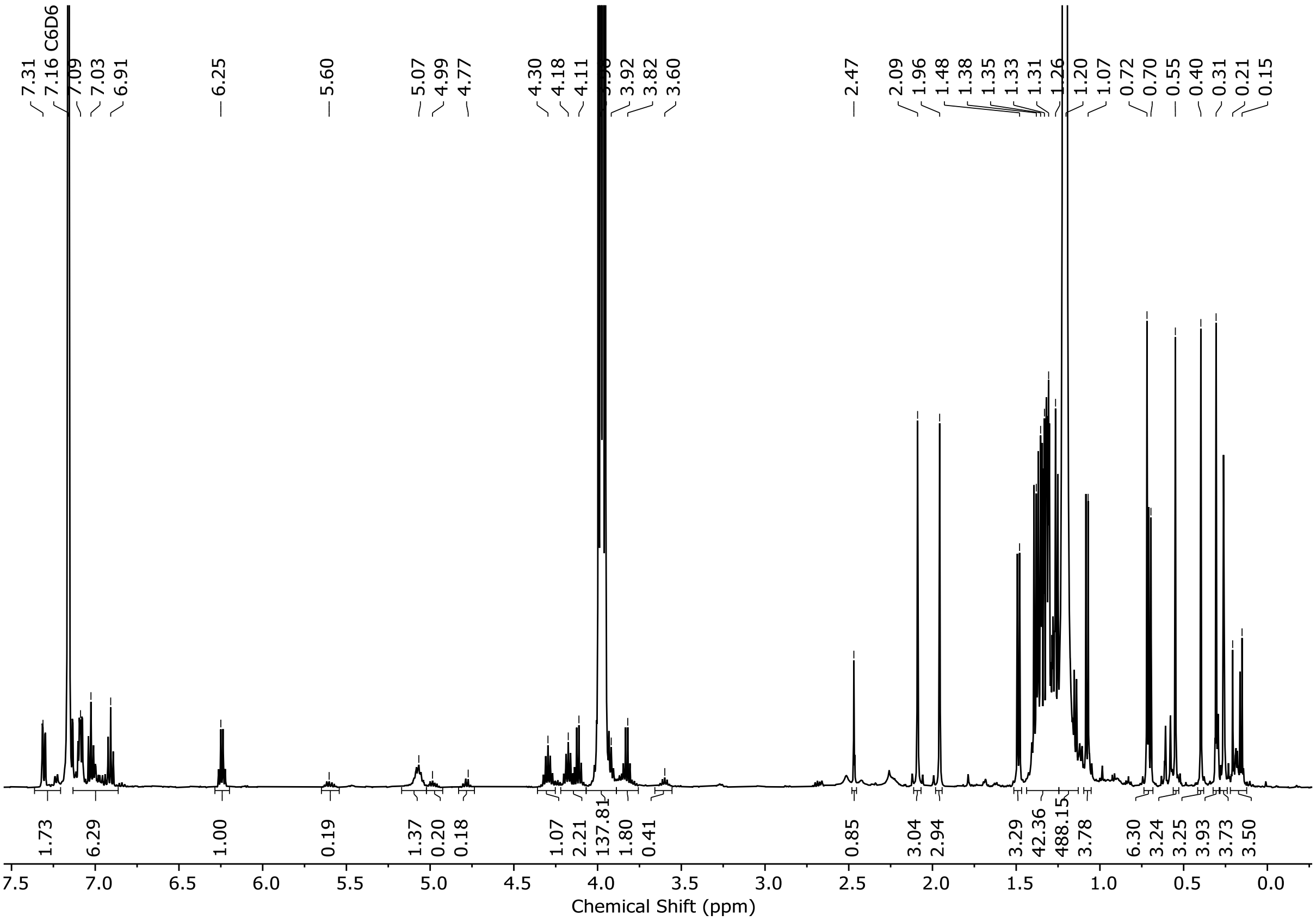

Figure S50: ^{1}H NMR spectrum (benzene-d_6, 400 MHz) of L-lactide polymerisation using **2** at t = 10 minutes. Polymerisation conditions: benzene-d_6, 50 °C, $[\text{L-LA}]_0/[\text{Bi}]_0$ = 50, and $[\text{L-LA}]_0$ = 0.5 M.

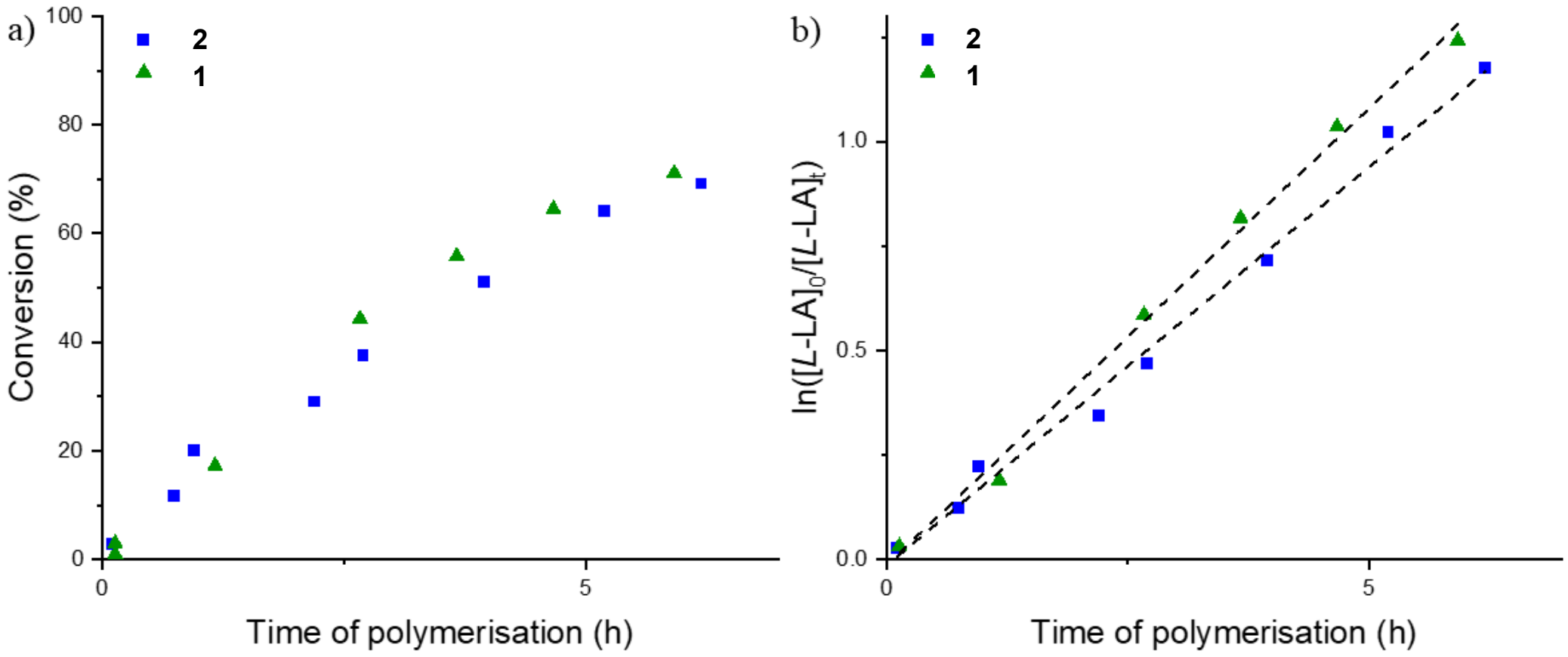

Figure S51: a) Plots of percentage conversion *vs*. polymerisation time for *L*-lactide polymerisation; b) plots of $\ln([L\text{-LA}]_0/[L\text{-LA}]_t)$ *vs*. polymerisation time for *L*-lactide polymerisation. Blue squares: **2**, k_{obs} = 0.191 ± 0.007 h^{-1}, R^2 = 0.991; green up triangles: **1**, k_{obs} = 0.219 ± 0.008 h^{-1}, R^2 = 0.994. Polymerisation conditions: benzene-d_6, 50 °C, $[L\text{-LA}]_0/[\text{Bi}]_0$ = 50:1, and $[L\text{-LA}]_0$ = 0.5 M.

Table S4: ROP of *L*-lactide using **2**.[a]

**Conv.**[b] (%)	k_{obs}[c] (h^{-1})	R^{2c}	$M_{n,SEC}$[d] (g mol^{-1})	$M_{n,theo}$[e] (g mol^{-1})	M_w/M_n[d]
92	0.191 ± 0.007	0.991	6 900	6 800	1.20

[a]Polymerisation conditions: benzene-d_6, 50 °C, $[L\text{-LA}]_0/[Bi]_0$ = 50:1, $[L\text{-LA}]_0$ = 0.5 M. [b]Measured by ^1H NMR spectroscopy. [c]Obtained from plots of $\ln([L\text{-LA}]_0/[L\text{-LA}]_t)$ *vs.* polymerisation time. [d]Determined by SEC analysis calibrated with PS standards in THF with the appropriate Mark-Houwink corrections.[3] [e]Theoretical M_n for PLA = (conv.(%) × $[L\text{-LA}]_0/[Bi]_0$) × 144.13 + (M_w of end groups).

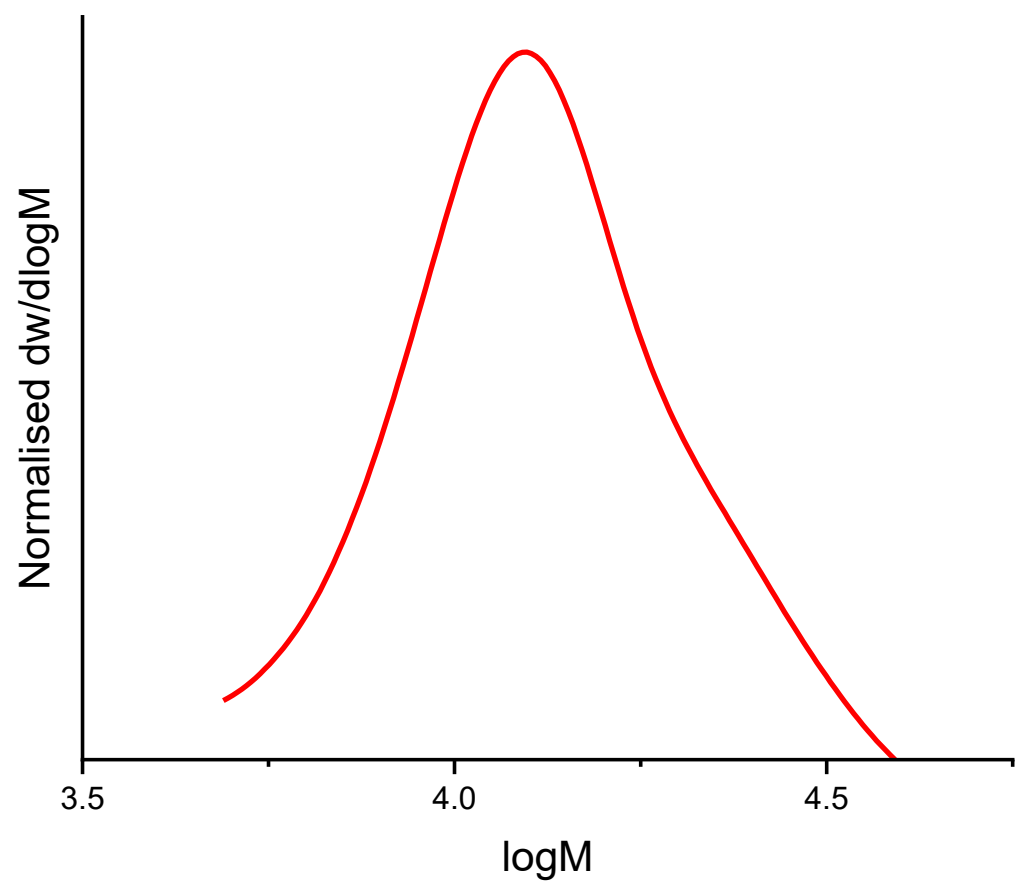

Figure S52: SEC plot of the PLA produced using **2** (M_n = 6 900 g mol^{-1}, $Đ_M$ = 1.20). Polymerisation conditions: benzene-d_6, 50 °C, $[L\text{-LA}]_0/[Bi]_0$ = 50:1, and $[L\text{-LA}]_0$ = 0.5 M.

3.9. End-group analysis of PLLA

A PLLA polymer sample afforded by **1** was analysed by MALDI-ToF mass spectrometry to determine the nature of polymer end groups. The mass spectrum displayed a series of peaks separated by *m*/*z* = 72, indicative of intermolecular transesterification taking place during the polymerisation. The major series of peaks was assigned to the PLLA chain terminated by α–NMe_2 and ω–OH end groups, consistent with ROP proceeding *via* a coordination-insertion mechanism. This can be illustrated by the peak at 2 966.1 g mol^{-1} corresponding to the α–NMe_2/ω–OH end-capped polymer chain comprising 20 lactide units together with K^+ (M_n = (144.1 × 20 + 39.1 + 45.1) g mol^{-1}). An additional series of peaks at the lower molecular weight range was attributed to the polymer chain without end groups, indicating the formation of macrocyclic PLLA *via* intramolecular transesterification.

The presence of the α–NMe_2 end group was also observed in the ^1H NMR spectrum of the isolated PLLA, with two characteristic proton singlets located at δ 2.45 and 2.09 ppm. Additionally, separate multiplet resonances were observed for chain-end C*H*Me *methine* and CH*Me methyl* groups centred at δ 4.1, 3.9, and 1.1 ppm. The tandem formation of both linear and cyclic PLLA was also evidenced by ^1H NMR spectroscopic analysis. Deconvolution of ^1H NMR spectra was carried out using MestReNova software and manual line fitting was performed on monomer and polymer *methine* proton regions. Monomer percentage conversion was calculated as a relative ratio of the area under the fitted lines corresponding to monomer and polymer *methine* resonances.

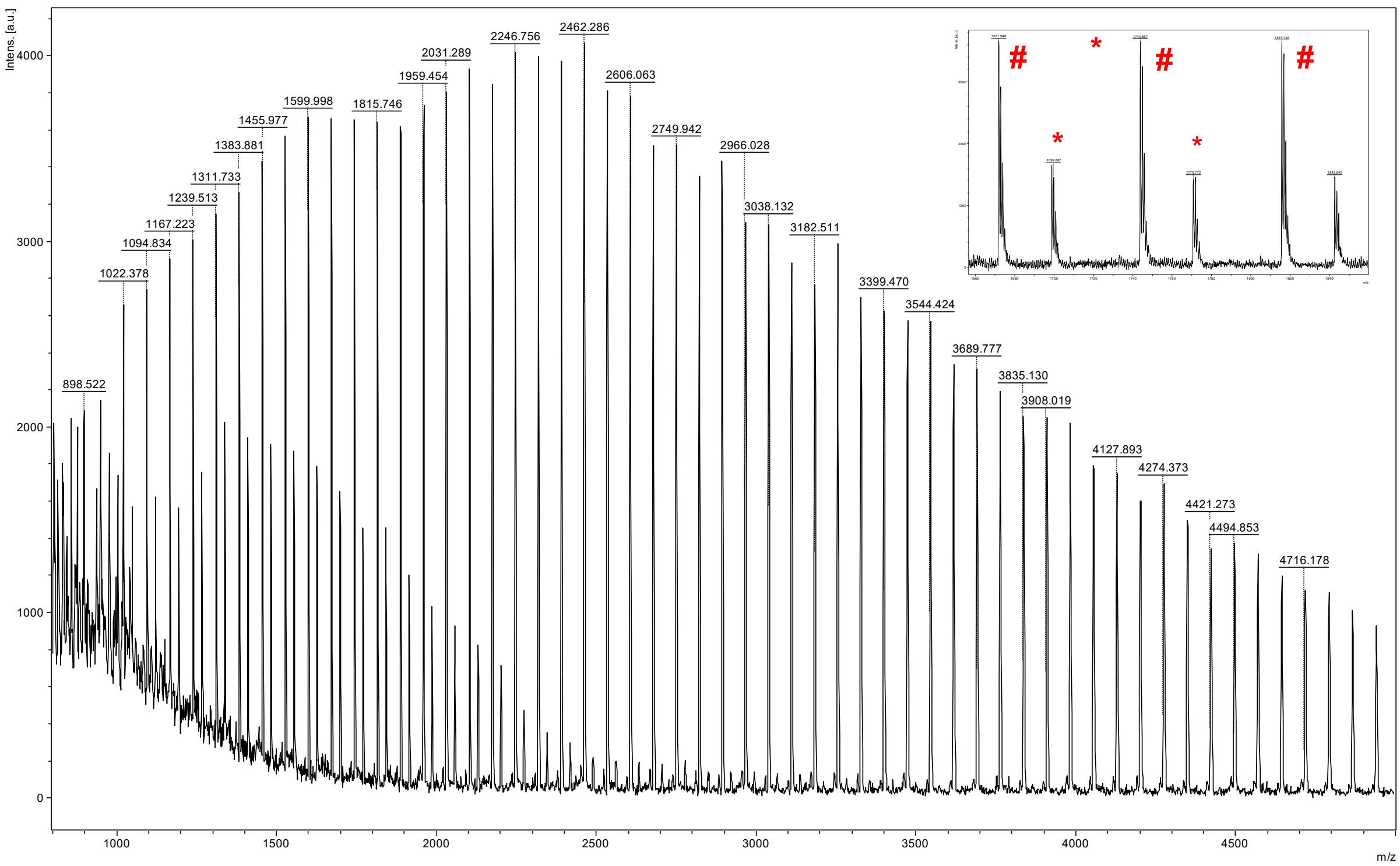

Figure S53: MALDI-ToF mass spectrum of the PLLA produced using **1**. Polymerisation conditions: benzene-d_6, 50 °C, [*L*-LA]$_0$/[Bi]$_0$ = 50:1, [*L*-LA]$_0$ = 0.5 M. # represents linear α–NMe$_2$/ω–OH terminated PLLA. * represents cyclic PLLA.

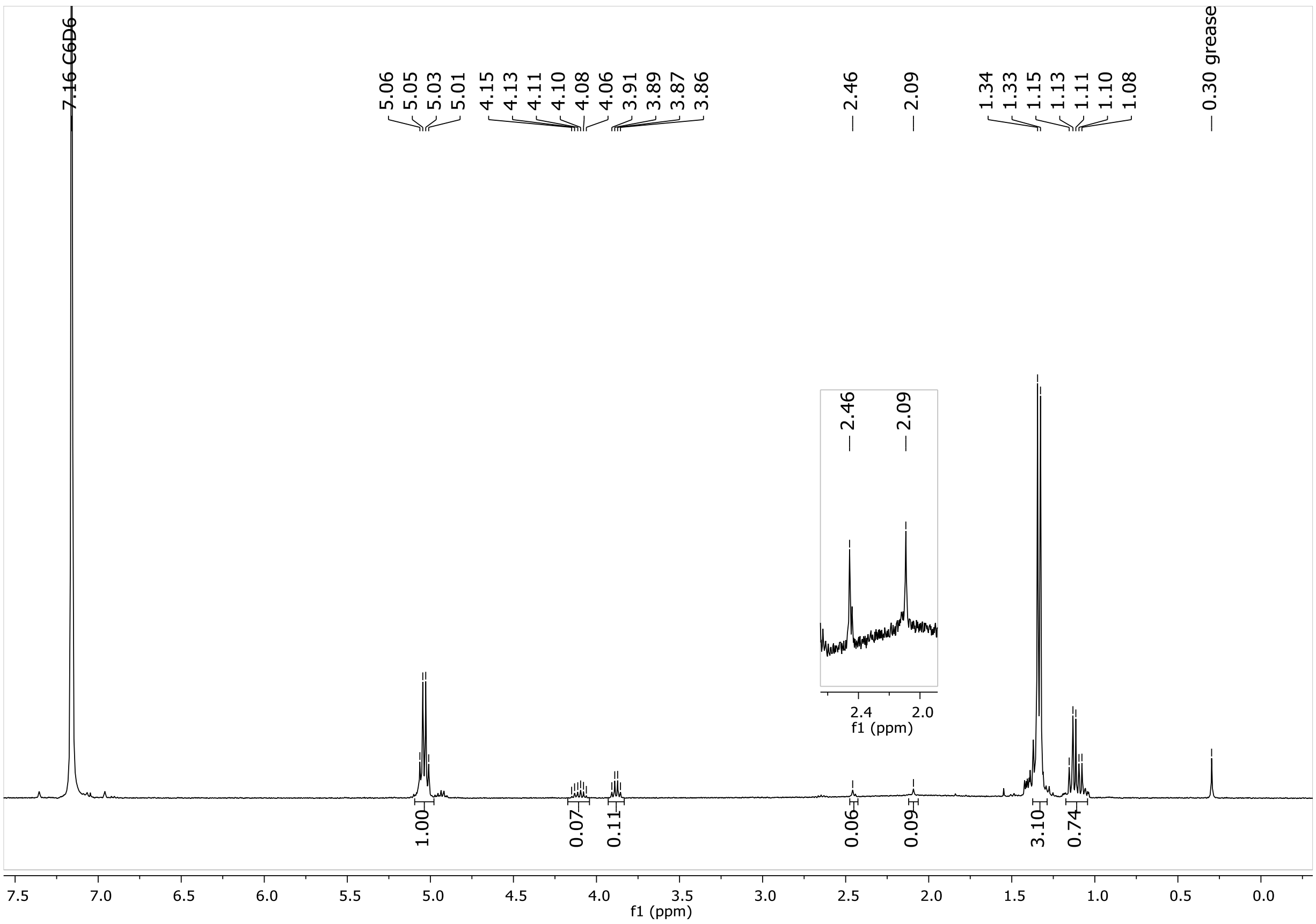

Figure S54: ^{1}H NMR spectrum (*benzene-d_6, 600 MHz) of the PLLA produced using **1**. Expanded region highlights terminal N*Me*$_2$ resonances. Polymerisation conditions: benzene-d_6, 50 °C, [*L*-LA]$_0$/[Bi]$_0$ = 50:1, [*L*-LA]$_0$ = 0.5 M.

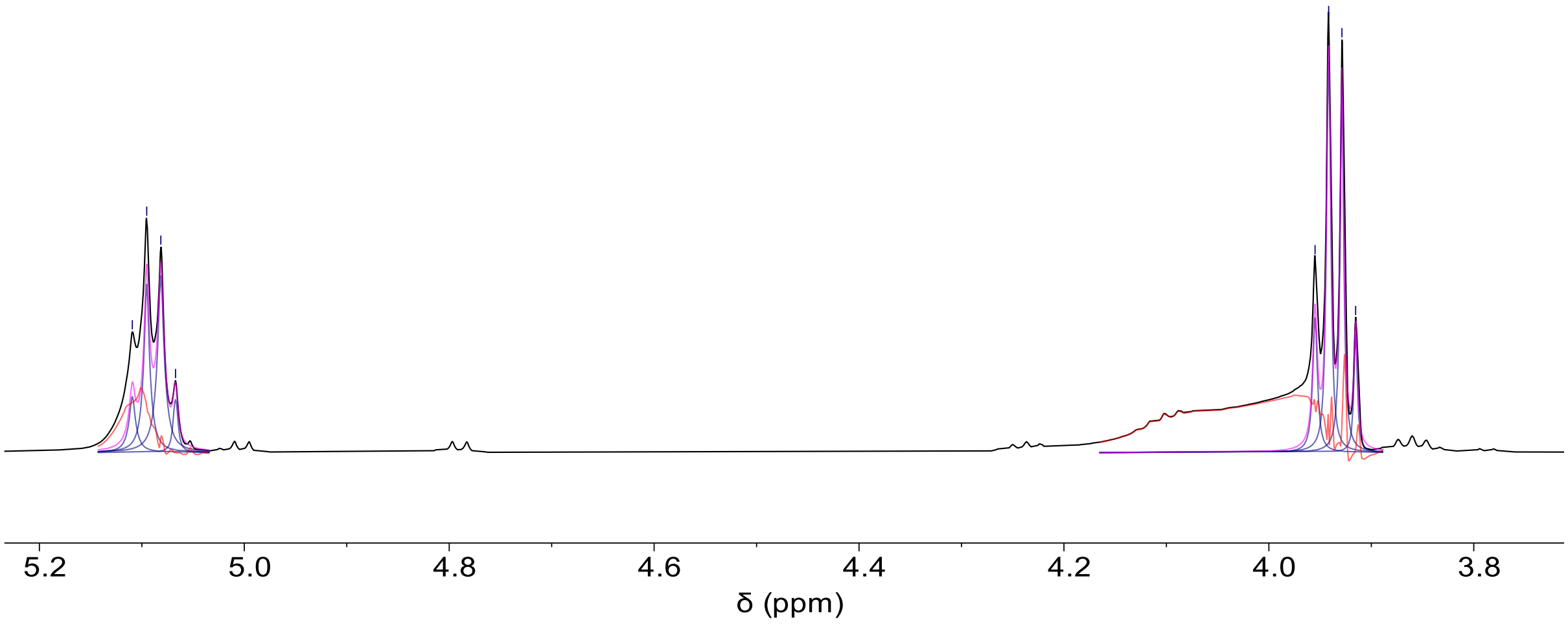

Figure S55: ^{1}H NMR spectrum (benzene-d_6, 600 MHz) for the polymerisation of *L*-lactide using **1**, demonstrating the deconvoluted *methine* proton regions. Polymerisation conditions: benzene-d_6, 50 °C, $[L\text{-LA}]_0/[\text{Bi}]_0$ = 50:1, $[L\text{-LA}]_0$ = 0.5 M.

3.10. ε-Caprolactone polymerisation using bismuth amide 1

Compound **1** was tested as an initiator for the ROP of ε-caprolactone. Polymerisation was carried out at 50°C with a $[\varepsilon\text{-CL}]_0:[\text{Bi}]_0$ ratio of 50:1, and an initial monomer concentration $[\varepsilon\text{-CL}]_0$ = 0.5 M. Unlike the ROP of *L*-lactide, ε-caprolactone was polymerised with an induction period of approximately 2 hours. A plot of $\ln([\varepsilon\text{-CL}]_0/[\varepsilon\text{-CL}]_t)$ *vs*. polymerisation time after the indication period showed a first-order dependence on monomer concentration, consistent with ROP proceeding *via* a typical coordination-insertion mechanism.[17] The activity of **1** in the polymerisation of ε-caprolactone was found to be more than three-fold lower than that for *L*-lactide (k_{obs} = 0.061 ± 0.003 and 0.219 ± 0.008 h^{-1}, respectively). The presence of the α–NMe_2 end group was observed in the ^{1}H NMR spectrum of the isolated PCL sample, with its two proton signals found at δ 2.67 and 2.20 ppm. Comparable NMR spectroscopic data were recorded in the case of α–NMe_2/ω–OH terminated PLLA (NMe_2 at δ 2.45 and 2.09 ppm). Analysis of the initial NMR spectrum after addition of monomer was consistent with the rapid formation of compound **3**, albeit the NMe_2 resonances were slightly broadened and the SiMe_2 resonances were slightly shifted relative to the isolated compound. These differences might be due to fluxional coordination of monomer to the bismuth centre of **3**. Thus, the induction period is likely to reflect low activity of the ring-opened alkoxy initiation product **3** towards further monomer insersion rather than slow reactivity of **1** towards monomer.

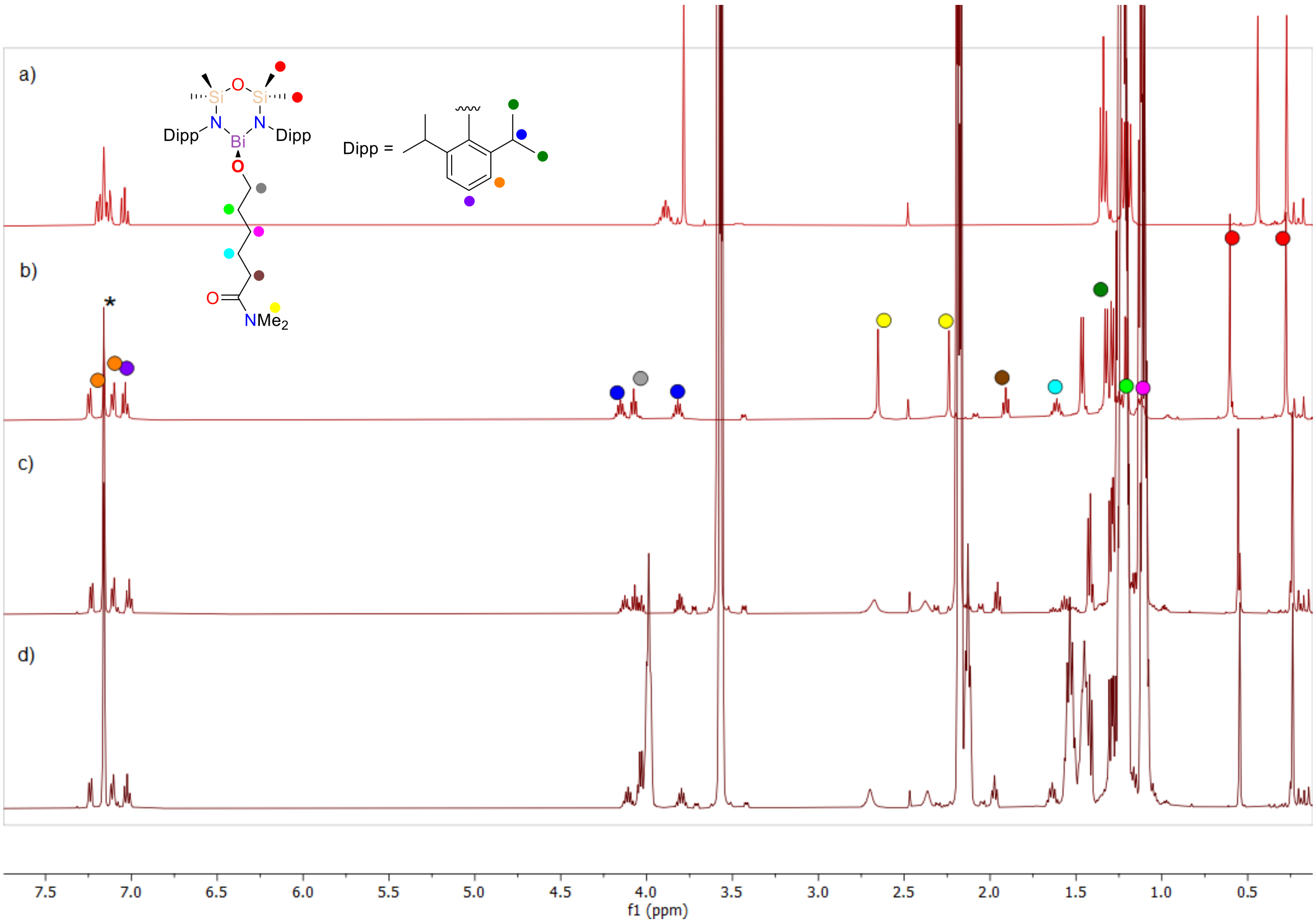

Figure S56: Stacked ^{1}H NMR spectra (400 MHz, benzene-*d*$_6$): a) compound **1**; b) compound **3**; c) initial polymerisation reaction mixture after addition of ε-CL to **1**, forming **3**; d) polymerisation mixture after 15 hours showing partial conversion of monomer, and formation of NMe_2, {(NON)Bi}-terminated PCL.

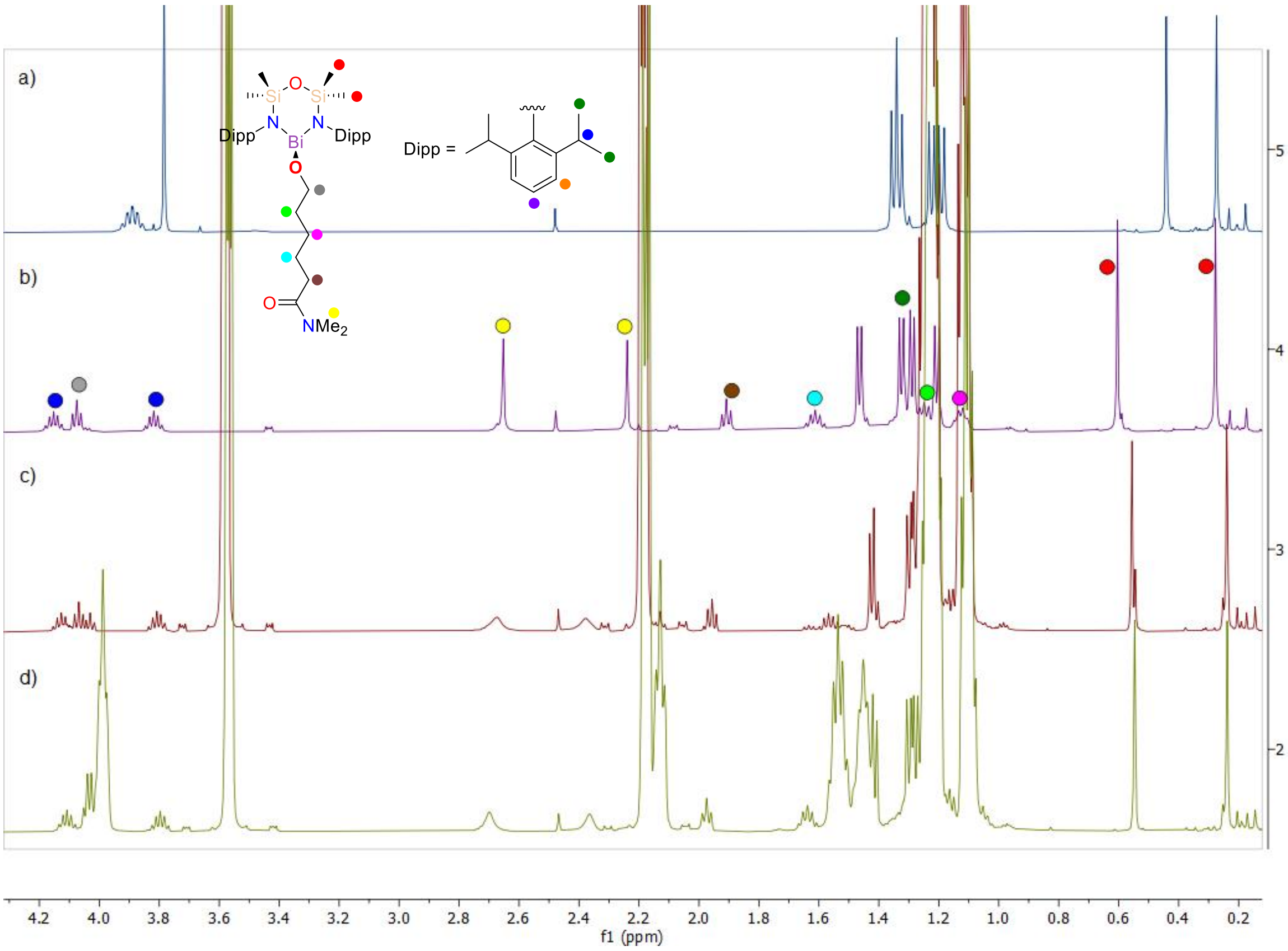

Figure S57: Expanded stacked ^{1}H NMR spectra (400 MHz, benzene-d_6) from top to bottom: compound **1**; compound **3**; initial polymerisation reaction mixture after addition of ε-CL to **1**, forming **3**; polymerisation mixture after 15 hours showing partial conversion of monomer, and formation of NMe_2, {(NON)Bi}-terminated PCL.

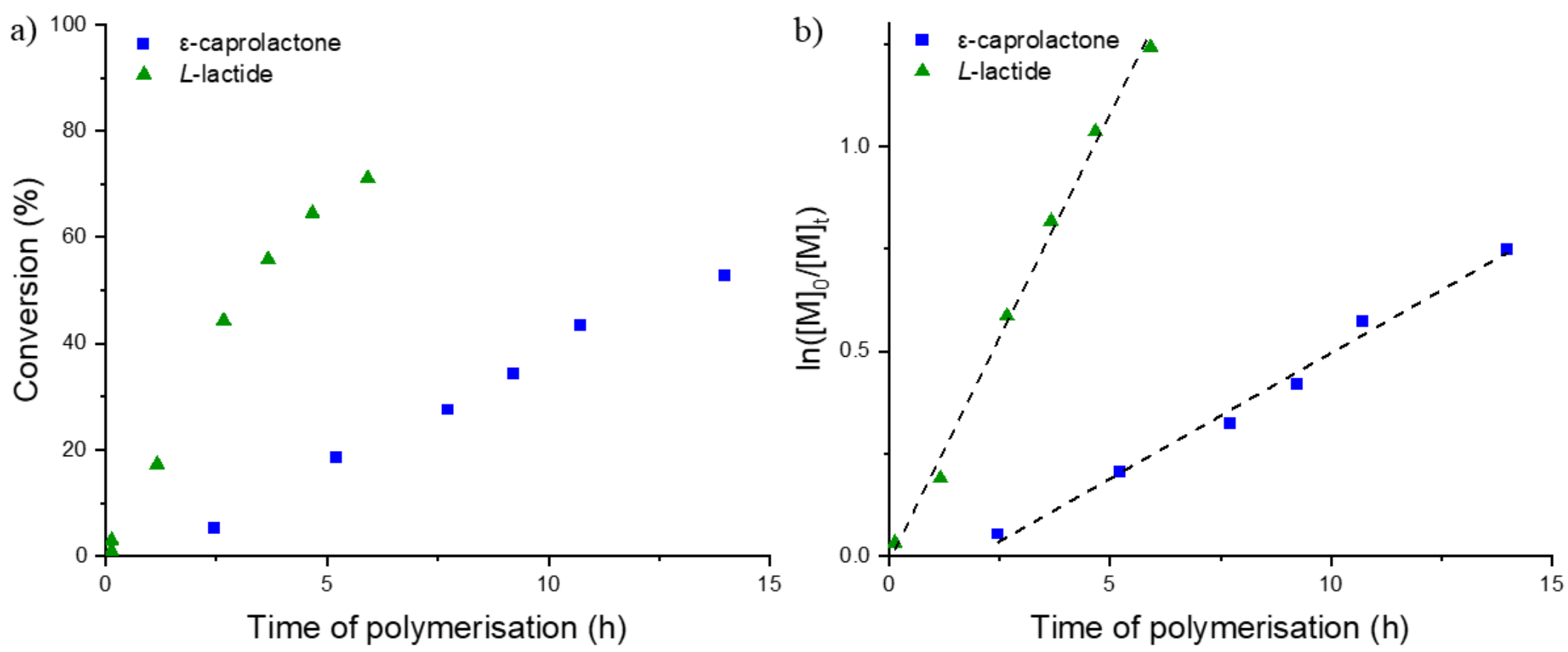

Figure S58: a) Plots of percentage conversion *vs.* polymerisation time; b) plots of $\ln([M]_0/[M]_t)$ *vs.* polymerisation time (where M = monomer). Blue squares: ε-CL, k_{obs} = 0.061 ± 0.003 h^{-1}, R^2 = 0.989; green up triangles: *L*-LA, k_{obs} = 0.219 ± 0.008 h^{-1}, R^2 = 0.994. Polymerisation conditions: benzene-d_6, 50 °C, $[M]_0/[Bi]_0$ = 50:1, and $[M]_0$ = 0.5 M.

Table S5: ROP of ε-caprolactone using **1**.[a]

**Conv.**[b] **(%)**	k_{obs}[c] **(h^{-1})**	R^{2c}	$M_{n,SEC}$[d] **(g mol^{-1})**	$M_{n,theo}$[e] **(g mol^{-1})**	M_w/M_n[d]
66	0.061 ± 0.003	0.989	2 930	3 812	1.32

[a]Polymerisation conditions: benzene-d_6, 50 °C, $[\varepsilon\text{-CL}]_0/[Bi]_0$ = 50:1, $[\varepsilon\text{-CL}]_0$ = 0.5 M. [b]Measured by ^1H NMR spectroscopy. [c]Obtained from plots of $\ln([\varepsilon\text{-CL}]_0/[\varepsilon\text{-CL}]_t)$ *vs.* polymerisation time. [d]Determined by SEC analysis calibrated with PS standards in THF with the appropriate Mark-Houwink corrections.[3] [e]Theoretical M_n for PCL = (conv.(%) × $[\varepsilon\text{-CL}]_0/[Bi]_0$) × 114.14 + ($M_w$ of end groups).

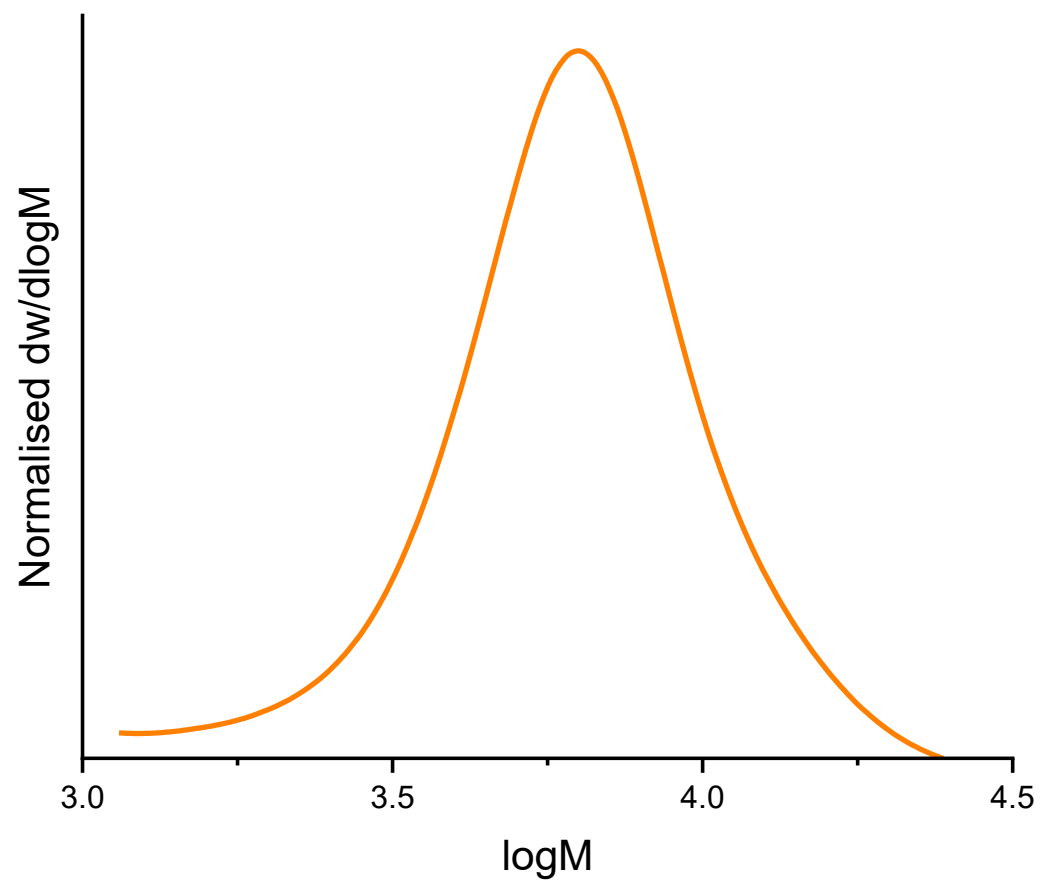

Figure S59: SEC plot of the PCL produced using **1** (M_n = 2 900 g mol^{-1}, $Đ_M$ = 1.32**).** Polymerisation conditions: benzene-d_6, 50 °C, $[\varepsilon\text{-CL}]_0/[Bi]_0$ = 50:1, and $[\varepsilon\text{-CL}]_0$ = 0.5 M.

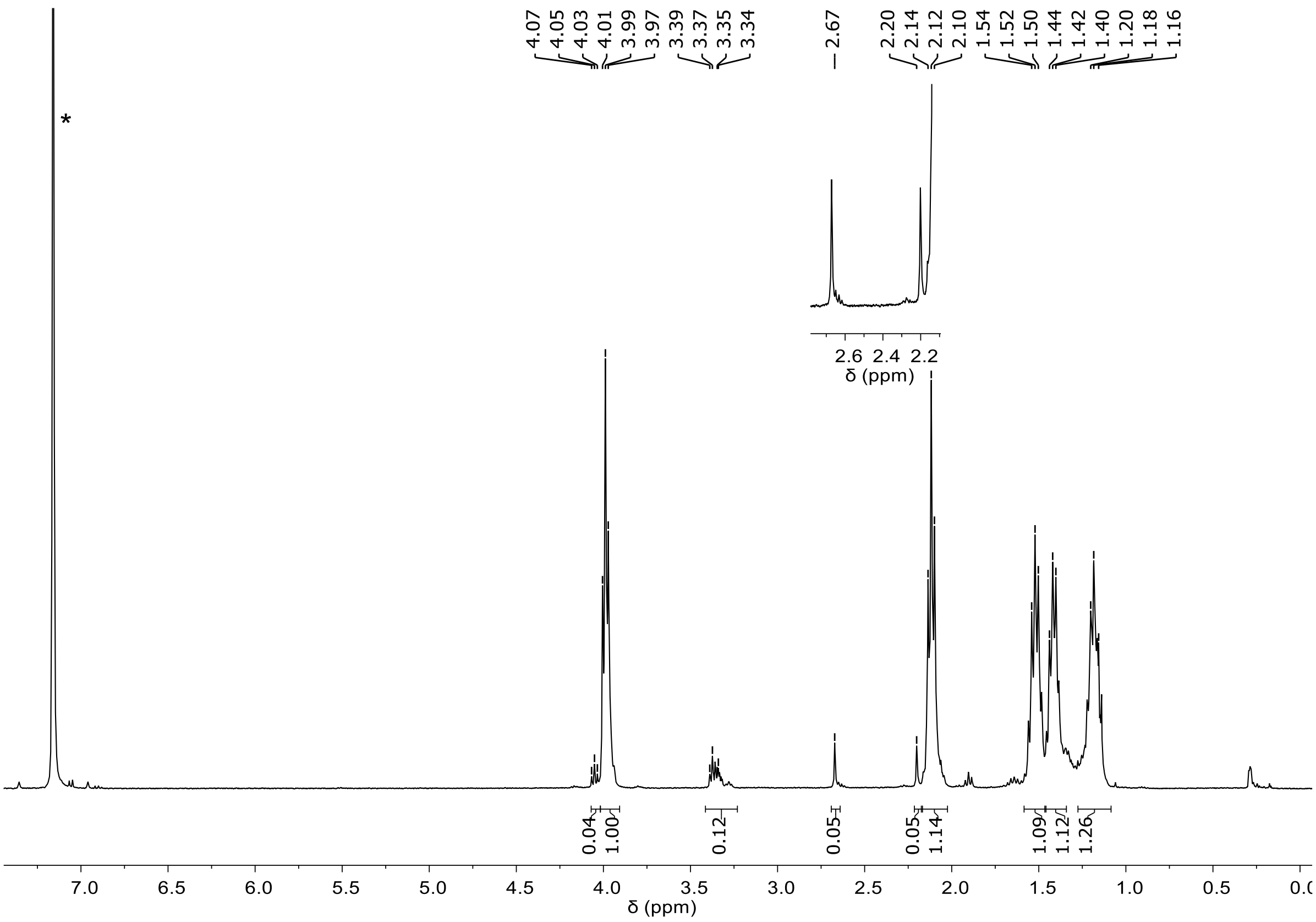

Figure S60: ^{1}H NMR spectrum (*benzene-d_6, 600 MHz) of the PCL produced using **1**. Expanded region highlights the N*Me*$_2$ end group. Polymerisation conditions: benzene-d_6, 50 °C, $[\varepsilon\text{-CL}]_0/[\text{Bi}]_0$ = 50:1, $[\varepsilon\text{-CL}]_0$ = 0.5 M.

4. Crystallographic data

4.1. General considerations, crystal data and refinement

Crystals were mounted on MiTeGen MicroMounts using perfluoropolyether oil and rapidly transferred to a goniometer head on a diffractometer fitted with an Oxford Cryostream open-flow nitrogen cooling device. Data collections for compounds **2** and **4** were conducted at 150 K using an Oxford Diffraction Supernova diffractometer with mirror monochromated Cu Kα radiation (λ = 1.54178 Å), whilst that for compound **1** was conducted at 150 K using an Oxford Diffraction XtaLAB Synergy, Dualflex, HyPix-Arc 100 diffractomter with Mo Kα radiation (λ = 0.71073 Å). The data were processed using CrysAlisPro.[18] Using Olex2,[19] the structure was solved with the SHELXT[20] structure solution program using Intrinsic Phasing and refined with the SHELXL[21] refinement package using Least Squares minimisation.

Table S6: Crystal data and structure refinement.

Identification code	**1**	**2**	**4**
Empirical formula	$C_{30}H_{52}BiN_3OSi_2$	$C_{33}H_{56}BiN_3O_3Si_2$	$C_{34}H_{58}BiN_3O_3Si_2$
Formula weight	735.90	807.96	821.99
Temperature/K	150.0(2)	150.0(2)	150.15
Crystal system	monoclinic	orthorhombic	triclinic
Space group	$P2_1/c$	$P2_12_12_1$	P-1
a/Å	12.3332(3)	9.79450(10)	9.85973(16)
b/Å	19.4335(4)	15.49700(10)	12.13718(19)
c/Å	14.5413(3)	24.3631(2)	17.0845(3)
α/°	90	90	71.1125(15)
β/°	108.889(2)	90	80.6848(14)
γ/°	90	90	77.9477(13)
Volume/Å^3	3297.53(13)	3697.96(5)	1881.92(6)
Z	4	4	2
ρ_{calc}g/cm^3	1.482	1.451	1.451
μ/mm^{-1}	5.445	10.241	10.071
F(000)	1488.0	1640.0	836.0
Crystal size/mm^3	0.29 × 0.06 × 0.04	0.54 × 0.07 × 0.06	0.218 × 0.141 × 0.091
Radiation	Mo Kα (λ = 0.71073)	Cu Kα (λ = 1.54184)	CuKα (λ = 1.54184)
2Θ range for data collection/°	4.318 to 62.17	6.76 to 152.37	7.81 to 152.396
Index ranges	-17 ≤ h ≤ 16, -26 ≤ k ≤ 26, -20 ≤ l ≤ 19	-12 ≤ h ≤ 11, -19 ≤ k ≤ 19, -30 ≤ l ≤ 30	-12 ≤ h ≤ 12, -15 ≤ k ≤ 15, -21 ≤ l ≤ 20
Reflections collected	49277	55520	37616
Independent reflections	8968 [R_{int} = 0.0493, R_{sigma} = 0.0409]	7704 [R_{int} = 0.0515, R_{sigma} = 0.0256]	7805 [R_{int} = 0.0504, R_{sigma} = 0.0296]
Data/restraints/parameters	8968/0/357	7704/0/394	7805/1896/773
Goodness-of-fit on F^2	1.012	1.057	1.084
Final R indexes [I>=2σ (I)]	R_1 = 0.0268, wR_2 = 0.0448	R_1 = 0.0208, wR_2 = 0.0462	R_1 = 0.0288, wR_2 = 0.0747
Final R indexes [all data]	R_1 = 0.0433, wR_2 = 0.0474	R_1 = 0.0243, wR_2 = 0.0481	R_1 = 0.0296, wR_2 = 0.0754
Largest diff. peak/hole / e Å^{-3}	0.53/-0.55	0.90/-0.82	1.75/-0.83
Flack parameter		-0.043(4)	

4.2. Crystal structure determination of [(NON^{dipp})Bi(NMe_2)] (1)

CCDC Deposition Number 2580882.

Crystal Data for $C_{30}H_{52}BiN_3OSi_2$ (M =735.90 g/mol): monoclinic, space group $P2_1/c$ (no. 14), a = 12.3332(3) Å, b = 19.4335(4) Å, c = 14.5413(3) Å, β = 108.889(2)°, V = 3297.53(13) Å^3, Z = 4, T = 150.00(10) K, μ(Mo Kα) = 5.445 mm^{-1}, $Dcalc$ = 1.482 g/cm^3, 49277 reflections measured (4.318° ≤ 2Θ ≤ 62.17°), 8968 unique (R_{int} = 0.0493, R_{sigma} = 0.0409) which were used in all calculations. The final R_1 was 0.0268 (I > 2σ(I)) and wR_2 was 0.0474 (all data).

Residual electron density was found 0.73 Å from Bi1 and was accounted for by splitting the bismuth centre into two components (Bi1, Bi1A) of relative respective occupancies of 0.9838/0.0162. No other atoms belonging to the latter component were located or modelled, presumably due to very low occupancy.

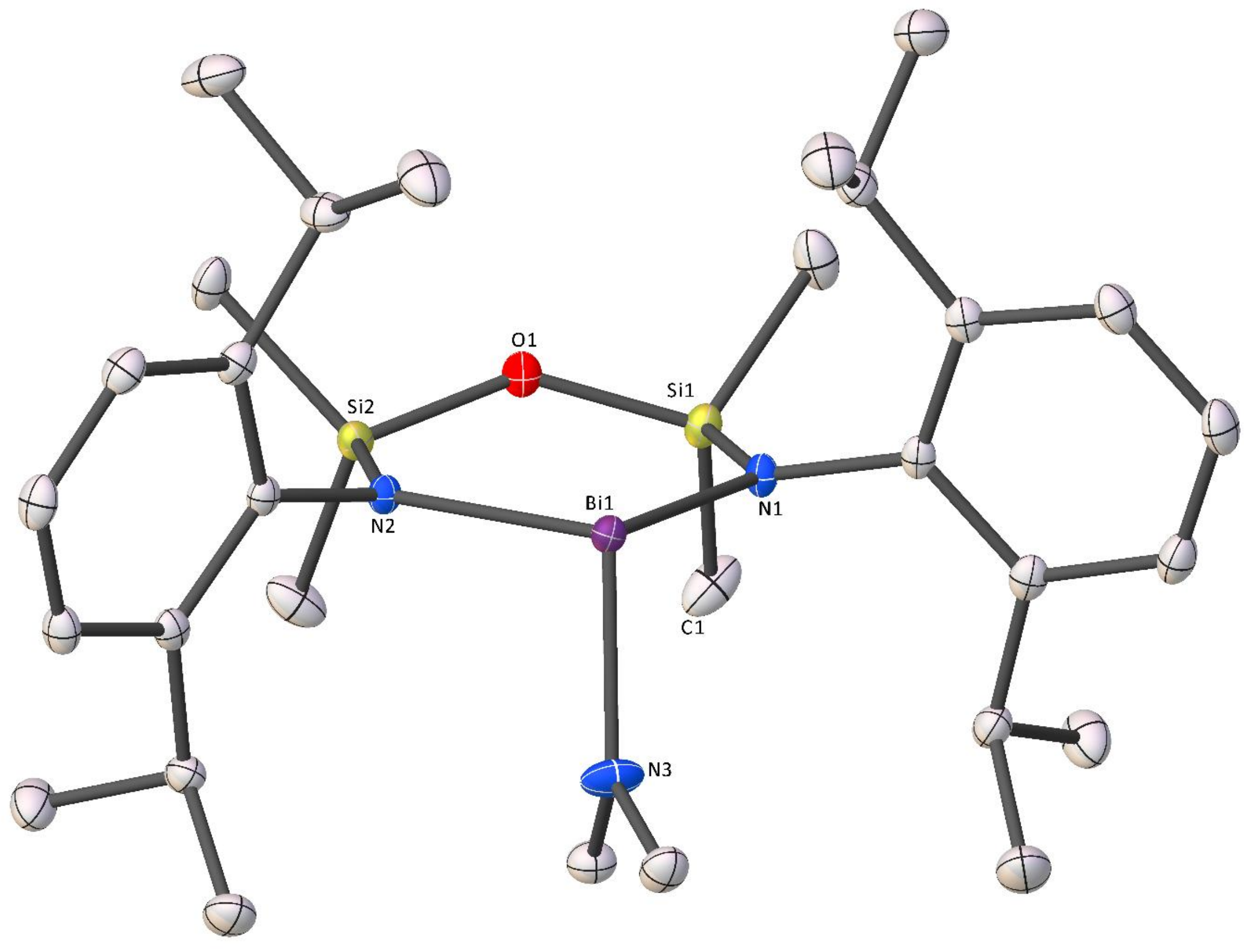

Figure S61: Displacement ellipsoid plot (30%) of the X-ray crystal structure of **1**. Hydrogen atoms are omitted for clarity. Selected distances (Å) and angles (°): Bi1-N1, 2.193(2); Bi1-N2, 2.188(2); Bi1-N3 2.112(2); N1-Bi1-N2, 99.33(7); N1-Bi2-N3, 99.99(9); N2-Bi1-N3, 98.03(9).

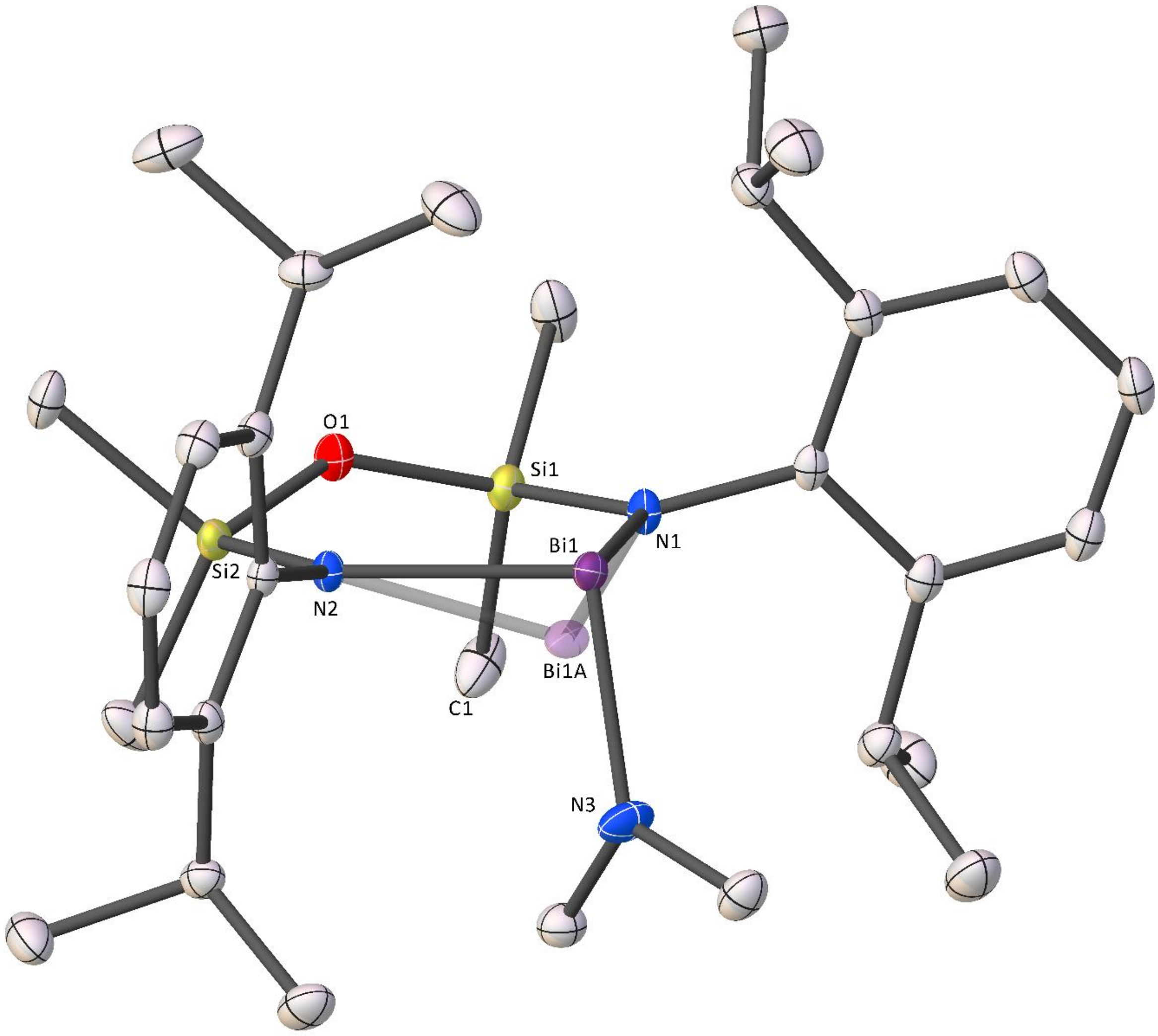

Figure S62: Displacement ellipsoid plot (30%) of the X-ray crystal structure of **1**, showing the minor component (occupancy 0.01628) of the split bismuth centre. Hydrogen atoms are omitted for clarity.

4.3. Crystal structure determination of [(NON^{dipp})Bi(κ^2-*O*,*O*-OC(CH$_3$)HC(O)NMe$_2$)] (2)

CCDC Deposition Number 2580883.

Crystal Data for $C_{33}H_{56}BiN_3O_3Si_2$ (*M* =807.96 g/mol): orthorhombic, space group $P2_12_12_1$ (no. 19), *a* = 9.79450(10) Å, *b* = 15.49700(10) Å, *c* = 24.3631(2) Å, *V* = 3697.96(5) Å^3, *Z* = 4, *T* = 150.0(2) K, μ(Cu Kα) = 10.241 mm^{-1}, *Dcalc* = 1.451 g/cm^3, 55520 reflections measured (6.76° ≤ 2Θ ≤ 152.37°), 7704 unique (R_{int} = 0.0515, R_{sigma} = 0.0256) which were used in all calculations. The final R_1 was 0.0208 (I > 2σ(I)) and wR_2 was 0.0481 (all data).

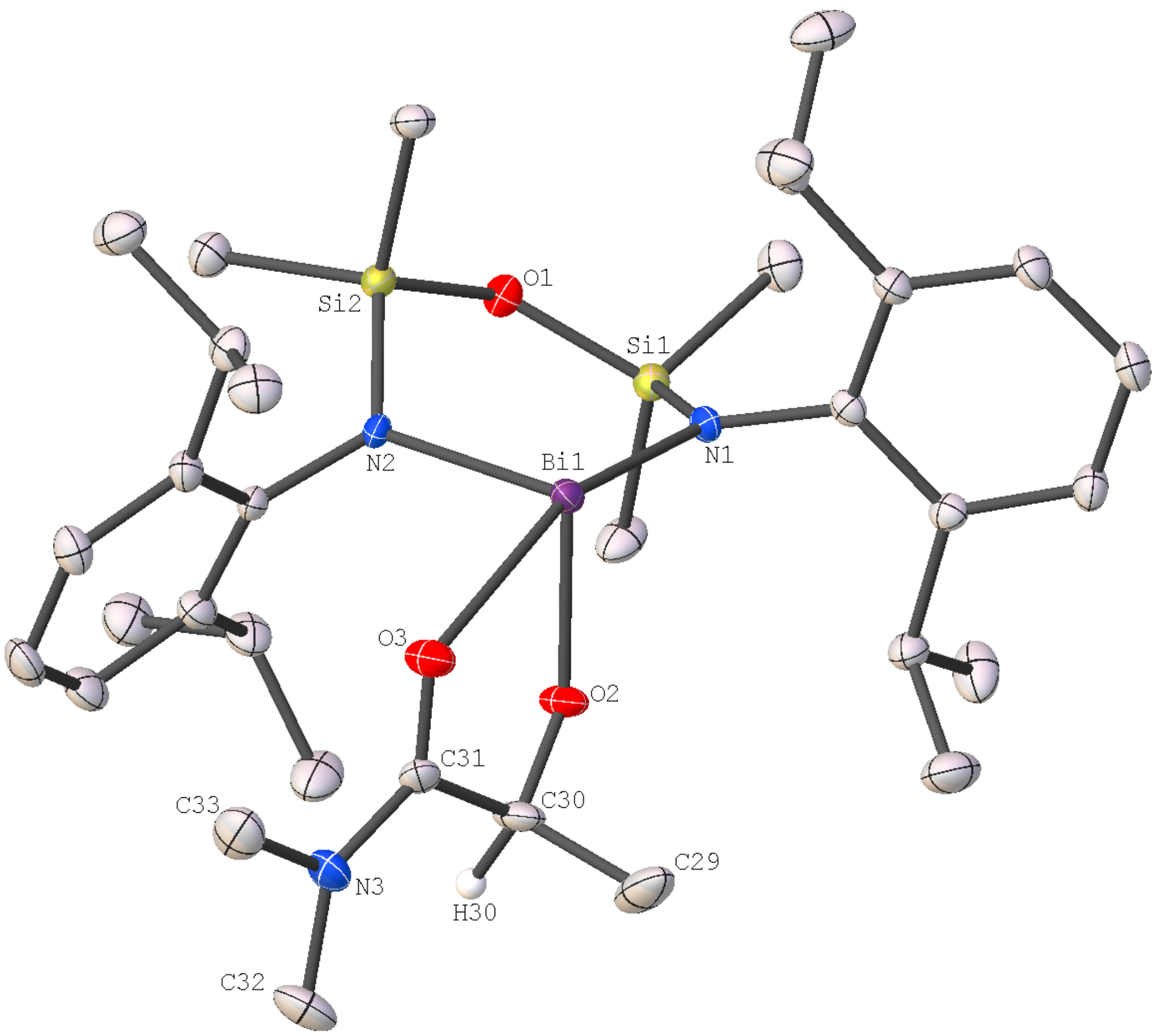

Figure S63: Displacement ellipsoid plot (30%) of the X-ray crystal structure of **2**. Hydrogen atoms are omitted for clarity except for H30. Selected distances (Å) and angles (°): Bi1-N1, 2.193(3); Bi1-N2, 2.184(3); Bi1-O2, 2.111(3); Bi1-O3, 2.590(3); C30-O2, 1.401(6); C31-O3, 1.240(6), C31-N3; N1-Bi1-N2, 94.12(14); N1-Bi2-O2 92.06(15); N2-Bi1-O2 94.75(14); N1-Bi1-O3 161.29(12); N2-Bi1-O3 92.21(14); O2-Bi1-O3 69.86(12); N3-C31-O3 121.5(5); O3-C31-C30 118.5(5); C30-C31-N3 119.9(5).

4.4. Crystal structure determination of [(NON^{dipp})Bi(O(CH$_2$)$_3$C(O)NMe$_2$)] (4)

CCDC Deposition Number 2580884.

Crystal Data for $C_{34}H_{58}BiN_3O_3Si_2$ (*M* =821.99 g/mol): triclinic, space group P-1 (no. 2), *a* = 9.85973(16) Å, *b* = 12.13718(19) Å, *c* = 17.0845(3) Å, *α* = 71.1125(15)°, *β* = 80.6848(14)°, *γ* = 77.9477(13)°, *V* = 1881.92(6) Å^3, *Z* = 2, *T* = 150.15 K, μ(CuKα) = 10.071 mm^{-1}, *Dcalc* = 1.451 g/cm^3, 37616 reflections measured (7.81° ≤ 2Θ ≤ 152.396°), 7805 unique (R_{int} = 0.0504, R_{sigma} = 0.0296) which were used in all calculations. The final R_1 was 0.0288 (I > 2σ(I)) and wR_2 was 0.0754 (all data).

In the crystal structure of **4**, the asymmetric unit contains one bismuth complex monomer. The entire molecule was found to be disordered, such that the structure solution was refined using a two-component split model with relative occupancy of 0.55:0.45. Distance and ADP restraints were employed to assist with modelling the disorder. In the major component, part 1, short (2.797(5) Å) intermolecular Bi1A-O3A interactions lead to a macrocyclic dimeric motif, whilst the minor component, part 2, exhibits longer (3.339(9) Å) intermolecular Bi1B-O3B interactions to give an extended polymeric chain structure. Intermolecular π-π stacking interactions between amide groups also contribute to the solid-state packing.

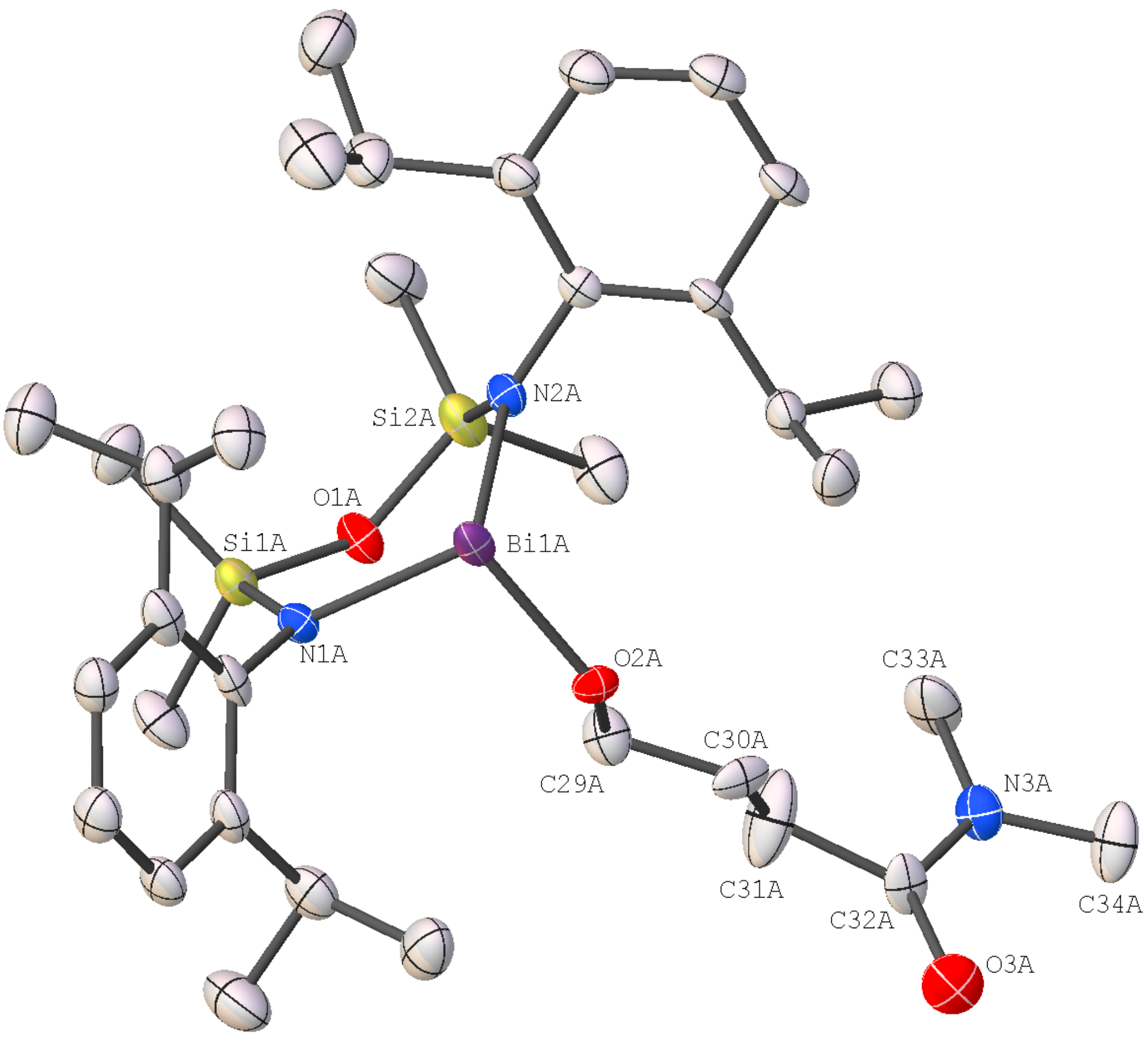

Figure S64: Displacement ellipsoid plot (30%) of the asymmetric unit of the major component (55%) present in the X-ray crystal structure of **4**. Hydrogen atoms are omitted for clarity.

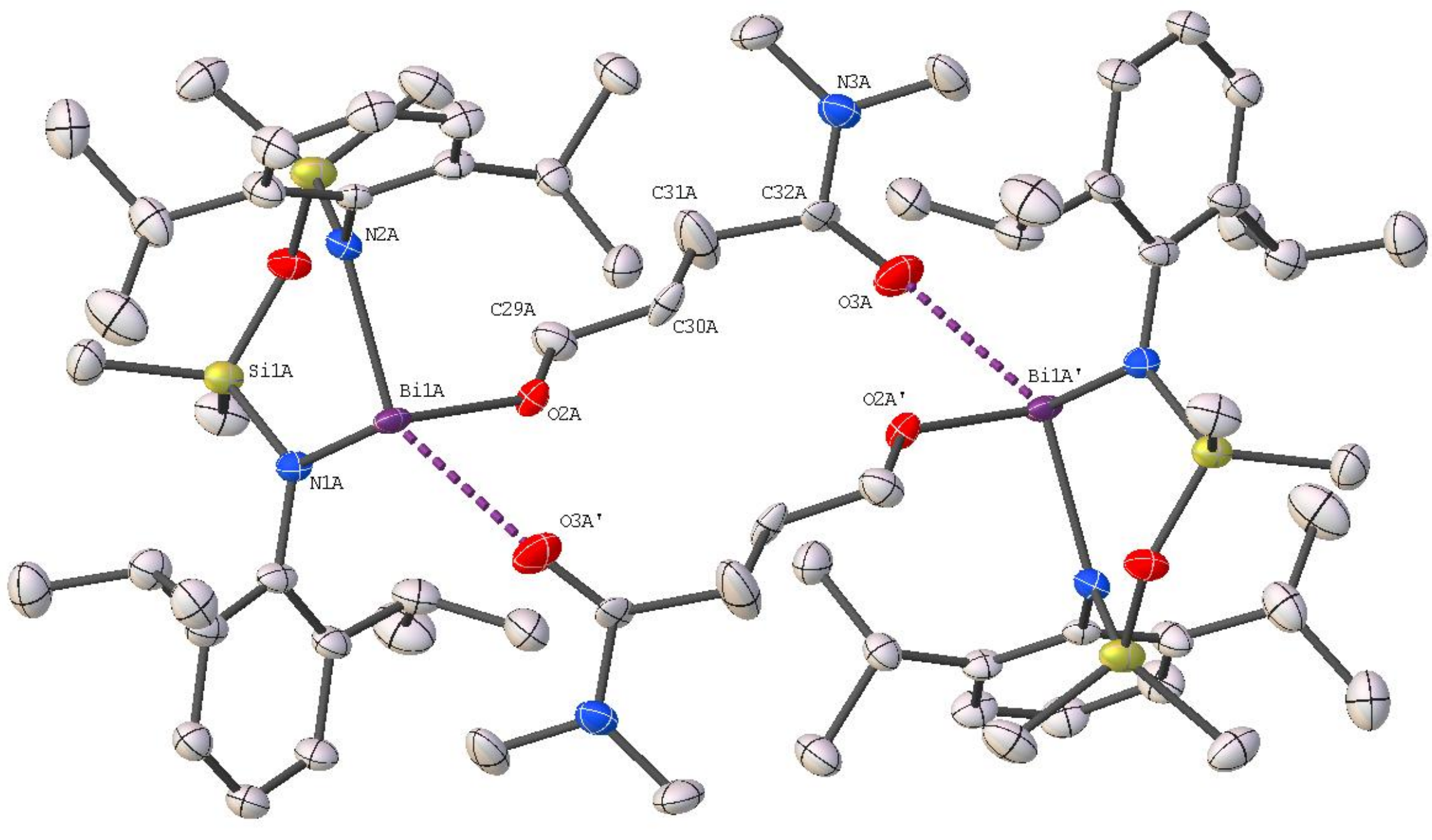

Figure S65: Displacement ellipsoid plot (30%) of the centrosymmetric bimolecular dimer present as the major component (55%) in the X-ray crystal structure of **4**. Hydrogen atoms are omitted for clarity.

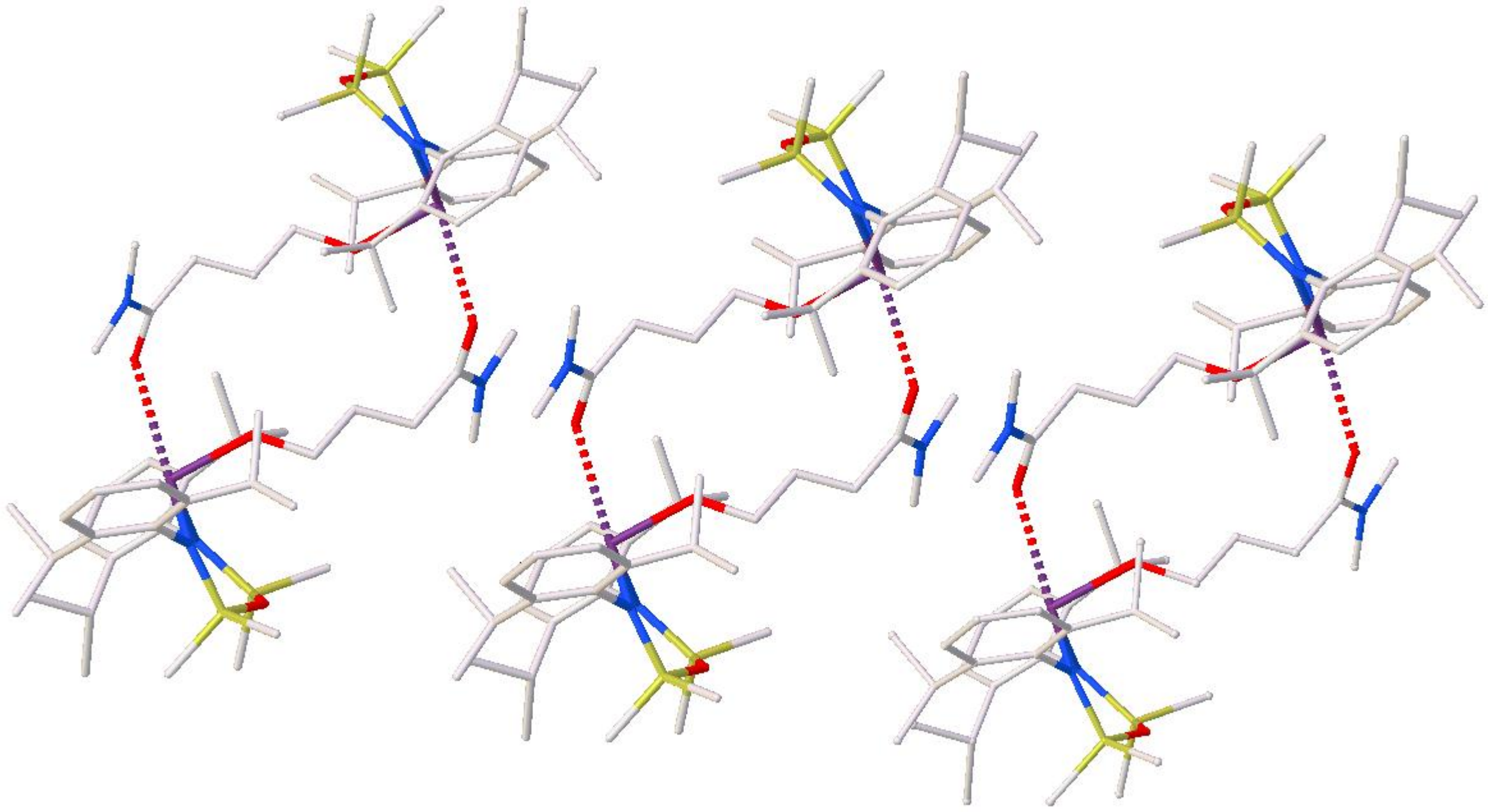

Figure S66: Wireframe plot showing the packing of the dimeric major component (55%) in the X-ray crystal structure of **4**. Hydrogen atoms are omitted for clarity.

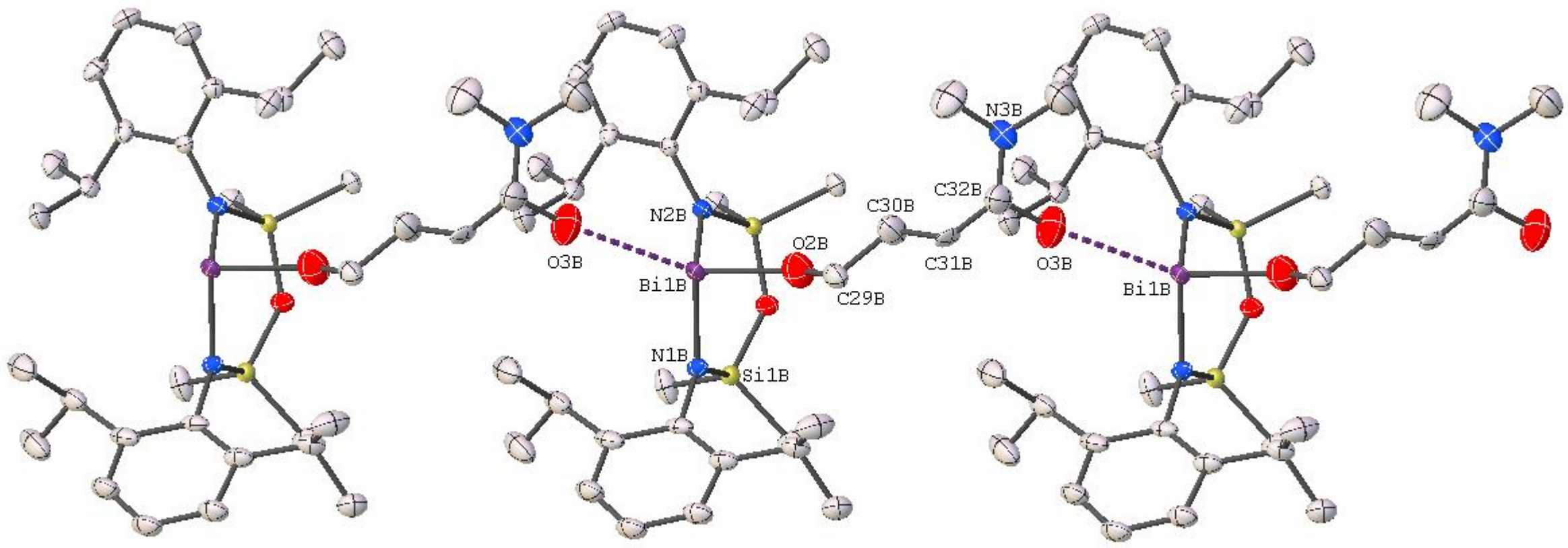

Figure S67: Displacement ellipsoid plot (30%) of the extended polymeric chain structure (three repeat units) present as the minor component (45%) in the X-ray crystal structure of **4**. Hydrogen atoms are omitted for clarity.

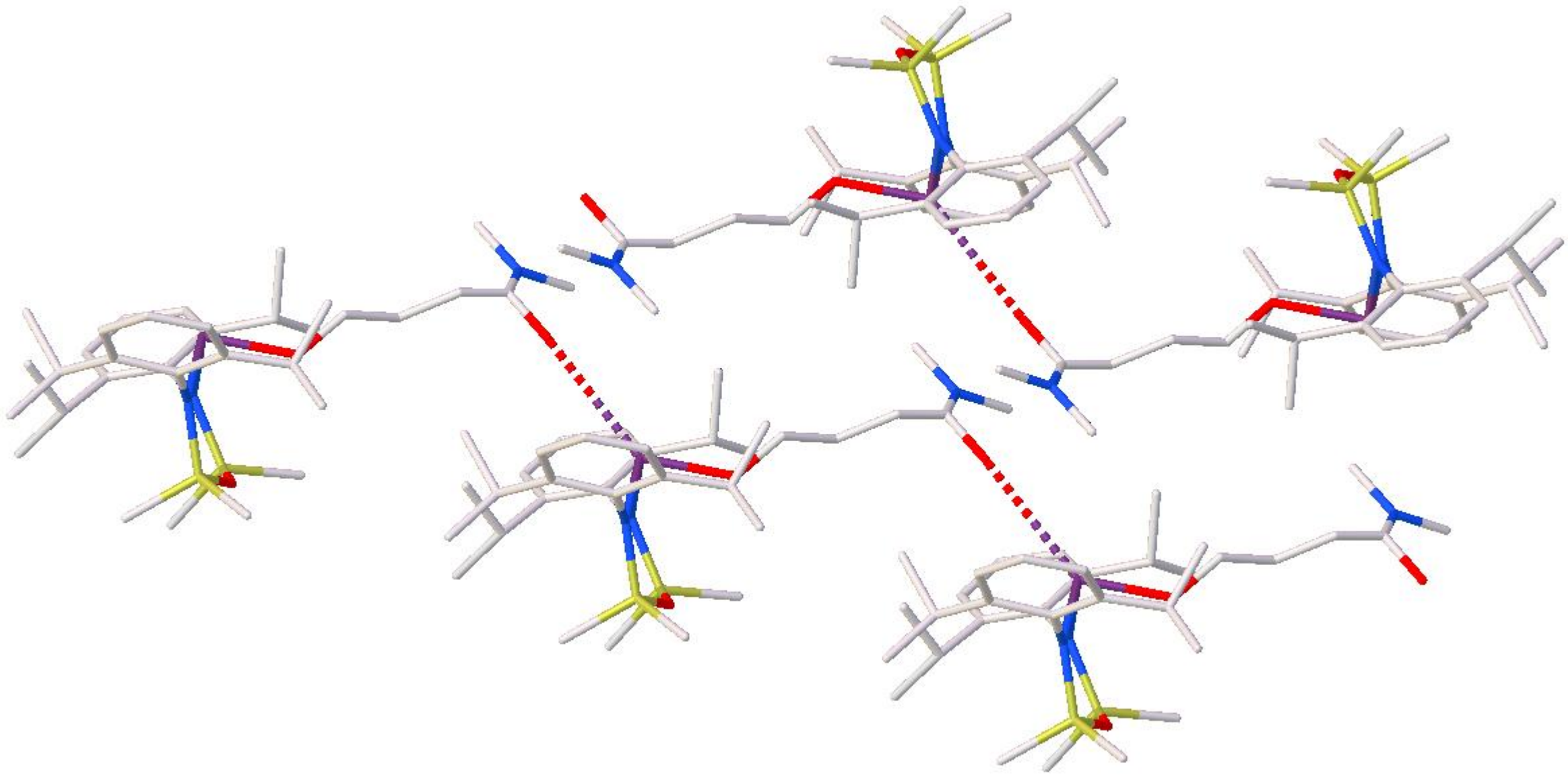

Figure S68: Wireframe plot showing the packing of the polymeric chain-structured minor component (45%) in the X-ray crystal structure of **4**. Hydrogen atoms are omitted for clarity.

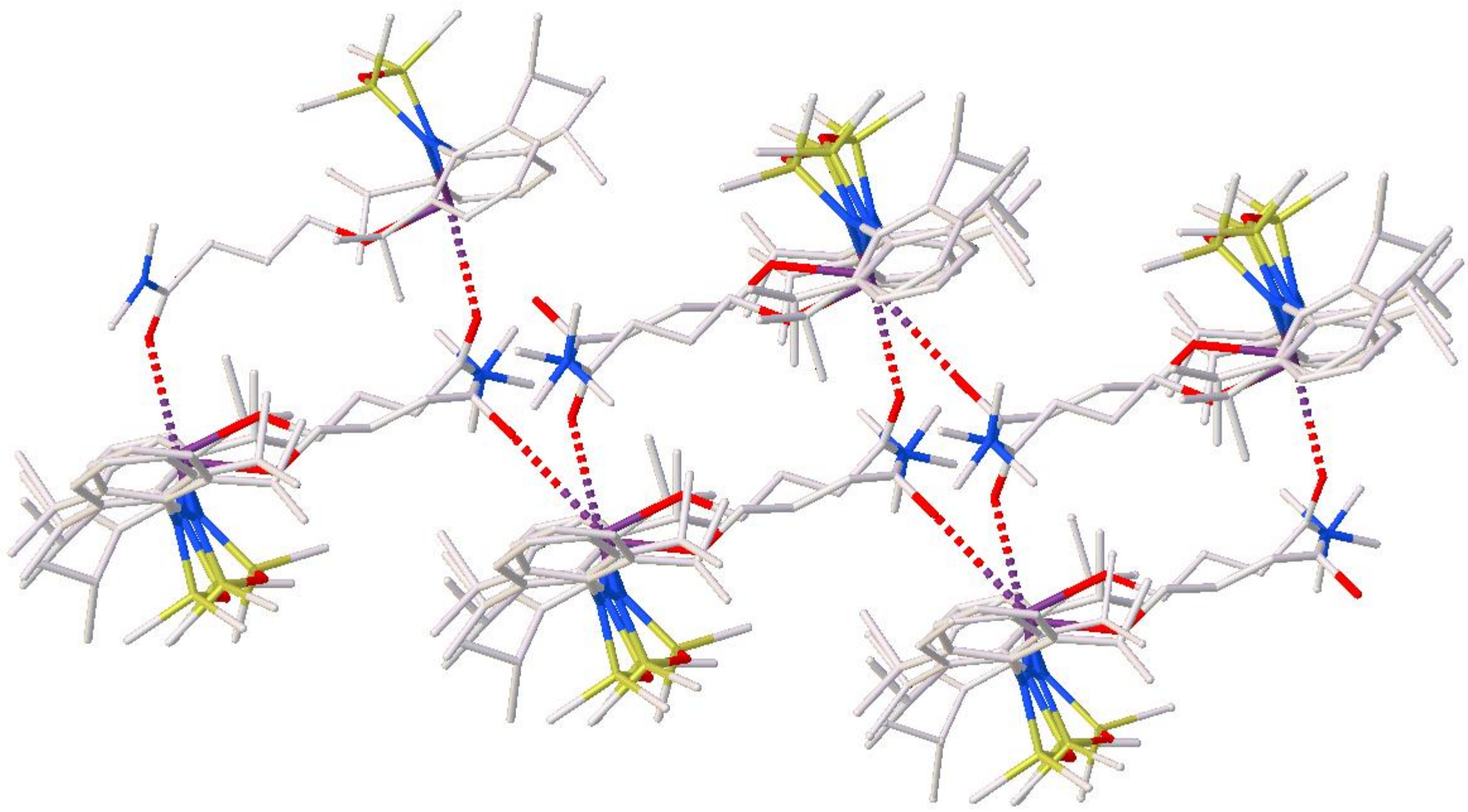

Figure S69: Wireframe plot illustrating the packing of both components of the disordered X-ray crystal structure of **4.** Hydrogen atoms are omitted for clarity.

5. References

1. W. Clegg, N. A. Compton, R. J. Errington, G. A. Fisher, M. E. Green, D. C. R. Hockless and N. C. Norman, *Inorganic Chemistry*, 1991, **30**, 4680-4682.
2. D. B. Leznoff, G. Mund, K. C. Jantunen, P. H. Bhatia, A. J. Gabert and R. J. Batchelor, *Journal of Nuclear Science and Technology*, 2002, **39**, 406-409.
3. J. R. Dorgan, J. Janzen, D. M. Knauss, S. B. Hait, B. R. Limoges and M. H. Hutchinson, *J. Polym. Sci., Part B: Polym. Phys.*, 2005, **43**, 3100-3111.
4. M. Haddad, M. Laghzaoui, R. Welter and S. Dagorne, *Organometallics*, 2009, **28**, 4584-4592.
5. J. Coudane, C. Ustariz-Peyret, G. Schwach and M. Vert, *J. Polym. Sci., Part A: Polym. Chem.*, 1997, **35**, 1651-1658.

6. A. P. Dove, V. C. Gibson, E. L. Marshall, H. S. Rzepa, A. J. P. White and D. J. Williams, *J. Am. Chem. Soc.*, 2006, **128**, 9834-9843.
7. J.-C. Buffet and J. Okuda, *Polym. Chem.*, 2011, **2**, 2758-2763.
8. H. R. Kricheldorf and C. Boettcher, *J. Macromol. Sci. A*, 1993, **30**, 441-448.
9. Z. R. Turner, J. T. Wilmore, N. H. Rees and J.-C. Buffet, *Dalton Trans.*, 2022, **51**, 3060-3074.
10. H.-Y. Chen, J. Zhang, C.-C. Lin, J. H. Reibenspies and S. A. Miller, *Green Chem.*, 2007, **9**, 1038-1040.
11. B. M. Chamberlain, M. Cheng, D. R. Moore, T. M. Ovitt, E. B. Lobkovsky and G. W. Coates, *J. Am. Chem. Soc.*, 2001, **123**, 3229-3238.
12. C. K. Williams, L. E. Breyfogle, S. K. Choi, W. Nam, V. G. Young, M. A. Hillmyer and W. B. Tolman, *J. Am. Chem. Soc.*, 2003, **125**, 11350-11359.
13. A. Kowalski, J. Libiszowski, A. Duda and S. Penczek, *Macromolecules*, 2000, **33**, 1964-1971.
14. N. Diteepeng, J.-C. Buffet, Z. R. Turner and D. O'Hare, *Dalton Trans.*, 2019, **48**, 16099-16107.
15. A. F. Douglas, B. O. Patrick and P. Mehrkhodavandi, *Angew. Chem. Int. Ed.*, 2008, **47**, 2290-2293.
16. G. Hu, Z. Deng, Y. Wang, D. Yuan, Y. Zhang and Y. Yao, *J. Organomet. Chem.*, 2024, **1003**, 122941.
17. P. Dubois, N. Ropson, R. Jérôme and P. Teyssié, *Macromolecules*, 1996, **29**, 1965-1975.
18. *CrysAlisPRO*, Oxford Diffraction / Agilent Technologies UK Ltd, 2013.
19. O. V. Dolomanov, L. J. Bourhis, R. J. Gildea, J. A. K. Howard and H. Puschmann, *J. Appl. Cryst.*, 2009, **42**, 339-341.
20. G. M. Sheldrick, *Acta Cryst. A*, 2015, **71**, 3-8.
21. G. M. Sheldrick, *Acta Cryst. C*, 2015, **71**, 3-8.
